# Supplementary material for: Clinical utility of foundation models in musculoskeletal MRI for biomarker fidelity and predictive outcomes
Source: NPJ Digit Med. 2026 Mar 24;9:383. doi: 10.1038/s41746-026-02520-w (PMC13187405; doi:10.1038/s41746-026-02520-w)
Supplement: Supplementary file 1 — Supplementary Information [file 41746_2026_2520_MOESM1_ESM.pdf]

Supplementary Information for: Clinical utility of foundation models in musculoskeletal MRI for biomarker fidelity and predictive outcomes

Gabrielle Hoyer[1,2,3,*], Michelle W Tong[1,2,3], Rupsa Bhattacharjee[1,4], Valentina Pedoia[1,5], Sharmila Majumdar[1,3]

[1] Center for Intelligent Imaging, Department of Radiology and Biomedical Imaging, University of California, San Francisco, CA, USA
[2] Department of Bioengineering, University of California, Berkeley, CA, USA
[3] Department of Bioengineering and Therapeutic Sciences, University of California, San Francisco, CA, USA
[4] Department of Medical Sciences and Technology, Indian Institute of Technology, Madras, Chennai, India
[5] Bay Area Institute of Computation, Altos Labs, Redwood City, CA, USA

Contents

5 Supplementary Information 4
5.1 Annotated Imaging Assets 4
5.2 Prompts and automation rationale 5
5.2.1 Uniform 2D prompting 5
5.2.2 Why SAM2 was evaluated without memory 5
5.3 Preprocessing Strategy for Model Compatibility 5
5.3.1 Standardizing formats 5
5.3.2 Image preprocessing 5
5.3.3 Mask preprocessing 6
5.3.4 Optional 256×256 path 6
5.3.5 Adapting data for object detection 6
5.4 Baseline Model Performance and Statistical Analysis 6
5.5 Fine-tuning scenarios (extended detail) 6
5.6 Preprocessing and Hierarchical Mixed-effects Modeling of Imaging Parameters and Fine-tuning Strategy 7
5.6.1 Data Preprocessing for Mixed-Effects Modeling 7
5.6.2 Feature Selection Using Variance Inflation Factor (VIF) 7
5.6.3 Hierarchical Mixed-Effects Modeling of MRI Parameters and Fine-tuning Strategy 7
5.7 Extended Details for Analysis of Clinical Biomarkers as a Measure of Segmentation Fidelity 7
5.7.1 Cartilage Thickness Evaluation 8
5.7.2 $T_{1\rho}$ and T_2 Relaxation Time Mapping 8
5.7.3 Intervertebral Disc Height Measurement 8
5.7.4 Muscle and Tissue Volume Analysis 8
5.7.5 Intraclass Correlation Coefficient (ICC) Analysis 8
5.7.6 Bland-Altman Analysis 9
5.7.7 Regression Analysis for Biomarker Agreement 9
5.7.8 Spearman's Rank Correlation 9
5.8 Experimental Setup for Model and Dataset Evaluation for AutoLabel 9
5.8.1 Prediction Refinement and Evaluation Workflow 9

*Corresponding author.
Email address: gabbie.hoyer@ucsf.edu (Gabrielle Hoyer)

5.9 Automated knee MRI triage cascade (extended detail) . . . 9
5.9.1 Runtime measurement . . . 10
5.10 Longitudinal biomarker extraction and clinical outcome modeling . . . 10
5.10.1 Cohort and imaging schedule . . . 10
5.10.2 Prediction tasks and feature windows . . . 10
5.10.3 Model fitting and calibration . . . 11
5.10.4 Performance metrics . . . 11
5.10.5 Sensitivity analyses . . . 11
5.10.6 Model explanation and direction-of-effect . . . 11

6 Engineering Framework and Experiment Tracking **11**
6.1 Modular Pipeline for Baseline, Fine-tuning, and Evaluation . . . 11
6.1.1 Configuration and Experiment Initialization . . . 11
6.1.2 Metadata Integration and Imaging Fidelity . . . 11
6.1.3 Structured Experiment Tracking and Logging . . . 12
6.1.4 Model Checkpointing and Reuse . . . 12
6.2 Segmentation Evaluation Pipeline . . . 12
6.2.1 Multi-Class and Multi-Instance Compatibility . . . 12
6.2.2 Evaluation Without Post-Processing . . . 12
6.3 Biomarker Quantification . . . 12
6.3.1 Metric Extraction from Segmentations . . . 12
6.3.2 Scalable, Structured Output . . . 12
6.4 Object Detection and Prompt Automation . . . 13
6.4.1 Detection Model Training for Prompt Generation . . . 13
6.4.2 Evaluation of Prompt-Based Inference . . . 13
6.5 AutoLabel System for Full Pipeline Automation . . . 13
6.5.1 Workflow Integration and Modularity . . . 13
6.5.2 System Flexibility and Future Utility . . . 13
6.5.3 Applications in Scalable Medical Imaging . . . 13

7 Results **14**
7.1 Detailed analysis of MRI acquisition parameters . . . 14
7.1.1 Effect estimates . . . 14
7.1.2 Interpretation . . . 14
7.1.3 Model checks and preprocessing . . . 14
7.2 Triage performance . . . 14
7.2.1 Demographic profile and cascade performance . . . 14
7.2.2 Stage-wise discrimination . . . 14
7.2.3 Workload and service-line implications . . . 14
7.3 OAI longitudinal biomarker extraction and clinical outcome . . . 15
7.3.1 Total-knee-replacement prediction . . . 15
7.3.2 Incident radiographic osteoarthritis prediction . . . 15

8 Figures **16**
8.1 Fine-tuning Experiments and Evaluation . . . 16
8.2 Biomarker Metrics and Evaluation . . . 20
8.3 Autolabel Pipeline Evaluation . . . 26
8.4 Downstream Pipelines . . . 31

9 Tables **34**
9.1 MRI Dataset Overview and Imaging Specifications . . . 34
9.2 Baseline Model Evaluation . . . 37
9.3 Fine-tuning Experiments and Evaluation . . . 40
9.4 Mixed Modeling Data Preparation, Quality Checking, and Results . . . 44
9.5 Biomarker Metrics and Evaluation . . . 47
9.6 Autolabel Pipeline Evaluation . . . 51
9.7 Clinical Utility Validation . . . 52

10 Data Index for external repository **60**

11 References **61**

Conventions
Section numbering in this Supplementary Materials document continues from the main manuscript to facilitate cross-referencing for readers.
Sections correspond to citations in the main text (e.g., "Supplementary Information 5.12").
Figures and tables are labeled S1, S2, . . . ; captions begin "Fig. S#" or "Table S#."
External repository items are labeled D1, D2, . . . and are listed in the Data index.

5. Supplementary Information

5.1. Annotated Imaging Assets

This study amassed diverse MRI datasets spanning various tissue types, imaging sequences, and acquisition parameters. These datasets were initially collected to investigate topics such as musculoskeletal degeneration, accelerated MRI reconstruction, and patient risk assessment;they now form a valuable basis for validating methods that can streamline clinical workflows and support patient care. In addition to advancing machine learning infrastructure and evaluation frameworks, these assets promote the development of tools for diagnosing and monitoring conditions more effectively. Dataset labels, naming conventions, demographics, and imaging protocols are summarized in Tables S0–S2.

Knee MRI datasets:

- *Knee_3D_CUBE_Research_Anatomical_300*[24]: High-resolution 3D fast spin echo (FSE) CUBE sequence with bone and cartilage labels. Acquired initially for evaluating musculoskeletal structure and informing deep learning methods for segmentation and downstream post-processing.
- *Knee_3D_undersampled_CUBE_Research_Anatomical_50*[24]: 8x-undersampled, multi-channel k-space variant of the above sequence. Enables analysis of segmentation robustness to accelerated acquisition and supports development of reconstruction-aware pipelines.
- *Knee_2D_MAPSS-echo1_Research_Compositional_39*[25] and *_22*[25]: Multi-echo MAPSS sequences used for compositional $T_{1\rho}$ and T_2 imaging. Label cartilage compartments and menisci, supporting analysis of biochemical cartilage degeneration in osteoarthritis.
- *Knee_3D_DESS_Research_Anatomical_86*: Derived from the Osteoarthritis Initiative (OAI)[26] and labeled by Stryker imorphics[27]. Used extensively for studying progressive structural pathology in articular cartilage and meniscus degeneration.

Spine MRI datasets:

- *Spine_2D_T1ax_Clinical_Anatomical_59*[28]: T_1-weighted axial sequence labeled for paraspinal musculature. Originally acquired to study sarcopenia, muscle atrophy, and fatty infiltration.
- *Spine_2D_T2ax_Clinical_Anatomical_38*[28]: T_2-weighted axial sequence with bone and disc labels. Provides high contrast for assessing lumbar disc herniation and spinal stenosis[60].
- *Spine_2D_T1sag_Clinical_Anatomical_111*[28] and *_88*[28]: T_1-weighted sagittal sequences annotated for vertebral bodies and intervertebral discs. The 88-series includes detailed vertebral and disc labels from T12 to S1, supporting studies of lumbar alignment and degenerative change.

Other anatomical sites:

- *Hip_3D_CUBE_Research_Anatomical_42*[29]: Coronal 3D CUBE sequence optimized for visualization of femoral structure and surrounding tissues. Enables investigation of geometric and structural markers associated with hip osteoarthritis[61].
- *Shoulder_3D_CUBE_Research_Anatomical_28*[30]: 3D CUBE sequence capturing high-resolution images of the scapula and shoulder joint. Supports orthopedic analysis and surgical planning[30, 31], including biomechanical modeling of joint dynamics.
- *Thigh_2D_T1ax_Clinical_Anatomical_50*[26]: T_1-weighted axial sequence labeled for muscle, fat, and bone. Applied in studies of muscular composition, fat infiltration, and systemic metabolic health[62–64].

5.2. Prompts and automation rationale

5.2.1. Uniform 2D prompting

We standardized on slice-wise bounding-box prompts for all experiments to keep comparisons model-agnostic and to support high-throughput inference across anatomies and scanners. Consistent 2D prompting avoids assumptions about temporal/spatial tracking and makes error analysis comparable across models.

5.2.2. Why SAM2 was evaluated without memory

SAM2's memory module is optimized for short videos; long, slice-rich MR volumes introduce label occlusion or entry/exit that demands repeated re-prompting, which undermines automation. Across-patient variability (e.g., body habitus, age) further degrades long-range tracking. Disabling memory yields deterministic, slice-independent prompts that scale reliably in cohort runs. Additional pilot notes are retained here to complement Methods "Prompting strategy (2D prompts)".

5.3. Preprocessing Strategy for Model Compatibility

We describe implementation details not included in the main Methods to ensure reproducibility across data sources and toolchains.

5.3.1. Standardizing formats

Inputs arrived as DICOM, NIfTI, NPZ, HDF5, or MAT with masks stored per-label or as multi-class volumes. We converted all images/masks to NIfTI, harmonized dimensionality (2D→3D stacks as needed), and consolidated per-label masks into multi-class volumes. Orientation and slice order were corrected using DICOM metadata (manual checks when incomplete), using nibabel, SimpleITK, and pydicom.

5.3.2. Image preprocessing

For each 3D volume we clipped intensities to the 1st–99th percentile of non-zero voxels, normalized to 0–255, and (for the low-footprint path) stored as 8-bit. Slices were resized to 1,024×1,024 (cubic), scaled to [0,1], RGB-stacked, and saved as NumPy .npy for fast I/O.

5.3.3. *Mask preprocessing*

We excluded labels not used in analyses and removed small objects (<100 px in 2D slices; <1,000 voxels in 3D). Masks were resized to 1,024×1,024 with nearest-neighbor interpolation, cleaned with one morphological closing pass, and saved as single-channel .npy arrays.

5.3.4. *Optional 256×256 path*

SAM/SAM2 produce 256×256 logits; keeping ground-truth masks at 256×256 during fine-tuning reduces memory and avoids upscaling in the loss. Bounding boxes are generated at 256×256 and scaled to 1,024×1,024 for inference. This path trades minor boundary sharpness for faster experiments and lower storage.

5.3.5. *Adapting data for object detection*

YOLOv8 labels were generated from masks with normalized box coordinates and saved as per-image .txt files co-located with the .npy images, enabling the integrated AutoLabel workflow.

Implementation note. All steps above are scripted and configurable (voxel thresholds, target sizes, I/O formats) to accommodate site-specific constraints. See Methods and Supplementary Data for file-level manifests.

5.4. Baseline Model Performance and Statistical Analysis

Using the unified evaluation pipeline, we assessed zero-shot performance of SAM, MedSAM, and SAM2 across MSK MRI datasets. Dice was computed per label and aggregated at the subject level; model differences were tested with a Friedman test and Wilcoxon signed-rank pairwise comparisons with Benjamini-Hochberg FDR control (5%). Summary statistics are in Supplementary Tables S3–S5. Fig. 3a,b summarizes score distributions and variance patterns, and representative overlays appear in Fig. 3c. The main Methods provide the testing framework; this section retains dataset-level results and tables for transparency.

5.5. Fine-tuning scenarios (extended detail)

This note complements the main Methods by clarifying the scope of the ablation grid and directing to extended outputs. The full configuration—scenarios, dataset mixing, data-efficiency tiers, prompt augmentation, and optimization—appears in the Methods (“Fine-tuning configuration and training setup”) and its cited tables/figures.

Scope of ablations. The complete ablation grid (encoder frozen vs. unfrozen; with/without prompt shift; single vs. mixed datasets; data-efficiency tiers) was executed on SAM to map performance-compute trade-offs comprehensively. SAM2 and MedSAM were fine-tuned only in the pooled run due to computational constraints and architectural similarity to SAM, so we use the SAM grid as the reference lens for deployment choices. Extended, tissue- and dataset-stratified outcomes are listed in Supplementary Tables S7–S9 with distributions in Supplementary Figs. S1–S4; slice-/subject-level numerics are in Supplementary Data D1–D26. The experiment index is given in Supplementary Table S6.

Initialization study. To isolate weight-initialization effects, we compared SAM, MedSAM, and SAM2 initializations under matched training configurations (both full and partial fine-tuning) using the pooled mskSAM setting across knee, spine, shoulder, and thigh. Summary outcomes are provided in Supplementary Table S9; class-level metrics appear in Supplementary Data D1–D24.

Reading the ablations. Where relevant, the Results highlight that encoder-decoder updates provided the most uniform gains across anatomies, with prompt-shift effects reported alongside strategy and dataset

factors. Readers can use Supplementary Tables S7–S9 and Figs. S1–S4 to trace these effects by tissue, sequence, and subject-set size.

5.6. Preprocessing and Hierarchical Mixed-effects Modeling of Imaging Parameters and Fine-tuning Strategy

We evaluated how MRI acquisition parameters and experimental factors relate to segmentation quality using hierarchical linear mixed-effects models with subject-level mean Dice as the outcome. The goal is exploratory: to identify protocol and configuration factors that co-vary with performance across heterogeneous anatomies and acquisitions, not to infer causal effects for any single parameter. This section documents preprocessing, multicollinearity control, and model specification; coefficients and diagnostics remain in the Supplementary tables referenced below.

5.6.1. Data Preprocessing for Mixed-Effects Modeling

Inputs included MRI metadata (TE, TR, flip angle, field strength, vendor, slice thickness, pixel spacing, image row size, SAR) and experimental covariates (acquisition mode, encoder fine-tuning, prompt shift). Missing values were imputed via iterative random-forest models (regressor/classifier) using scikit-learn[50]; continuous variables were standardized, nominal features one-hot encoded (drop-first), and ordinal features ordinally encoded. Imputation fidelity was screened with Kolmogorov-Smirnov tests; results are reported in Table S10. The preprocessed matrix was then used for multicollinearity checks.

5.6.2. Feature Selection Using Variance Inflation Factor (VIF)

We removed highly collinear predictors using variance inflation factors (threshold > 10), then recomputed VIFs to confirm reduction. The retained set is listed in Table S11. Final imputation/encoding/scaling were reapplied to the filtered matrix (see Table S11) before model fitting.

5.6.3. Hierarchical Mixed-Effects Modeling of MRI Parameters and Fine-tuning Strategy

We fit separate hierarchical models for each fine-tuning regime (Type 1: single-dataset; Type 2: grouped by anatomy/sequence; Type 3: pooled mskSAM). Dataset identity was a random intercept; fixed effects were TE, pixel spacing, slice thickness, flip angle, acquisition mode (2D=0, 3D=1), encoder fine-tuned (0/1), and bounding-box shift (0/1). Prespecified two-way interactions tested whether imaging-effect relationships differed by regime. Models were estimated by restricted maximum likelihood; 95% CIs and p-values used Wald approximations. Complete outputs are in Table S12 with visualization in Fig. 4a. Supplementary Data D25–D26 provide per-scenario design matrices and predictions.

Interpretation.. Because anatomy, label sets, and task difficulty differ across datasets, parameter effects can be partially confounded with task mix. The random-intercept structure mitigates clustering, but does not fully remove this possibility. We, therefore, regard p-values as descriptive and focus on effect sizes with 95% CIs; the results are intended to inform practical protocol guardrails and model configurations rather than prescriptive changes to scanner parameters.

5.7. Extended Details for Analysis of Clinical Biomarkers as a Measure of Segmentation Fidelity

We compared quantitative imaging biomarkers derived from expert annotations with those computed from model-predicted masks across knee, spine, hip, shoulder, and thigh datasets to assess measurement fidelity. Biomarkers included cartilage thickness[65–67], $T_{1\rho}/T_2$[68–70], intervertebral disc height[28, 71], and muscle/bone volumes[62–64, 72–74]. All measurements were scaled to physical units using DICOM metadata (PixelSpacing, SliceThickness). Dataset- and anatomy-level summaries are reported in Supplementary Data D27–D52.

Terminology and measurement classes We use *biomarker* for any quantitative MRI measurement computed from a segmentation mask. Established markers here are cartilage thickness (medial-axis + Euclidean distance transform), intervertebral disc height (cranio-caudal extent), $T_{1\rho}/T_2$ relaxation times (masked averages), and muscle volume (voxel volumetry). *Proxy* measures (meniscus thickness; bone volume) extend tissue coverage and stress-test automation but are not positioned as disease endpoints in this study. Extraction specifics follow below; the main Methods summarize definitions and agreement statistics.

Abbreviations: ICC, intraclass correlation coefficient; LoA, limits of agreement; SE, standard error; SD, standard deviation.

5.7.1. *Cartilage Thickness Evaluation*

Cartilage thickness was computed by applying a medial-axis transform to each cartilage mask and sampling the Euclidean distance to the nearest boundary along the medial-axis skeleton (the centerline equidistant from the mask boundaries); values were averaged per anatomical compartment and scaled to mm via voxel dimensions[65, 75]. Subject-level summaries aggregate across slices and compartments (Supplementary Fig. S6).

Cartilage thickness was computed by applying a medial-axis transform to each cartilage mask and sampling the Euclidean distance to the nearest boundary along the medial-axis skeleton (the centerline equidistant from the mask boundaries); values were averaged per anatomical compartment and scaled to physical units (Supplementary Fig. S6a).

5.7.2. *$T_{1\rho}$ and T_2 Relaxation Time Mapping*

Cartilage masks were applied to parametric $T_{1\rho}$ or T_2 maps; voxel values were clipped to 0–100 ms and averaged within each cartilage compartment (medial/lateral femoral, medial/lateral tibial, and patellar when present), then aggregated to subject level[68–70]. These steps were performed for both manual and model masks to enable paired agreement analyses Fig. S7 & Fig. S9).

5.7.3. *Intervertebral Disc Height Measurement*

Disc instances were identified by connected components. For each slice, we derived the minimal bounding rectangle of the disc contour and used its cranio-caudal extent as slice-level height; subject-level disc height per level was the maximum across slices (mm via DICOM spacing)[28, 71] (Supplementary Fig. S10).

5.7.4. *Muscle and Tissue Volume Analysis*

For each tissue mask and slice, we multiplied voxel counts by the voxel volume $v = \text{PixelSpacing}_x \times \text{PixelSpacing}_y \times \text{SliceThickness}\,[\text{mm}^3]$, summed across slices, and reported volumes in cm^3 (divide by 1,000). This was applied to thigh and lumbar muscle groups and to knee/hip bones, using both manual and model masks (Fig. S5 & Fig. S8).

5.7.5. *Intraclass Correlation Coefficient (ICC) Analysis*

We screened distributional assumptions with Shapiro-Wilk and Levene (Supplementary Table S13). When assumptions held, we computed absolute-agreement, two-way mixed-effects single-measurement ICC (ICC(3,1)) with 95% CIs (Pingouin)[76]; otherwise we estimated ICC from a linear mixed model (subject random intercept) and obtained 95% CIs by bootstrap (10,000 resamples). Per-biomarker outputs are summarized in Supplementary Table S14 and visualized in Fig. 4b with dataset-specific views in S5–S10.

5.7.6. Bland-Altman Analysis

Parametric Bland-Altman (mean bias; limits of agreement = mean ± 1.96 SD) was used when normality/variance assumptions held; otherwise a percentile-based non-parametric version reported median bias and 2.5th–97.5th percentile limits. Uncertainty was quantified by 10,000 bootstrap resamples. Plots appear in Fig. 4b and Supplementary Figs. S5–S10; assumption checks in Supplementary Table S13.

5.7.7. Regression Analysis for Biomarker Agreement

For parametric cases we fit ordinary least squares (manual = outcome, automated = predictor) and report intercept, slope, R, R^2, p (Supplementary Table S15). When assumptions were violated, we used Gaussian Process Regression with kernel $K(x, x') = \mathrm{RBF}(x, x') + \mathrm{WhiteKernel}$ (scikit-learn), reporting mean prediction and 95% pointwise intervals (Supplementary Figs. S5–S10; Table S15).

5.7.8. Spearman's Rank Correlation

To complement ICC and Bland-Altman when distributions were non-normal or monotone-only, we computed subject-level Spearman's ρ with two-sided p values (SciPy) (Supplementary Table S16).

5.8. Experimental Setup for Model and Dataset Evaluation for AutoLabel

This section expands the main Methods by detailing post-processing kernel choices, unit-of-analysis, and visualization conventions used to evaluate detector-prompted segmentation across representative MSK datasets and both zero-shot and best fine-tuned settings (with/without bounding-box shift).

5.8.1. Prediction Refinement and Evaluation Workflow

Predicted logits were sigmoid-thresholded at 0.5, small components removed (connected components), and a single morphological closing pass applied. Kernel sizes were dataset/label-specific: 7×7 for finer structures and up to 15×15 for coarse labels; a matching Gaussian blur smoothed boundaries. Slice-wise Dice was aggregated to subject level and then to dataset level (Supplementary Data D53–D54).

For statistical comparisons, subject-level Dice under GT vs. YOLO prompts were contrasted with two-sided pairwise Wilcoxon rank-sum tests with Benjamini-Hochberg FDR control (5%) (Table S17). Distributions are shown as raincloud plots (Fig. 5a).

Figs. S11–S15 show five-panel exemplars (YOLO box overlay, GT overlay, prediction overlay, GT contour, prediction contour) for three subjects per dataset, illustrating the impact of detector-prompt quality on mask fidelity.

5.9. Automated knee MRI triage cascade (extended detail)

Demographics, class balance, and routed cohort composition are summarized in Table S19. Stage-wise operating points, confusion matrices, and sensitivity/specificity summaries appear in Tables S18–S19. Probability calibration for the six Stage-C tasks is shown in Fig. S16 (10 equal-width bins). Threshold-workload trade-offs (forwarded fraction vs. verification time) are in Fig. S16b.

Throughput: raw per-scan timing CSVs and detection/segmentation/biomarker breakdowns per GPU are provided as Supplementary Data; summary medians/IQRs are reported in the Results.

5.9.1. *Runtime measurement*

We timed AutoLabel on 30 randomly sampled knee 3D CUBE scans after a two scan warm up. "Model compute" includes the detector, SAM2 segmentation, and per slice biomarker updates. File I/O and end run aggregation were timed separately and excluded from the model compute figure.

On a TITAN RTX the medians were: model compute 42.6 s per scan [detection 8.3 s; segmentation 21.9 s; biomarker updates 12.4 s]. The wall clock without I/O was 103.3 s (IQR 99.0 to 111.4), and file load was 11.2 s.

On an A100 40 GB the medians were: model compute 30.1 s [5.9 s; 12.5 s; 11.7 s]. The wall clock without I/O was 249.9 s (IQR 232.2 to 276.1), and file load was 7.3 s.

The gap between model compute and wall clock reflects preprocessing and bookkeeping in the research script used for cohort runs. The pipeline was not tuned for per scan latency; the goal was cohort scale extraction of biomarkers for triage and longitudinal modeling. Raw per scan timings (CSV) are stored with the run outputs.

Workload estimation. Triage workload was estimated as *forwarded_fraction* $\times$ *per exam minutes* and reported per 1,000 scans. For example, $99/930 \times 1{,}000 \times 2$ min $= 212.9$ min $= 3.5$ h at the 85% setting, and $47/930 \times 1{,}000 \times 2$ min $= 101.1$ min $= 1.7$ h at the 90% setting.

5.10. Longitudinal biomarker extraction and clinical outcome modeling

This section provides extended cohort definitions, modeling settings, and sensitivity outputs that complement the main Methods.

5.10.1. *Cohort and imaging schedule*

Sagittal 3D DESS scans from the Osteoarthritis Initiative were quality-checked, leaving 1,109 index knees (baseline demographics in Table S20). Imaging at 0, 12, 24, 36, and 48 months; surgery status tracked to 96–120 months.

Automated biomarker assembly. At each visit AutoLabel (mskSAM2) generated cartilage and meniscus masks. Mean thickness and annual change were z-scaled to a Kellgren-Lawrence 0–1 reference. Age, sex, and BMI were forward-filled when missing (<3%).

5.10.2. *Prediction tasks and feature windows*

1. Total knee replacement (TKR): Horizons 48–96 months (main) and 48–120 months (sensitivity). Candidate learners were screened under a 48-month landmark; a 400-tree random forest (min_samples_leaf=10) outperformed alternatives and was selected, with a logistic-regression baseline.
2. Incident radiographic osteoarthritis (OA): The subset with baseline KL < 2 ($n = 743$) formed the OA-incidence cohort. L2-regularized logistic regression was carried forward for 48–96m and 48–120m horizons.

Preliminary screens also compared L1-penalized logistic regression, random forest, XGBoost, soft voting, and stacking; this favored logistic regression for OA and random forest for TKR. Demographics-only baselines (age, sex, BMI at 48m) are listed in Tables S20 and S22.

5.10.3. *Model fitting and calibration*

Random forest and logistic regression used all data up to the 48-month landmark. Logistic regression: median imputation, standardization, L2 penalty (solver=lbfgs, max_iter=1,000), class_weight="balanced". Random forest: median imputation, 400 trees, min_samples_leaf=10, class_weight="balanced". Five-fold `StratifiedGroupKFold` (group=participant) preserved paired knees. Out-of-fold probabilities were isotonic-calibrated; discrimination and decision curves used calibrated probabilities. We report Brier error for raw vs. calibrated probabilities. AUC CIs used percentile bootstrap (1,000 draws); decision-curve bands used bootstrap (2,000 draws). Cox comparators used month-0 features (Harrell C; IPCW C)[55, 56]; 95% intervals were mean $\pm$ 1.96$\times$(SD/$\sqrt{5}$) across folds (Tables S20–S25).

5.10.4. *Performance metrics*

We report AUC (bootstrap 95% CI), calibration slope, Brier (raw, calibrated), and decision-curve net benefit[22] under three penalty settings (standard; MRI 0.20; surgery 1.00). Complete tables: S20–S23. ROC, Kaplan-Meier, and decision curves: Fig. S17.

5.10.5. *Sensitivity analyses*

Sensitivity analyses included per-scan mean imputation for missing visits (Tables S24–S25) and an extended 48–120m TKR horizon to assess long-term discrimination drift.

5.10.6. *Model explanation and direction-of-effect*

TreeSHAP was used for random forests and SHAP's linear explainer for logistic regression (standardized inputs). Mean absolute SHAP ranked features at the 48-month landmark; direction-of-effect plots pair SHAP with feature values at each visit (Supplementary Fig. S18).

6. Engineering Framework and Experiment Tracking

We built an integrated engineering framework to make the study reproducible and clinically relevant. It supports baseline testing, fine-tuning, prompt-source experiments, and downstream biomarker quantification through standardized metadata handling, YAML-based workflows; additionally, there is optional object-detection integration and automated evaluation.

6.1. Modular Pipeline for Baseline, Fine-tuning, and Evaluation

6.1.1. *Configuration and Experiment Initialization*

We used YAML configuration files to declare dataset composition, image resolution, and preprocessing; model/training knobs (optimizer/scheduler/batch/early-stopping) are recorded in the configs rather than restated here. Hardware parameters (single-/multi-GPU, gradient accumulation, learning-rate scaling) load at runtime to keep runs reproducible across compute budgets.

6.1.2. *Metadata Integration and Imaging Fidelity*

To maintain anatomical accuracy and clinical relevance, we integrated metadata at both the slice and subject levels. Slice-level metadata included spatial properties extracted from DICOM headers, such as pixel spacing, slice thickness, and instance identifiers. Subject-level metadata captured demographics and imaging parameters, including scanner field strength, acquisition type, age, and sex.

These metadata were stored in Parquet tables and used throughout the pipeline to guide dataset parti-

tioning and to scale segmentation-derived metrics into physical units. For example, cartilage thickness and disc height were calculated in millimeters, and tissue volumes were expressed in cubic centimeters. This integration ensured that outputs remained interpretable in clinical contexts.

6.1.3. Structured Experiment Tracking and Logging

We organized runs in labeled directories with configs, logs, and outputs co-located. Weights & Biases (W&B)[77] captured training/validation losses and Dice; when unavailable, JSON logs were saved locally. QC images (mask/overlay/box) were emitted automatically to support rapid review. This structure makes configuration-level effects easy to trace and preserves reproducibility across experiments.

6.1.4. Model Checkpointing and Reuse

Checkpoints were saved automatically and, when trained with DDP, cleaned to remove prefix keys so they load on a single GPU. Each checkpoint ships with the exact config snapshot and a short metadata summary to enable reuse (fine-tune, inference, or benchmarking) without code edits.

6.2. Segmentation Evaluation Pipeline

We evaluated baseline and fine-tuned models with standard segmentation metrics (Dice, IoU) computed at slice, subject, and dataset levels. Runs are config-driven; results are logged to W&B or saved as CSV/JSON. QC overlays are produced to surface preprocessing or inference issues early.

6.2.1. Multi-Class and Multi-Instance Compatibility

To handle MSK multi-class/multi-instance cases, a custom dataset creates per-label masks and robust boxes, with a collation function that batches variable label counts. We use PyTorch[78]/MONAI[79] for inference and per-label Dice, aggregated to subject and dataset summaries for downstream statistics.

6.2.2. Evaluation Without Post-Processing

Primary segmentation evaluations are based on raw model outputs (no hole-filling or smoothing) to avoid post-hoc variability. This keeps differences attributable to model/training conditions and establishes a reproducible benchmark. (The AutoLabel experiment uses a standardized refinement recipe reported in §5.8.1, applied *only* for that detector-prompted setting.)

6.3. Biomarker Quantification

6.3.1. Metric Extraction from Segmentations

We compute MRI biomarkers, including cartilage thickness (medial-axis + Euclidean distance transform), tissue volumes (voxel counts), disc heights (cranio-caudal extent), and $T_{1\rho}/T_2$ (masked intensity means). All values are scaled to physical units using DICOM spacing and slice thickness to remain clinically interpretable across protocols.

6.3.2. Scalable, Structured Output

Biomarker outputs were saved in structured CSV files organized by subject and experiment. Each run was configured via YAML templates specifying the set of biomarkers to compute, target anatomical labels, and scale-conversion metadata. The modular design enabled consistent evaluation across datasets while allowing researchers to modify parameters as needed for different use cases. This approach supports longitudinal studies, site-to-site comparisons, and clinical applications where anatomical accuracy and metric

standardization are essential.

6.4. Object Detection and Prompt Automation

6.4.1. Detection Model Training for Prompt Generation

To automate bounding-box prompts, we trained YOLOv8[47] on five MSK datasets using simple YAML configs per dataset (classes/paths/splits). The goal was integration speed and reproducibility, not advancing detection; YOLO serves purely to generate prompts for segmentation at scale.

6.4.2. Evaluation of Prompt-Based Inference

Detector outputs are aligned to slices and passed to SAM/SAM2; predictions are batched and aggregated to volumes. Optional refinement (connected components, one closing pass, light smoothing) is available for downstream visualization tasks. This setup tests segmentation under realistic, imperfect prompts.

6.5. AutoLabel System for Full Pipeline Automation

6.5.1. Workflow Integration and Modularity

AutoLabel integrates detection, segmentation, and evaluation into a streamlined, automated workflow. The system processes raw DICOM or NIfTI volumes slice-by-slice, applying standard preprocessing steps like intensity normalization, resolution adjustment, and tensor formatting. After detection models produce bounding box prompts, segmentation is performed with SAM or SAM2. The resulting masks are assembled into full 3D volumes and saved in NIfTI format.

AutoLabel supports flexible input handling (e.g., folders of DICOMs, individual NIfTI files) and ensures spatial consistency by sorting slices and applying uniform preprocessing. These features make it suitable for use with varied datasets and MRI protocols.

6.5.2. System Flexibility and Future Utility

The pipeline was designed for extensibility. New detection or segmentation models can be incorporated by modifying configuration files without altering core logic. This modularity allows rapid testing of new architectures, supports comparative studies, and prepares the system to accommodate evolving foundation models or imaging tasks.

Because the entire inference process is defined via YAML, changes to model weights, preprocessing parameters, or post-processing settings can be applied consistently and reproducibly. The system aligns with the broader need in clinical AI for adaptable infrastructure that allows controlled evaluation of updated models as datasets grow or deployment contexts shift.

6.5.3. Applications in Scalable Medical Imaging

AutoLabel enables large-scale segmentation across MRI datasets without manual intervention. Its design supports cohort-level analyses, dataset construction, and biomarker extraction with minimal overhead. Periodic visualizations (e.g., every fifth slice) are optionally generated to assist with qualitative review and quality assurance. Outputs land in standardized directories for immediate downstream use. AutoLabel is meant to bridge model research and scalable deployment: automation where safe, clear configs, and repeatable logs. Experiments ran on multi-GPU HPC nodes; code and configs are available for reuse (https://github.com/gabbieHoyer/AutoMedLabel).

7. Results

7.1. Detailed analysis of MRI acquisition parameters

Hierarchical mixed-effects models (Supplementary Tables S10–S12; data tables D25–D26) were fit to quantify the association between imaging parameters and subject-level mean Dice scores after single-dataset training, anatomy-grouped training, and full musculoskeletal fine-tuning. All continuous variables were scaled to one standard deviation (SD) before fitting to allow direct comparison of effect sizes.

7.1.1. Effect estimates

- Flip angle was the strongest positive contributor. A one-SD increase in flip angle raised Dice by 0.71–0.77 (95% CI 0.46–1.08) depending on the training scenario.
- Pixel spacing and echo time (TE) showed the opposite pattern. Each SD increase in pixel spacing lowered Dice by up to 0.08, and each SD increase in TE lowered Dice by up to 0.10.
- A TE × slice-thickness interaction was observed in the anatomy-grouped setting, indicating that thicker slices partly offset TE-related losses.

7.1.2. Interpretation

These coefficients represent statistical associations rather than causal effects. The patterns suggest that greater contrast (via higher flip angle) and finer spatial sampling (via smaller pixel spacing) are linked to improved segmentation performance, whereas longer TE and coarser sampling may diminish boundary definition. The observed attenuation of parameter effects after full multi-dataset fine-tuning suggests that training with heterogeneous protocols can reduce the model's dependence on any single acquisition setting.

7.1.3. Model checks and preprocessing

Preprocessing steps included encoding of categorical variables, imputation of missing acquisition parameters, scaling, and variance inflation checks (Supplementary Tables S10–S11). Full fixed-effect coefficients and confidence intervals are reported in Supplementary Table S12.

7.2. Triage performance

7.2.1. Demographic profile and cascade performance

Subject characteristics for every branch of the knee-triage cascade are listed in Table S19. The full cohort (930 knees) averaged 44.8 ± 15.3 years; 52.7% were women. Sex and age distributions aligns with those of the wider musculoskeletal segmentation study, which supports direct comparison between segmentation fidelity and downstream clinical utility.

7.2.2. Stage-wise discrimination

Stage-wise operating points, confusion matrices, and calibration for all tasks are summarized in Supplementary Tables S18–S19 and Supplementary Fig. S16. Throughput timing CSVs and per-component breakdowns are provided as Supplementary Data; summary medians/IQRs are in the main Results.

7.2.3. Workload and service-line implications

Workload math and routed-cohort composition appear in Tables S18–S19 with calibration in Fig. S16; timing CSVs are in Supplementary Data. These triage outputs use the same validated biomarker layer that feeds the longitudinal risk models, tying near-term routing to long-term prediction.

7.3. OAI longitudinal biomarker extraction and clinical outcome

7.3.1. Total-knee-replacement prediction

Full 48→96-month landmark outputs (AUC, calibration, Brier, decision curves) are tabulated in Table S20 and visualized in Fig. 7 (main). *Extended horizon* (48→120 months) results are provided in Table S21; discrimination and net-benefit remain positive over relevant risk thresholds, with survival separation shown in Fig. S17.

7.3.2. Incident radiographic osteoarthritis prediction

Complete 48→96-month metrics and decision curves appear in Table S22 (main figure shows summary); *extended 48→120-month* discrimination is in Table S23. Sensitivity analyses for imputation and later landmarks are reported in S24–S25. Curves are collated in Fig. S17; SHAP exemplars in Fig. S18.

8. Figures

8.1. Fine-tuning Experiments and Evaluation

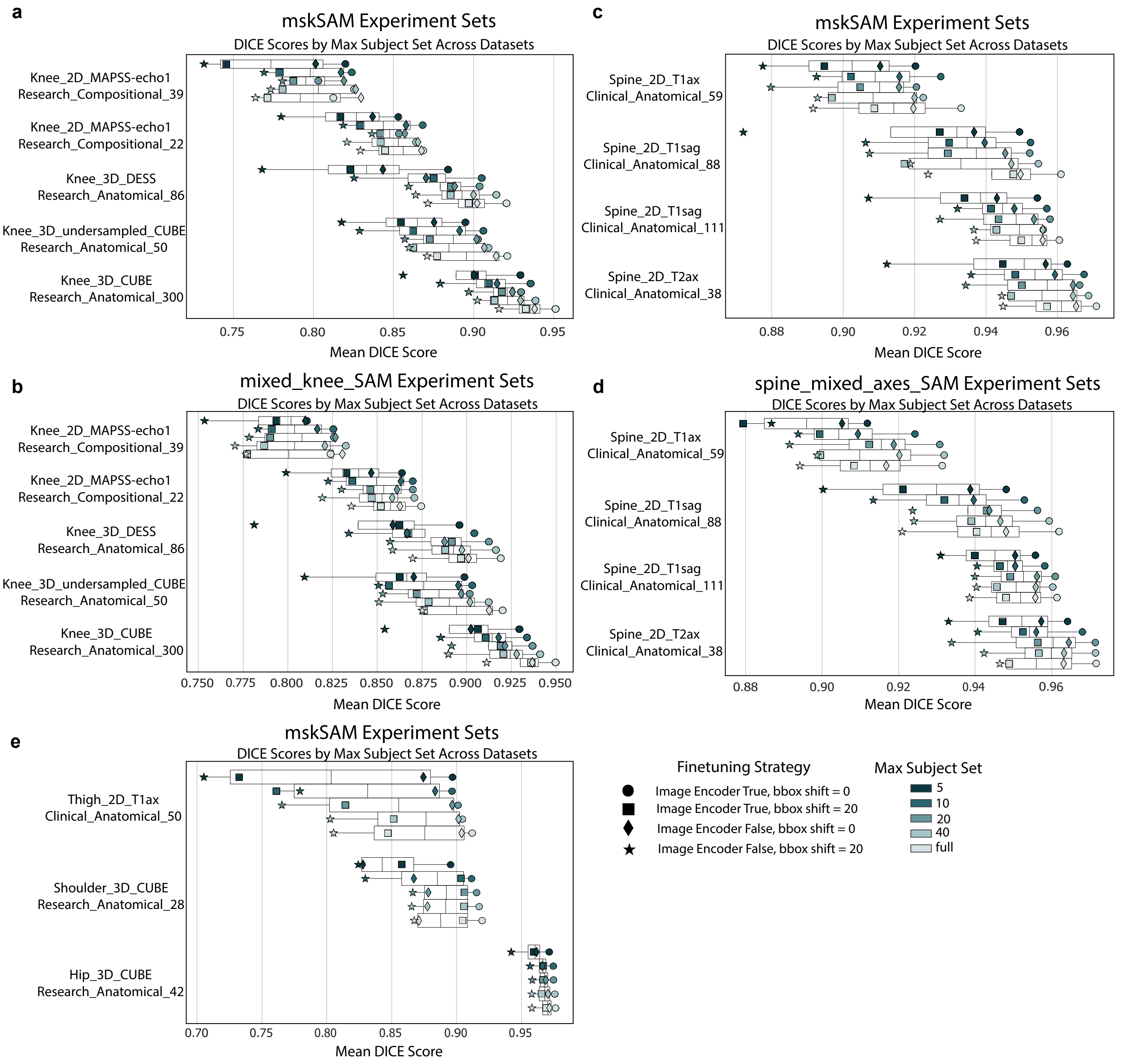

Fig. S1: Extended Evaluation of SAM Model Fine-tuning in Musculoskeletal MRI. This figure presents box plots illustrating DICE score distributions across knee, spine, shoulder, hip, and thigh datasets under various fine-tuning strategies and subject set sizes. The top row displays results from the mskSAM_SAM experiment, with separate box plots for knee, spine, and shoulder/hip/thigh datasets. Each plot shows DICE score distributions stratified by subject set size (5, 10, 20, 40, full). Colours progress from deep teal for the 5-subject set through successively lighter teal hues, reaching a pale blue-gray for the full set, so the fill shade alone indicates the cohort size. Fine-tuning strategies are represented by distinct symbols (circle, square, diamond, star), which correspond to different combinations of image encoder fine-tuning and bounding box shift. The bottom row provides results for the mixed_Knee_SAM and spine_mixed_axes_SAM experiments, with box plots for knee and spine datasets, respectively. As with the top row, DICE scores are shown for different subject set sizes and fine-tuning strategies. The box plots display the median, interquartile range (IQR), and any outliers, providing a clear visualization of the statistical distribution across test subjects.

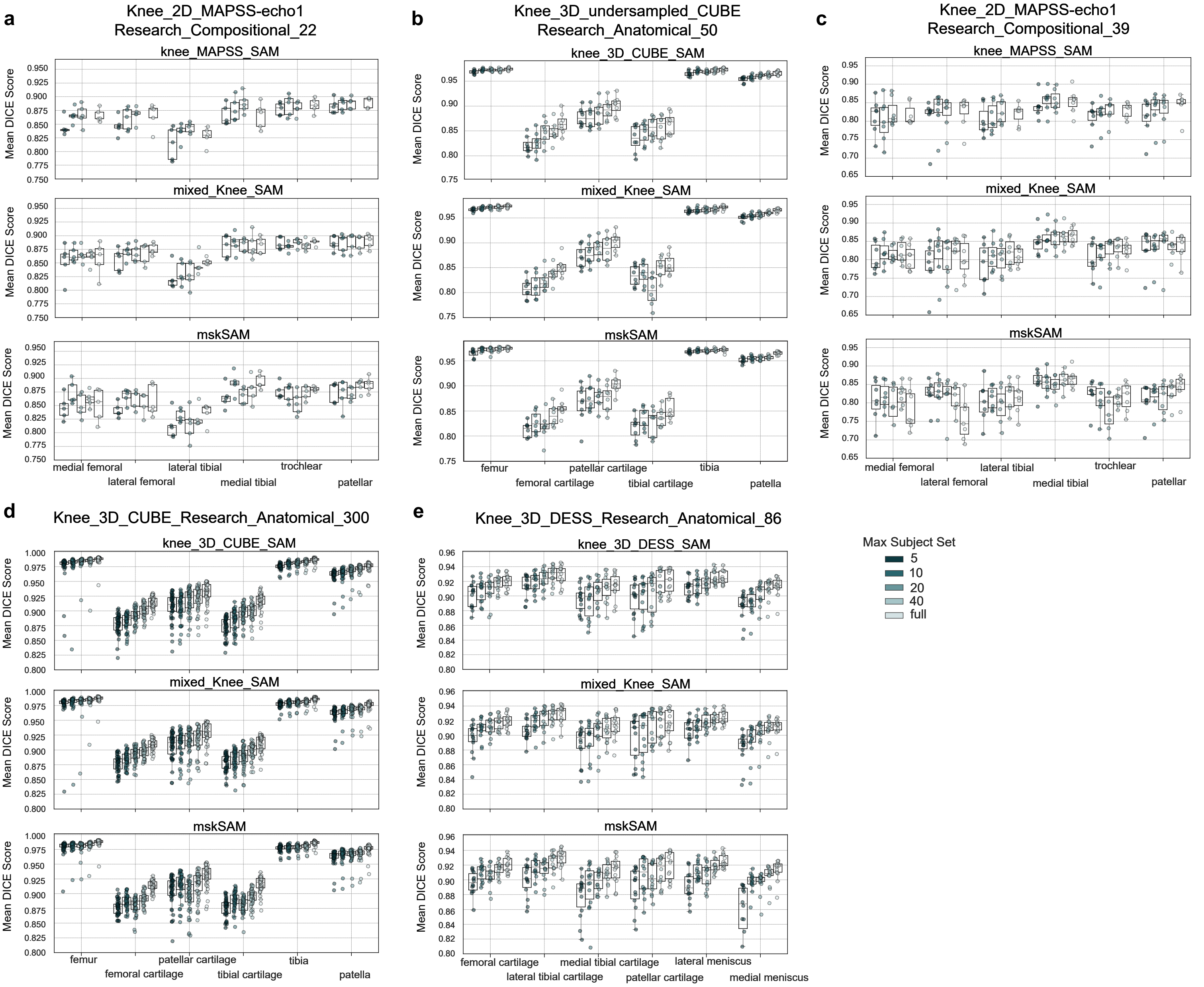

Fig. S2: Label-Specific Fine-tuning Performance for Knee Datasets.
This figure presents class-level DICE score distributions across various fine-tuning strategies for knee datasets. Box plots display performance for individual anatomical labels, comparing three fine-tuning conditions: single-dataset fine-tuning, mixed-knee fine-tuning, and comprehensive mskSAM fine-tuning. Each plot represents a specific knee dataset, with DICE scores stratified by label, including cartilage, bone, and meniscus. Coloured symbols represent the fine-tuning strategy applied, consistent with those used in Fig. 3d–f. This figure provides a detailed view of how segmentation performance varies across specific anatomical labels; this highlights the impact of each fine-tuning strategy at the label level rather than whole-dataset averages.

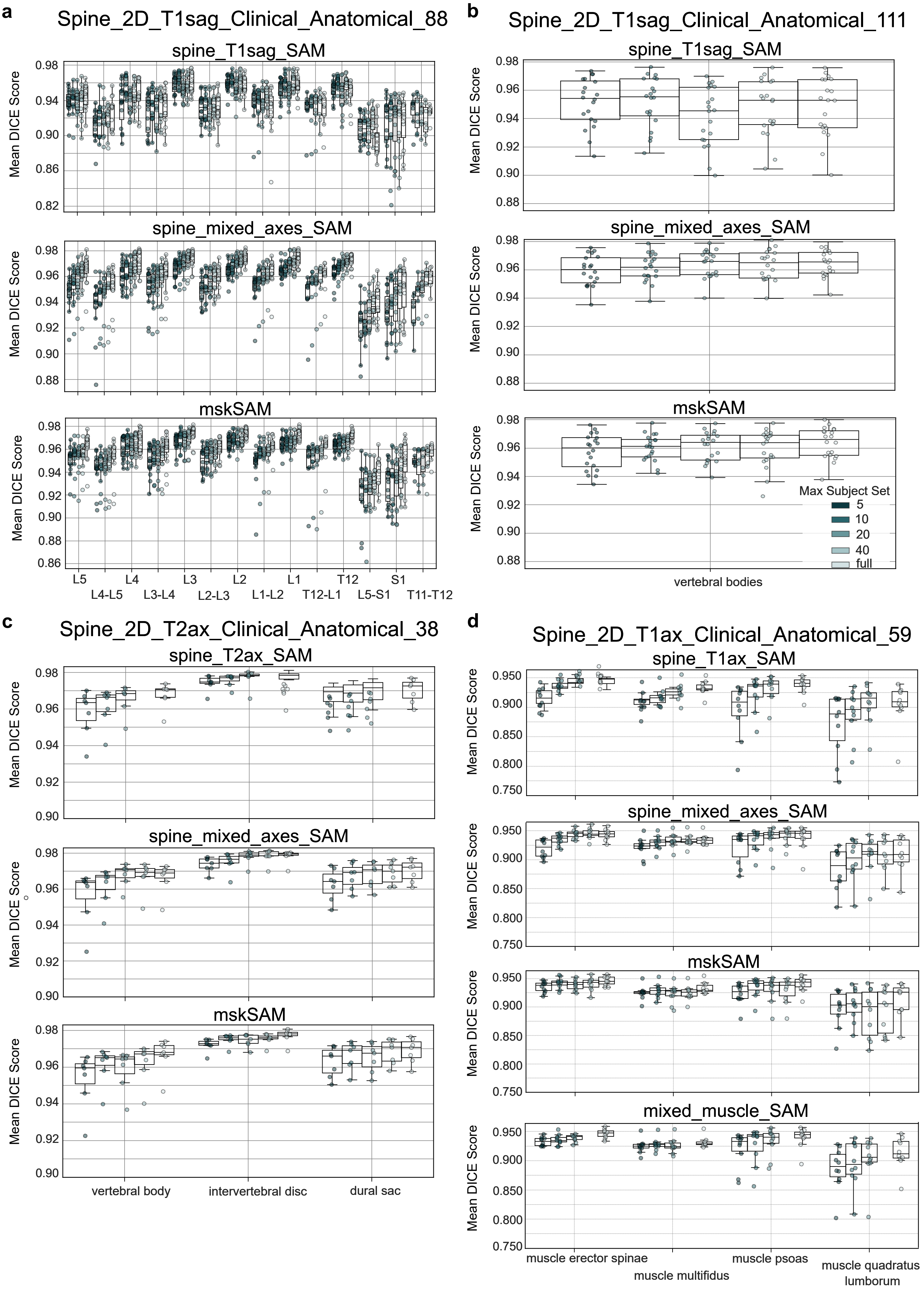

Fig. S3: Label-Specific Fine-tuning Performance for Spine Datasets.
This figure presents DICE score distributions for spine datasets, with class-level performance displayed across different fine-tuning strategies. The plots compare three fine-tuning conditions: single-dataset fine-tuning, mixed-spine fine-tuning, and comprehensive mskSAM fine-tuning; the Spine_2D_T1ax_Clinical_Anatomical_59 dataset also compares the mixed_Muscle_SAM fine-tuning condition (paired with the Thigh_2D_T1ax_Clinical_Anatomical_50 dataset). Each box plot shows DICE scores for individual anatomical labels, such as vertebral bodies and spinal discs, with symbols representing different subject set sizes (5, 10, 20, 40, full). The figure provides insights into the impact of fine-tuning strategies on segmentation performance at the label level for spine datasets. This allows for comparison between fine-tuning approaches and the consistency of segmentation across various anatomical structures.

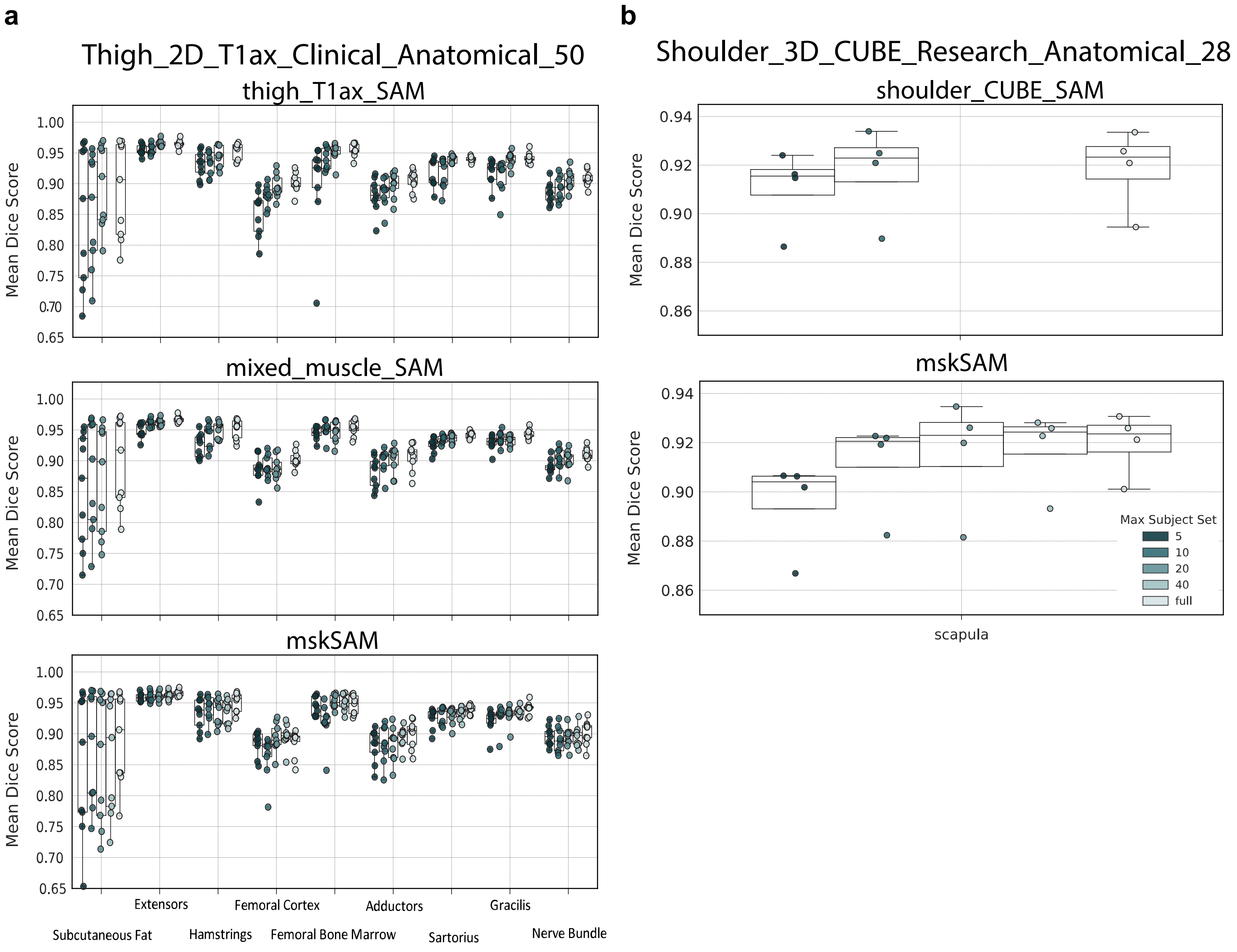

Fig. S4: Label-Specific Fine-tuning Performance for Thigh and Shoulder Datasets. This figure shows class-level DICE score distributions for thigh and shoulder datasets under different fine-tuning strategies. The top row displays results for the Thigh_2D_T1ax_Clinical_Anatomical_50 dataset across three fine-tuning conditions: thigh-only fine-tuning, mixed-muscle fine-tuning (paired with the Spine_2D_T1ax_Clinical_Anatomical_59 dataset), and comprehensive mskSAM fine-tuning. Each box plot shows performance for specific anatomical labels, including muscle and fat, with coloured symbols representing different subject set sizes (5, 10, 20, 40, full). The bottom row presents results for the Shoulder_3D_CUBE_Research_Anatomical_28 dataset under two fine-tuning conditions: shoulder-only fine-tuning and mskSAM fine-tuning. DICE scores are shown for various shoulder- specific anatomical labels, with a similar breakdown by subject set size. This figure provides insights into label-specific segmentation performance for both thigh and shoulder datasets, comparing the effects of dataset-specific, mixed-dataset, and comprehensive fine-tuning strategies.

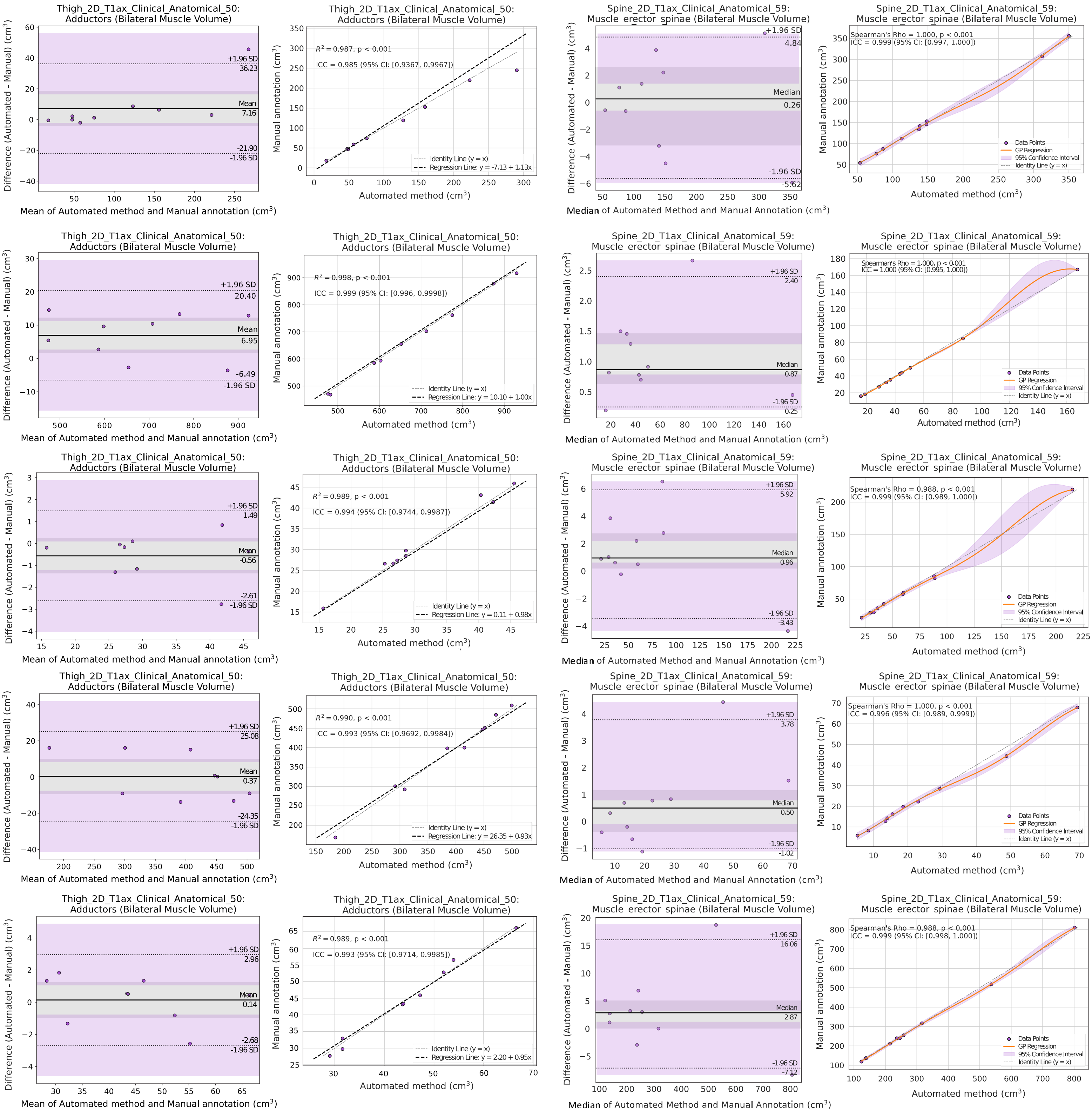

Fig. S5: Muscle Volume Agreement.
This figure presents the class-label level comparison of muscle volume measurements between manual annotations and model predictions for the Thigh_2D_T1ax_Clinical_Anatomical_50 and Spine_2D_T1ax_Clinical_Anatomical_59 datasets. Each row features Bland-Altman plots (left) showing the differences between manual and model-derived values, and regression plots (right) illustrating the correlation between methods. Linear regression is applied for normally distributed data, with R^2, p-values, and ICC3 results displayed. Non-normal datasets are evaluated using Gaussian Process regression, with Spearman's rho, p-values, and bootstrapped mixed-effects ICC results.

TOC

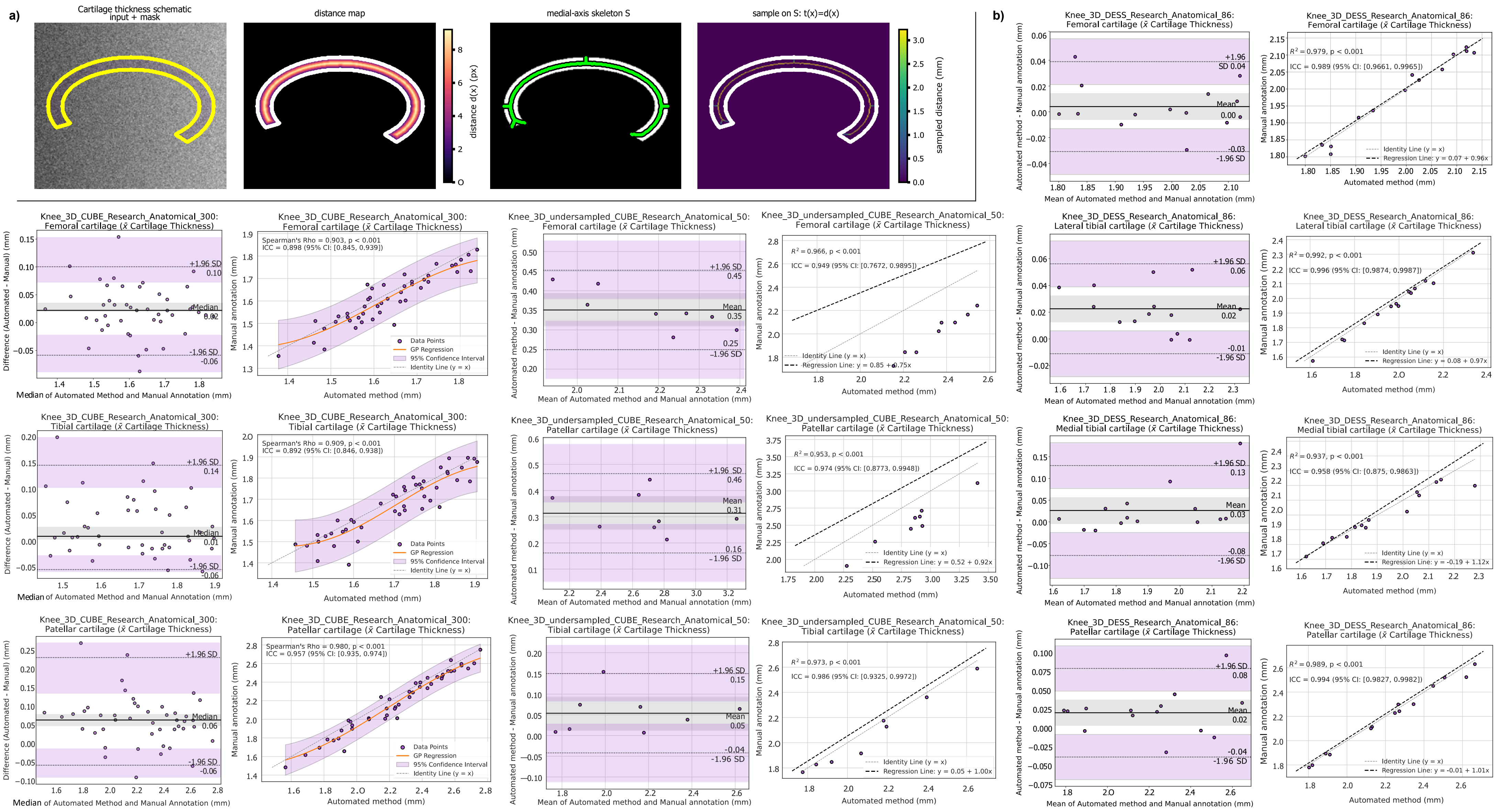

Fig. S6: Cartilage Thickness Agreement.
(a) Schematic of cartilage thickness computation from a binary cartilage mask. A Euclidean distance transform $d(x)$ is computed inside the mask; the medial-axis skeleton S is the set of interior points equidistant from opposing boundaries; thickness samples are obtained along S and scaled to physical units using pixel spacing. **(b)** The agreement at the class-label level between manual and model-predicted cartilage thickness measurements across the Knee_3D_DESS_Research_Anatomical_86 and Knee_3D_CUBE_Research_Anatomical_300 datasets. Bland-Altman plots (left) depict the measurement differences, while regression plots (right) evaluate correlations between manual and automated methods. Both normal (linear regression, R^2, p-values, ICC3) and non-normal (Gaussian Process regression, Spearman's rho, bootstrapped ICC) data evaluations are presented, illustrating concordance between the two methods.

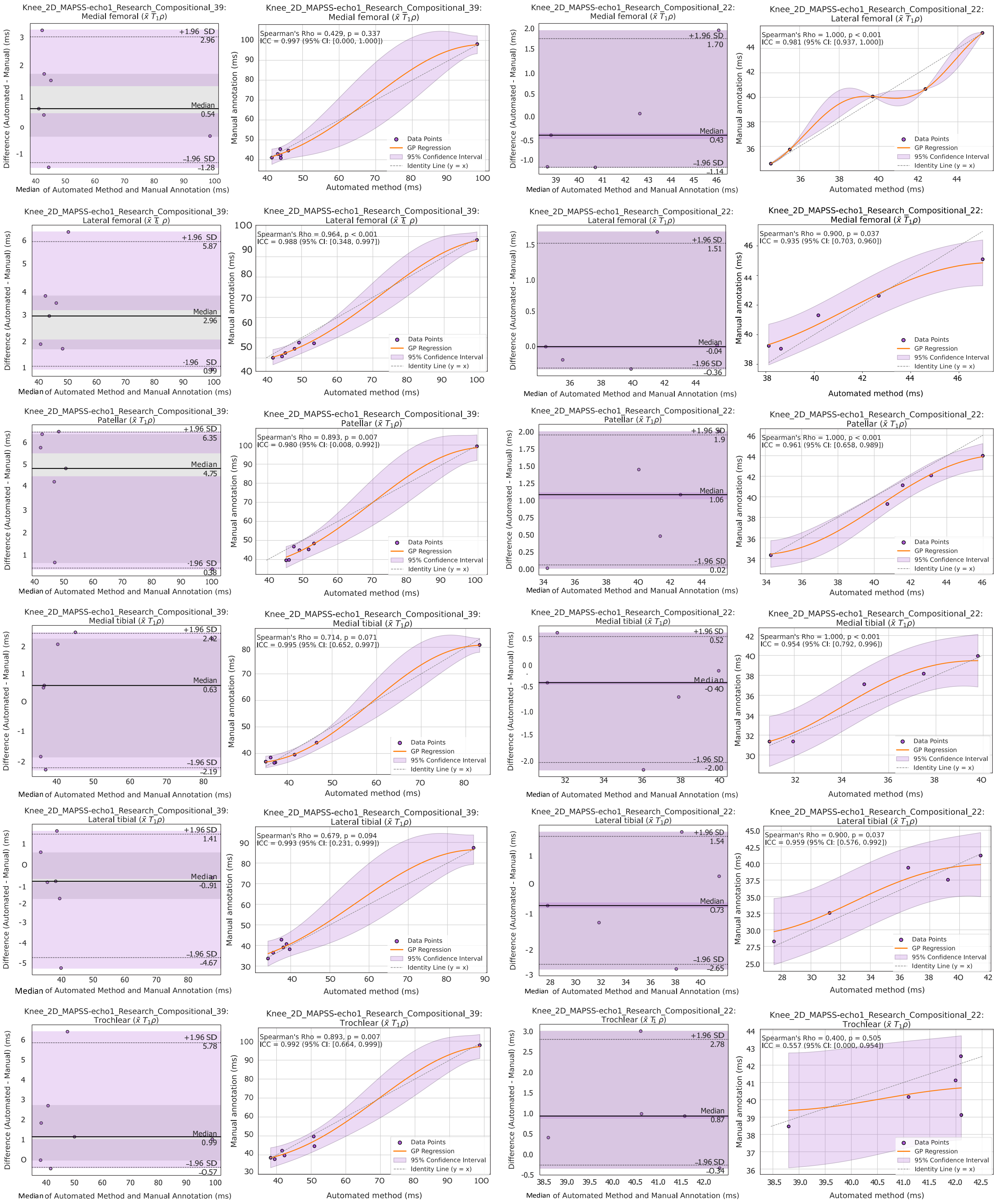

Fig. S7: $T_{1\rho}$ Agreement. This figure presents the class-label level agreement for $T_{1\rho}$ relaxation times between manual annotations and model predictions for the Knee_2D_MAPSS-echo1_Research_Compositional_22 and Knee_2D_MAPSS-echo1_Research_Compositional_39 datasets. Bland-Altman plots (left) display the differences, while Gaussian Process regression plots (right) are used for non-normal datasets. All datasets in this figure are non-normally distributed, with Spearman's rho, p-values, and bootstrapped mixed-effects ICC results assessing the concordance of manual and model- derived measurements.

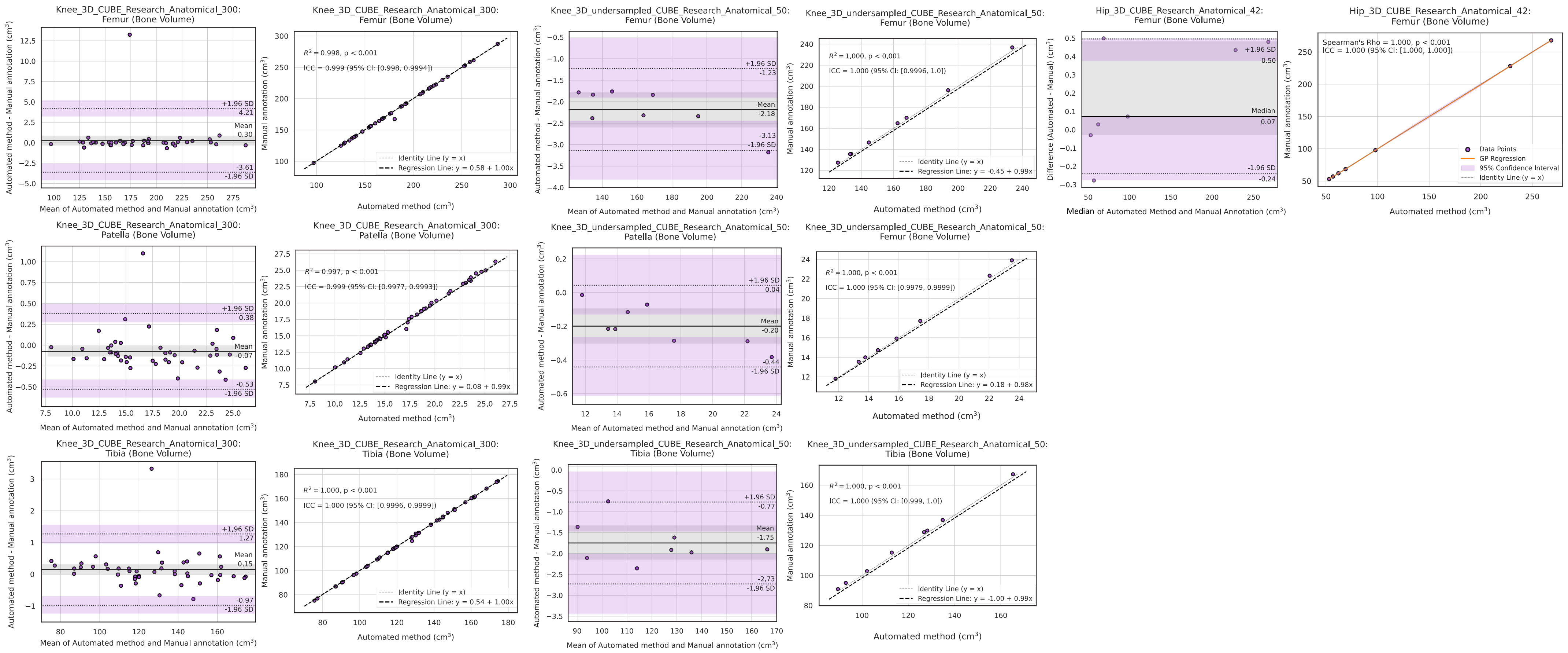

Fig. 8: Bone Volume Agreement. This figure compares bone volume measurements at the class-label level between manual and model-predicted segmentations for the Knee_3D_CUBE_Research_Anatomical_300, Knee_3D_undersampled_CUBE_Research_Anatomical_50, and Hip_3D_CUBE_Research_Anatomical_42 datasets. Bland-Altman plots (left) illustrate the differences between methods, and regression plots (right) display the statistical relationship. Linear regression is used for normal data, while Gaussian Process regression is applied to non-normal datasets, with relevant statistical metrics (R^2, p-values, ICC3 for normal, Spearman's rho and bootstrapped ICC for non-normal data) provided.

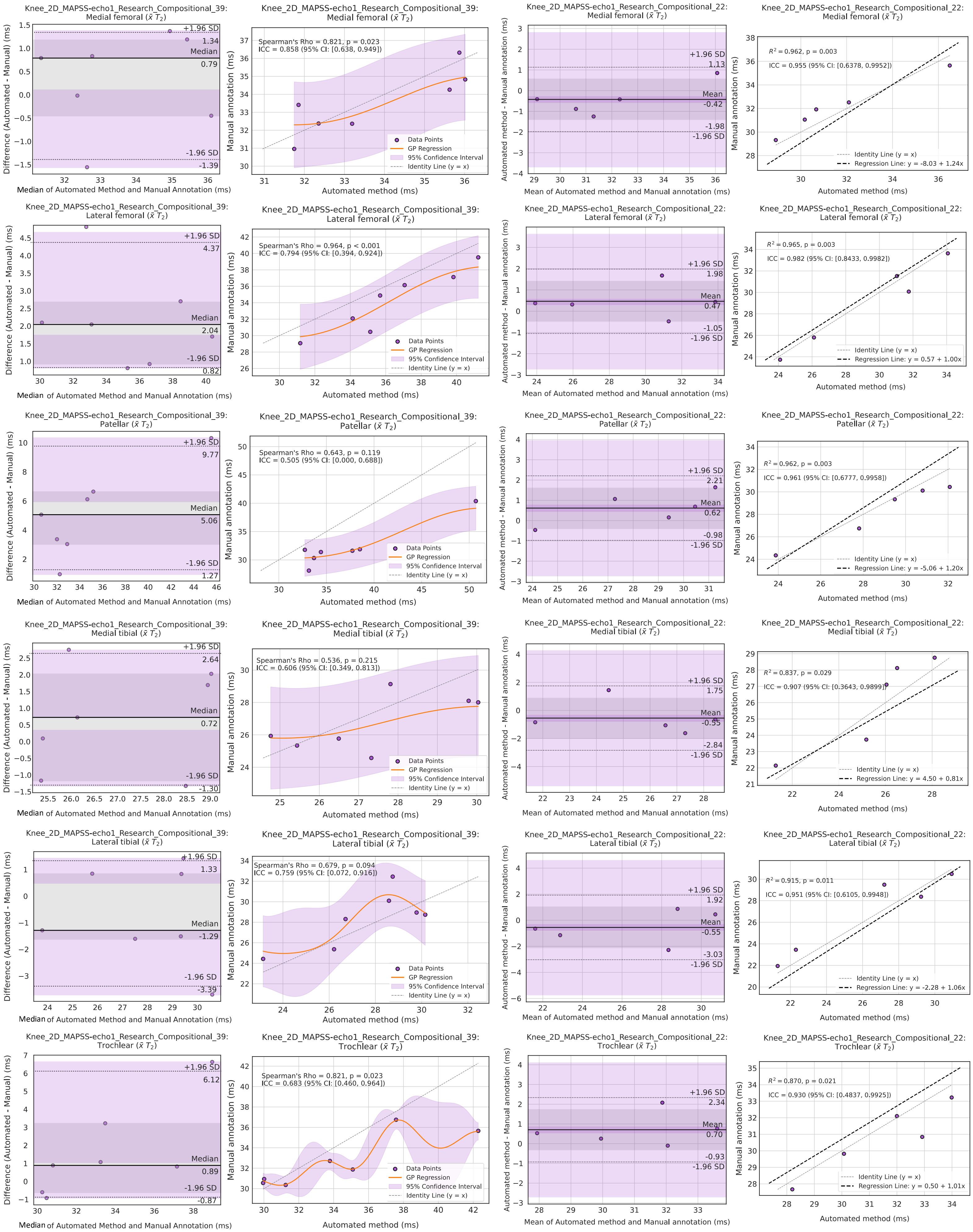

Fig. S9: T_2 Relaxation Time Agreement. This figure evaluates the agreement between manual and model-predicted T_2 relaxation time measurements at the class-label level for the Knee_2D_MAPSS- echo1_Research_Compositional_22 and Knee_2D_MAPSS-echo1_Research_Compositional_39 datasets. The Bland-Altman plots (left) and regression plots (right) reflect both normal and non-normal evaluation pipelines, with linear regression metrics (R^2, p-values, ICC3) for normal data and Gaussian Process regression metrics (Spearman's rho, bootstrapped ICC) for non-normally distributed data.

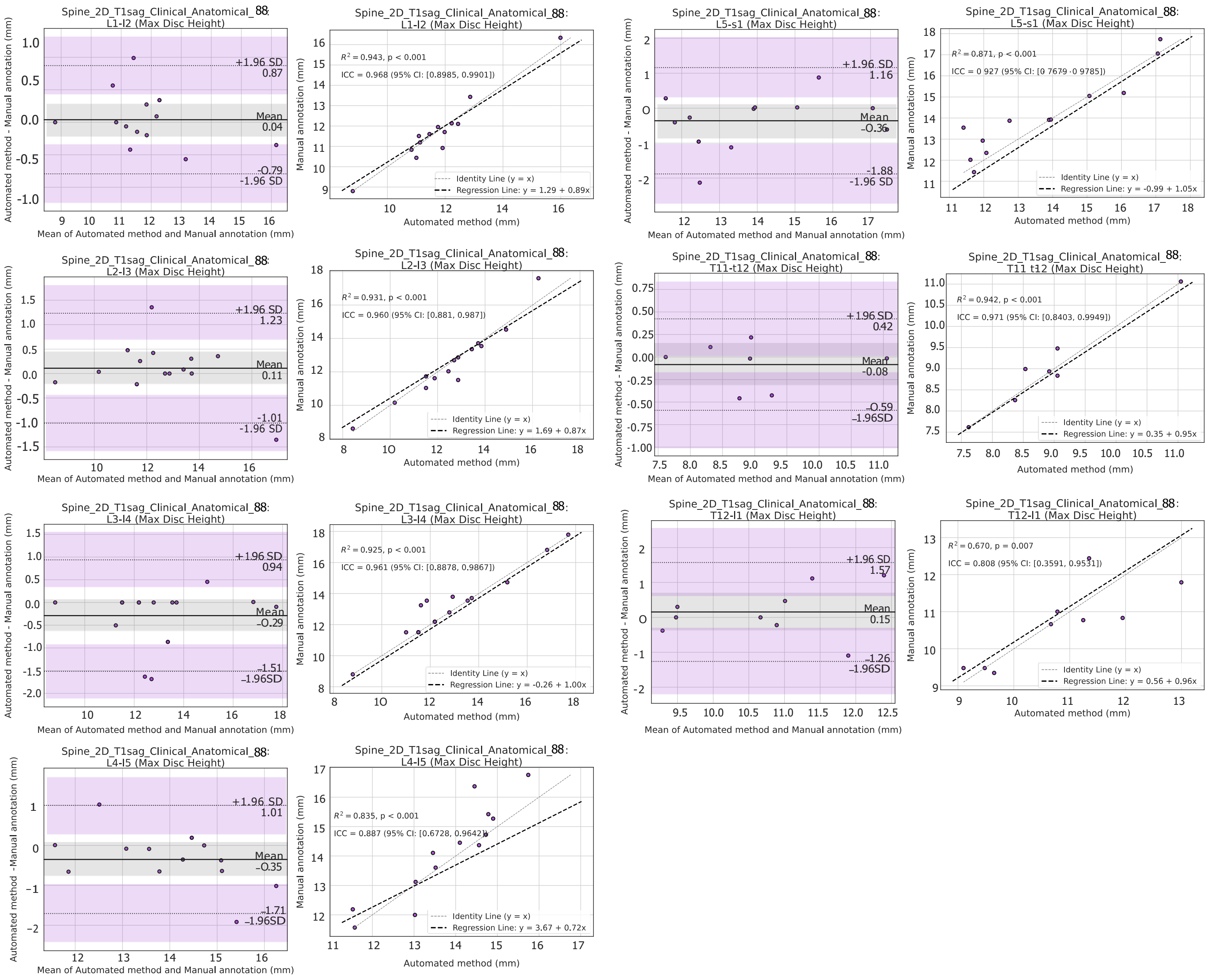

Fig. S10: Intervertebral Disc Height Agreement. This figure assesses the agreement of intervertebral disc height measurements between manual annotations and model predictions at the class-label level for the Spine_2D_T1sag_Clinical_Anatomical_88 dataset. Only normally distributed datasets are shown, evaluated through Bland-Altman plots (left) and linear regression plots (right). Statistical metrics include R^2, p-values, and ICC3 results, providing a detailed analysis of the concordance between manual and automated segmentation metrics for disc height.

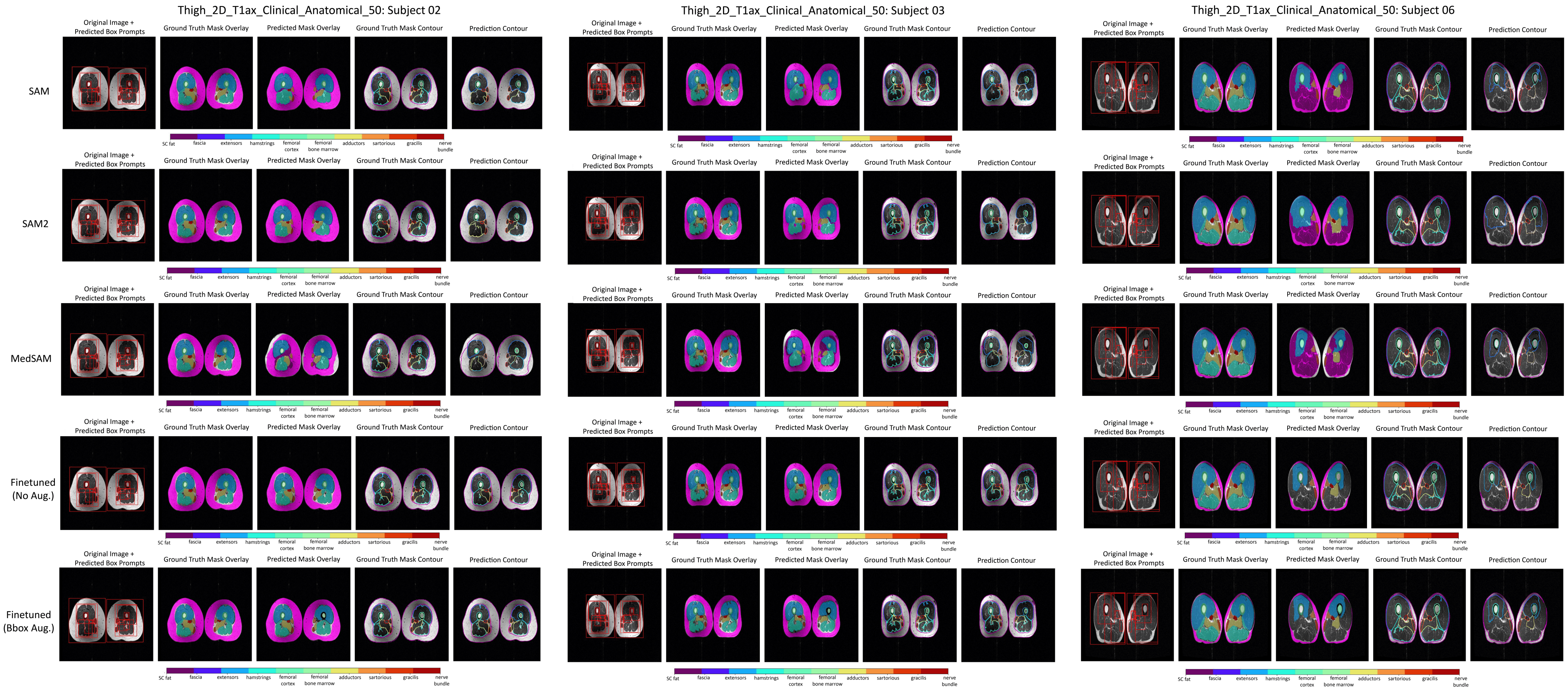

Fig. S11: Segmentation Model Performance on Thigh_2D_T1ax_Clinical_Anatomical_50. This figure compares the segmentation results of SAM, SAM2, MedSAM, Fine-tuned (No Aug.), and Fine-tuned (Bbox Aug.) models on the Thigh_2D_T1ax_Clinical_Anatomical_50 dataset. Each model is represented by five plots for three subjects, showing: (1) the original MRI slice with YOLOv8-predicted bounding boxes, (2) ground truth segmentation overlay, (3) predicted segmentation overlay, (4) ground truth contour, and (5) predicted contour. These visualizations explore the influence of imperfect bounding box prompts on segmentation model accuracy.

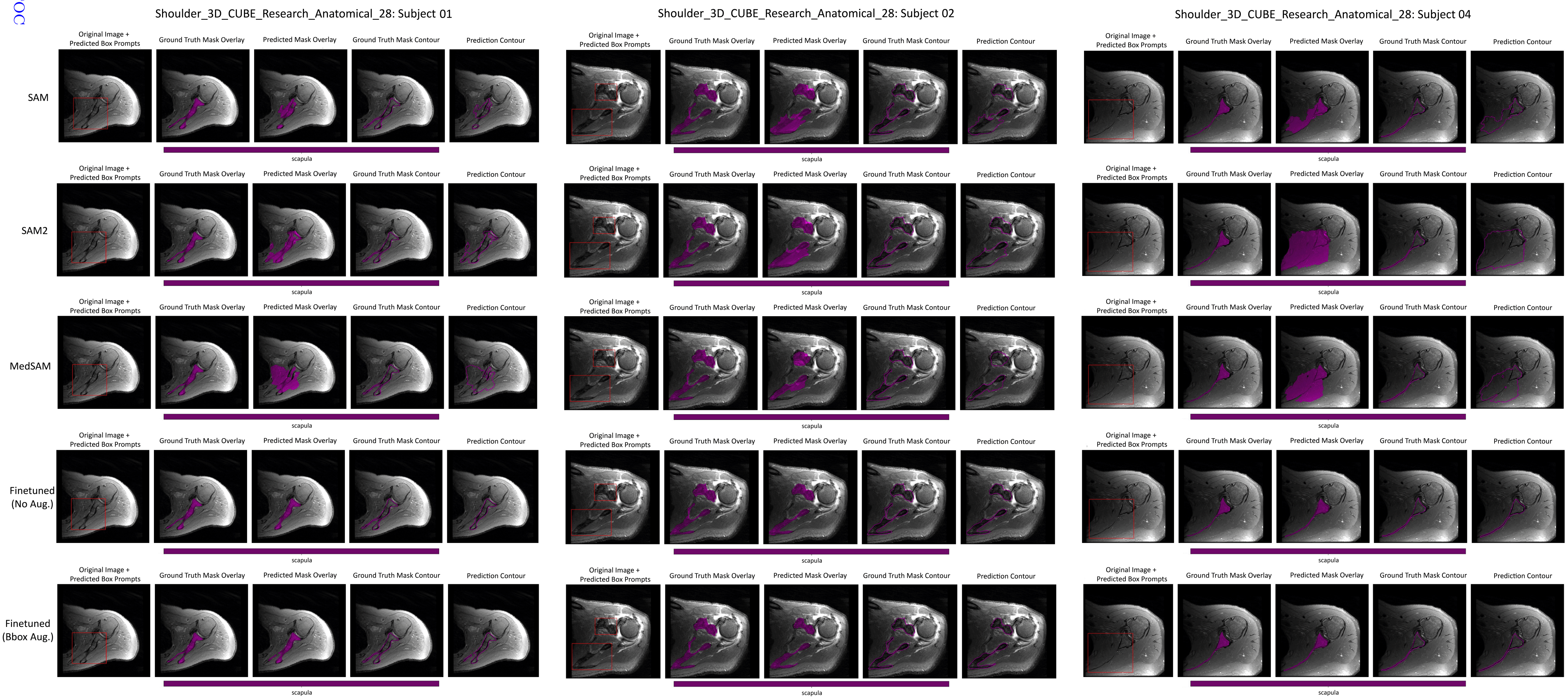

Fig. S12: Segmentation Model Performance on Shoulder_3D_CUBE_Research_Anatomical_28. This figure presents the segmentation performance of SAM, SAM2, MedSAM, Fine-tuned (No Aug.), and Fine-tuned (Bbox Aug.) models on the Shoulder_3D_CUBE_Research_Anatomical_28 dataset. For each model, five plots are shown across three subjects: original MRI with predicted bounding boxes, ground truth segmentation, predicted segmentation, and their corresponding contours. These comparisons demonstrate how bounding box prompts, generated by the YOLOv8 detection model, affect the segmentation outcomes, particularly when the prompts are not perfectly accurate.

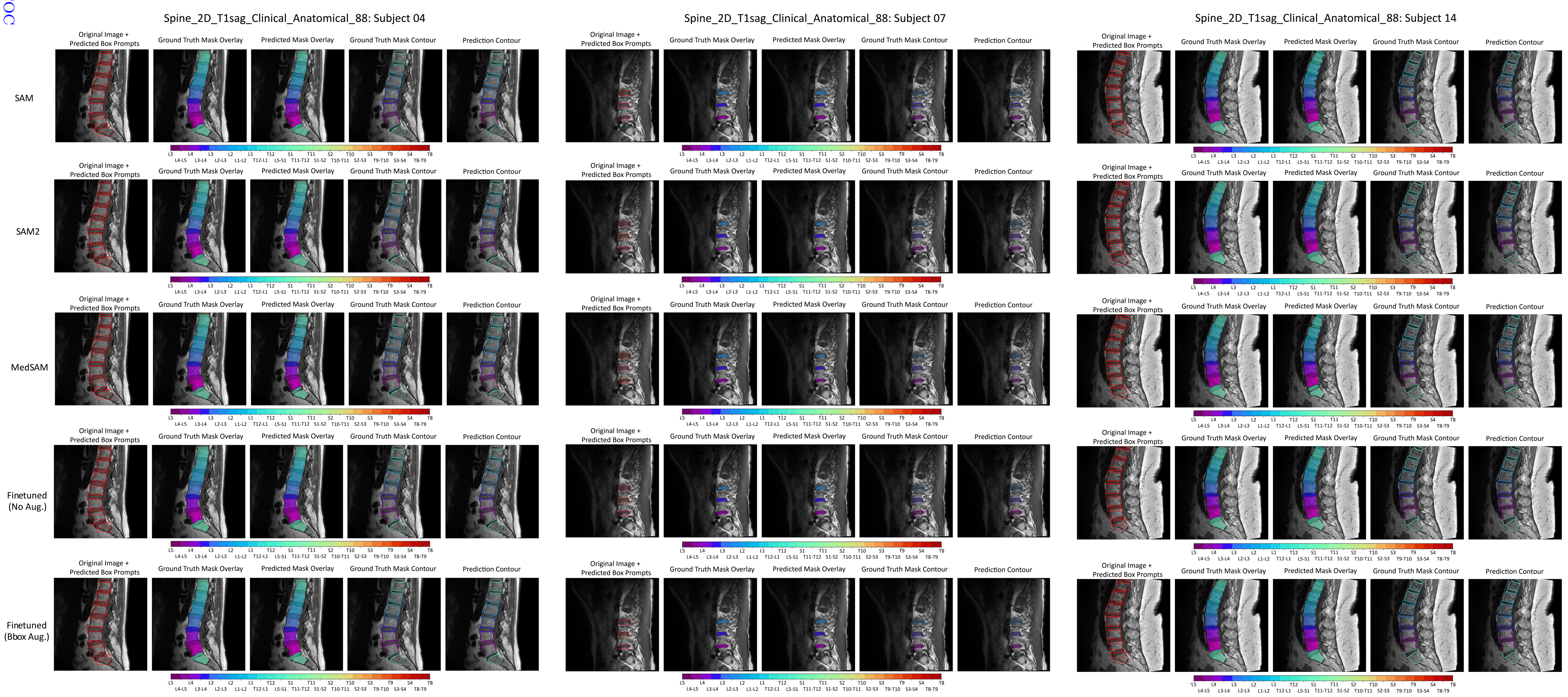

Fig. S13: Segmentation Model Performance on Spine_2D_T1sag_Clinical_Anatomical_88. This figure compares segmentation results for SAM, SAM2, MedSAM, Fine-tuned (No Aug.), and Fine-tuned (Bbox Aug.) models on the Spine_2D_T1sag_Clinical_Anatomical_88 dataset. Each model is represented by five plots across three representative subjects, including MRI slices with predicted bounding boxes, ground truth and predicted segmentation overlays, and corresponding contours. These results illustrate how inaccuracies in bounding box prompts impact the performance of the segmentation models, both in their baseline and fine-tuned forms.

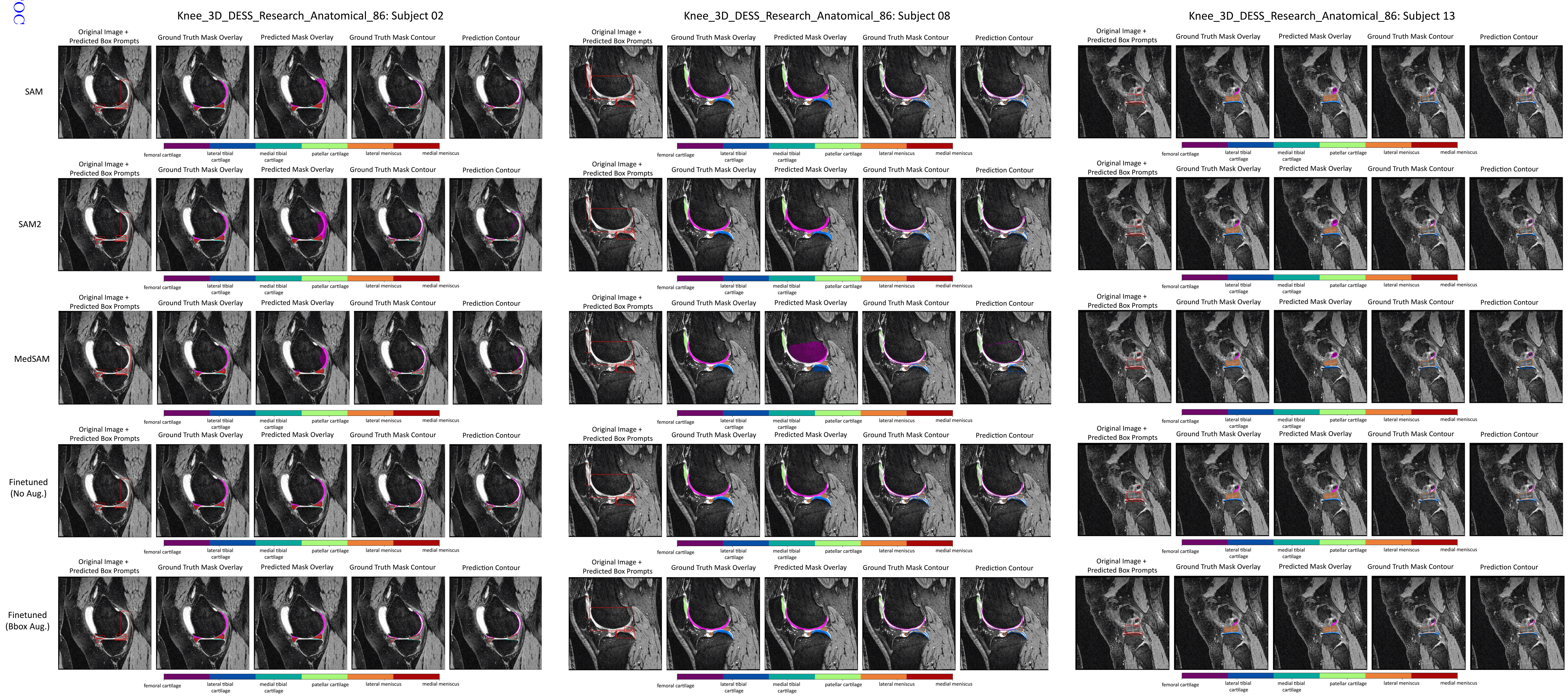

Fig. S14: Segmentation Model Performance on Knee_3D_DESS_Research_Anatomical_86. This figure showcases segmentation outcomes for SAM, SAM2, MedSAM, Fine-tuned (No Aug.), and Fine-tuned (Bbox Aug.) models on the Knee_3D_DESS_Research_Anatomical_86 dataset. Each model is presented with five plots for three subjects: MRI slices with YOLOv8-predicted bounding boxes, ground truth and predicted segmentation overlays, and corresponding contour visualizations. The figure highlights the effect of predicted bounding box prompts, which may introduce segmentation variability, especially in cases where the prompts are less accurate.

TOC

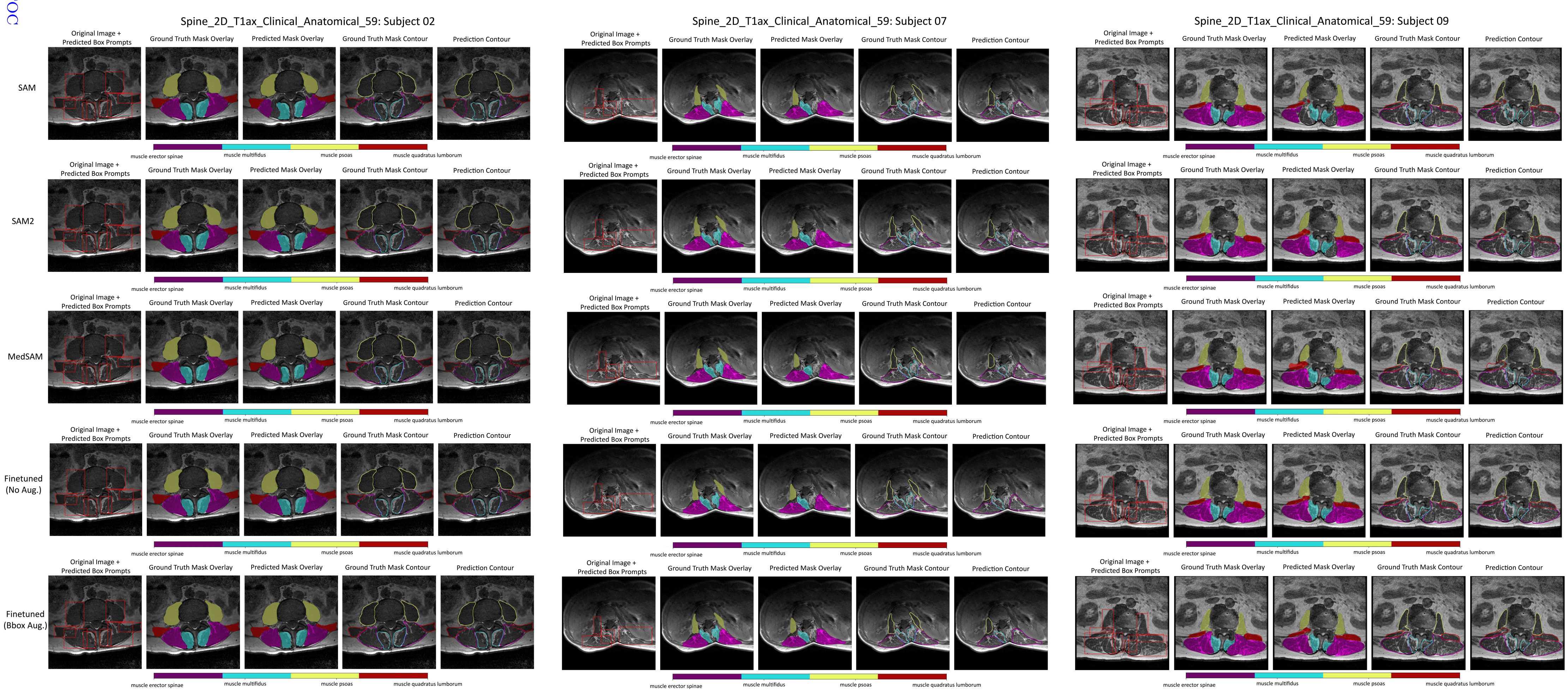

Fig. S15: Segmentation Model Performance on Spine_2D_T1ax_Clinical_Anatomical_59. This figure displays segmentation results for SAM, SAM2, MedSAM, Fine-tuned (No Aug.), and Fine-tuned (Bbox Aug.) models on the Spine_2D_T1ax_Clinical_Anatomical_59 dataset. For each model, five plots across three representative subjects show the MRI with predicted bounding boxes, ground truth segmentation, predicted segmentation, and contour overlays. These results highlight the impact of bounding box prompts generated by the object detection model, illustrating how the segmentation models respond to imperfect bounding box inputs.

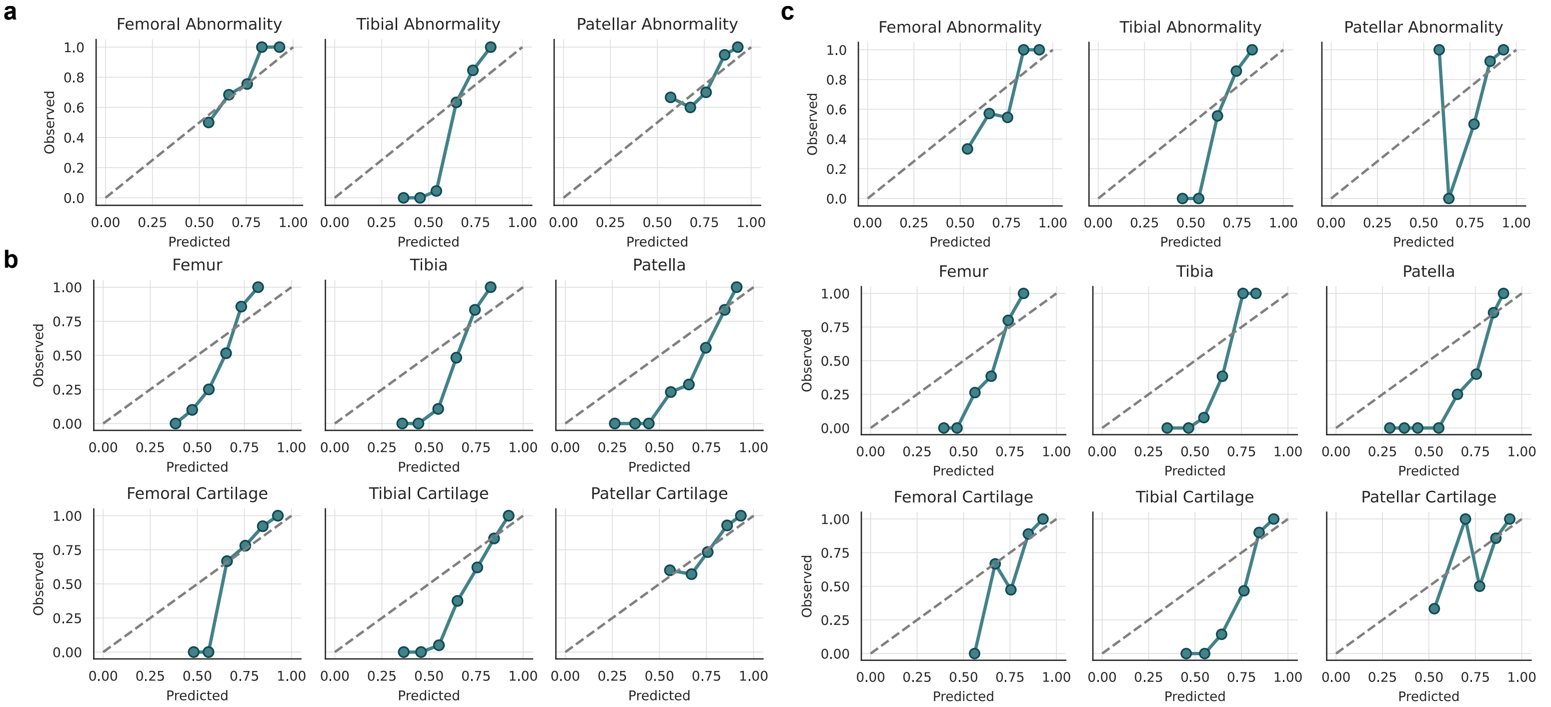

Fig. S16: Calibration performance of Stage C tasks. **a)** Joint-level abnormality (femur, tibia, patella) for the `a90p_b85p` operating point. **b)** Six tissue-specific tasks (cartilage and bone for each joint) for `a90p_b85p`. **c)** The same nine tasks for `a90p_b90p`.

Each plot shows observed event frequency vs predicted probability from the LR-XGB mean ensemble. Points derive from 10 equal-width probability bins (scikit-learn `calibration_curve`); the gray dashed line indicates perfect calibration. No probability recalibration (e.g., Platt or isotonic) was applied. Connecting lines aid visual tracking only.

TOC

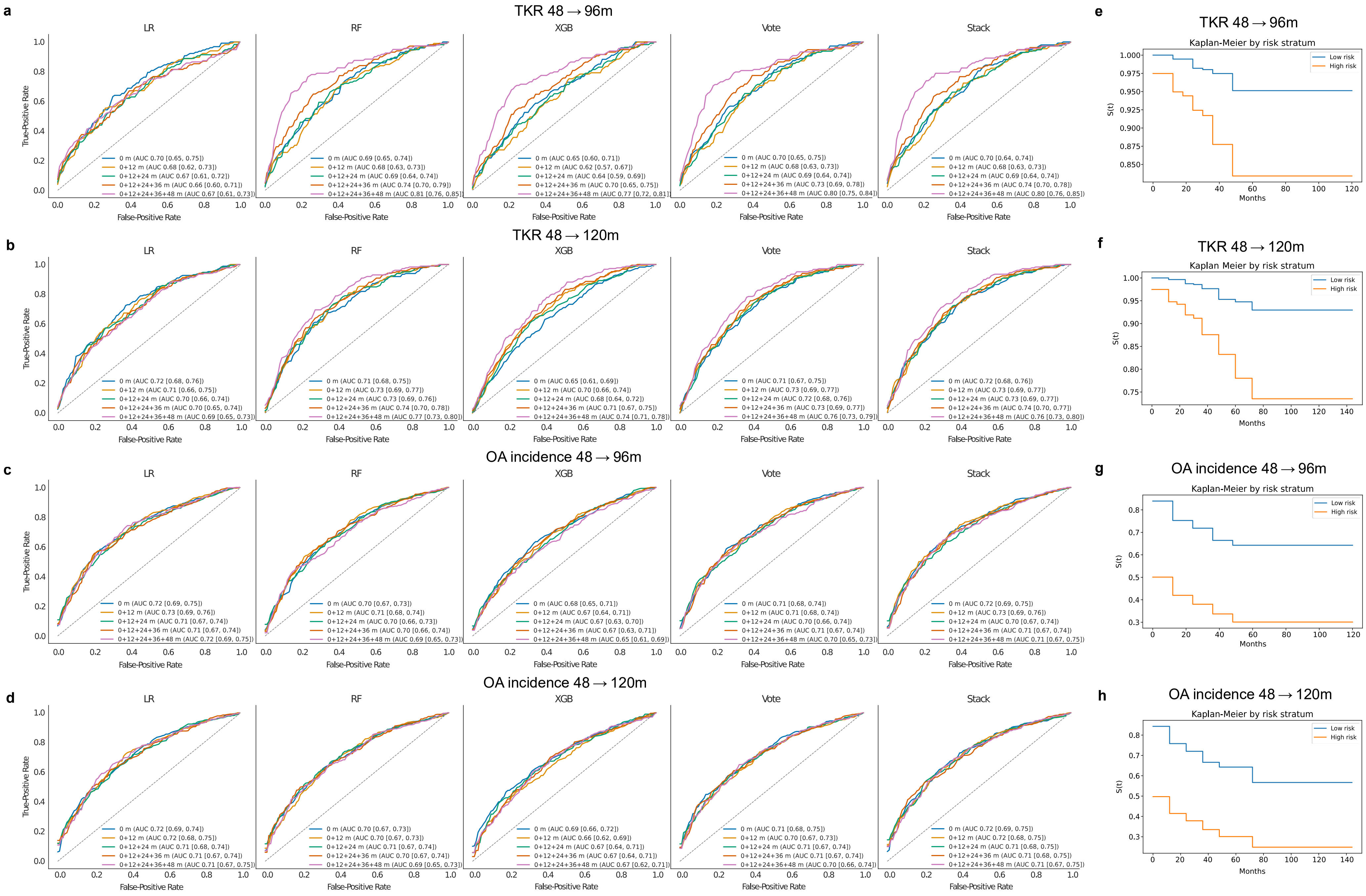

Fig. S17: ROC and survival curves. Panels a–d show ROC curves for five algorithms (LR, RF, XGB, vote, stack) evaluated at the 48-month landmark; panels e-h show Kaplan-Meier curves that stratify the test set by the RF predicted risk (median split).
a) TKR 48→96m **b)** TKR 48→120m **c)** OA incidence 48→96m **d)** OA incidence 48→120m
e–h) Corresponding survival curves demonstrate clear risk separation for TKR horizons, modest separation for OA incidence.

TOC

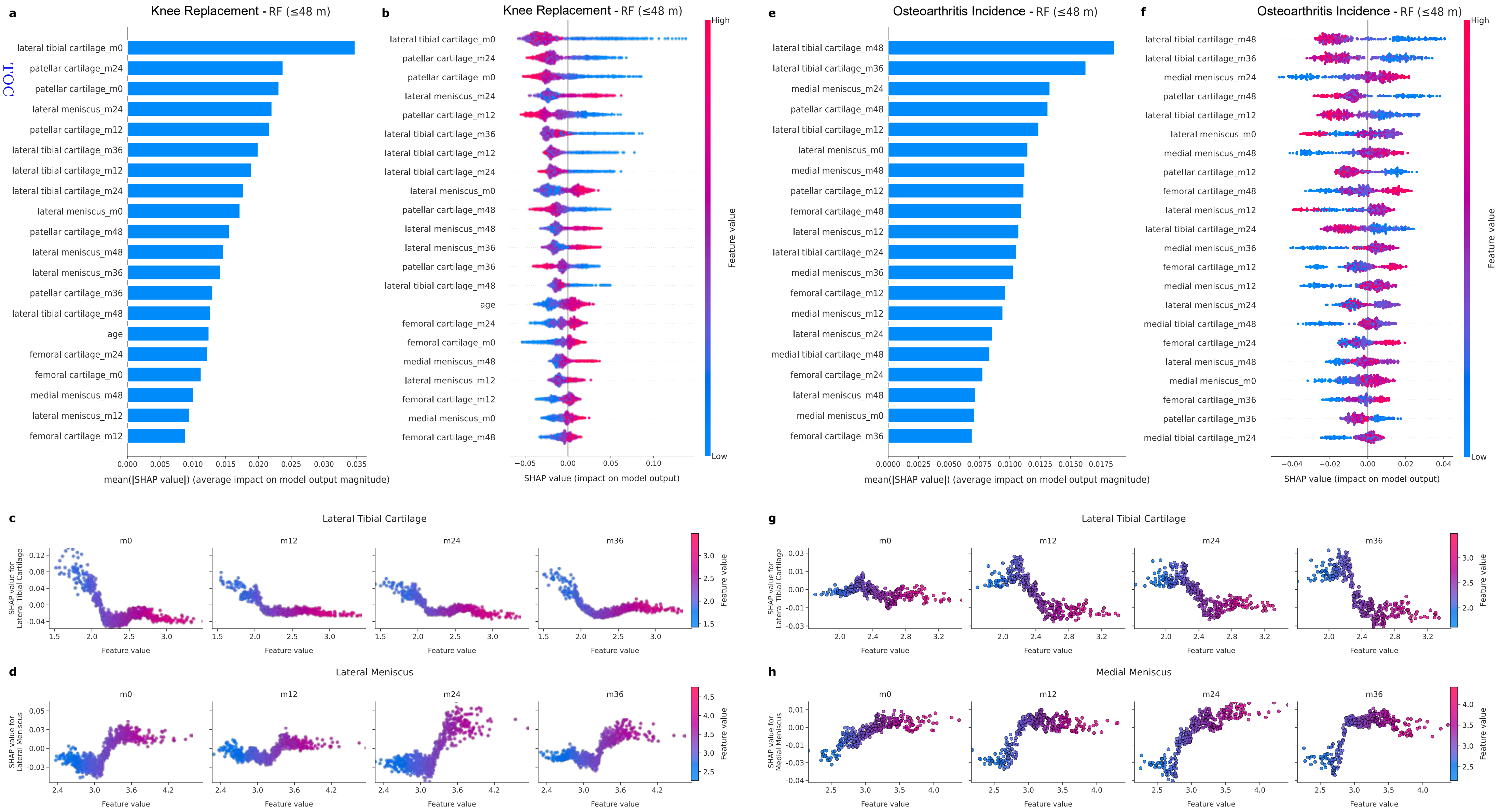

Fig. S18: SHAP interpretation of longitudinal knee biomarkers at the 48-month landmark. Panels a–d, total knee replacement (≤96 months). **a)** Ranking of features by mean absolute SHAP value for the random-forest model trained with biomarkers through month 48. **b)** Beeswarm plot of per-knee SHAP values; point colour encodes the raw feature value. **c)** One-dimensional SHAP dependence for lateral tibial cartilage thickness at baseline, 12, 24, and 36 months. **d)** Dependence for lateral meniscus thickness at the same visits. Positive SHAP values increase predicted risk. Panels e–h, incident radiographic osteoarthritis (≤48 months). **e–f)** Feature ranking and beeswarm for a random-forest model fit for visualization parity with the TKR panels; model selection for OA performance is reported in Table S22. **g)** Dependence for lateral tibial cartilage thickness. **h)** Dependence for medial meniscus thickness. All features were z-scored before modeling. SHAP values were computed on out-of-fold predictions from the five-fold scheme and then averaged across folds.

9. Tables

9.1. MRI Dataset Overview and Imaging Specifications

Summary of MRI Datasets with Anatomical Labels, Imaging Sequences, and Dimensionality

Dataset Name	*Total Subject (n)*	*Class Labels*	*Label Tissue Types*	*Anatomy*	*MRI Sequence*	*2D / 3D Sequence*	*Dataset Name*	*Total Subject (n)*	*Class Labels*	*Label Tissue Types*	*Anatomy*	*MRI Sequence*	*2D / 3D Sequence*
Knee_2D_MAPSS-echo1<br>Research_Compositional_39	39	0: 'background',<br>1: 'medial femoral',<br>2: 'lateral femoral',<br>3: 'lateral tibial',<br>4: 'medial tibial',<br>5: 'trochlear',	Cartilage	Knee	MAPSS-echo1	2D	Knee_3D_undersampled_CUBE<br>Research_Anatomical_50	50	0: 'background',<br>1: 'femoral cartilage',<br>2: 'tibial cartilage',<br>3: 'patellar cartilage',<br>4: 'femur',<br>5: 'tibia',	Bone, Cartilage	Knee	8x-undersampled<br>3D CUBE	3D
Spine_2D_T1ax<br>Clinical_Anatomical_59	59	0: 'background',<br>1: 'muscle_erector_spinae',<br>2: 'muscle_multifidus',<br>3: 'muscle_psoas',	Muscle	Lumbar Spine	T1 Axial	2D	Hip_3D_CUBE<br>Research_Anatomical_42	42	0: 'background',<br>1: 'femur',	Bone	Hip	3D CUBE	3D
Spine_2D_T1sag<br>Clinical_Anatomical_111	111	0: 'background',<br>1: 'vertebral_bodies',<br>2: 'intervetebral_discs'	Bone, Cartilage	Lumbar Spine	T1 Sagittal	2D	Knee_3D_DESS<br>Research_Anatomical_86	86	0: 'background',<br>1: 'femoral cartilage',<br>2: 'lateral tibial cartilage',<br>3: 'medial tibial cartilage',<br>4: 'patellar cartilage',<br>5: 'lateral meniscus',<br>6: 'medial meniscus'	Cartilage	Knee	3D DESS	2D
Spine_2D_T2ax<br>Clinical_Anatomical_38	38	0: 'background',<br>1: 'vertebral_body',<br>2: 'intervertebral_disc',<br>3: 'dural_sac'	Bone, Cartilage	Lumbar Spine	T2 Axial	2D	Thigh_2D_T1ax<br>Clinical_Anatomical_50	50	0: 'background',<br>1: 'sc_fat',<br>2: 'fascia',<br>3: 'extensors',<br>4: 'hamstrings',<br>5: 'fem_cortex',<br>6: 'fem_bm',<br>7: 'adductors',<br>8: 'sartorius',<br>9: 'gracilis',<br>10: 'nv'	Bone, Muscle,<br>Fat, Nerve	Thigh	T1 Axial	2D
Spine_2D_T1sag<br>Clinical_Anatomical_88	88	0: 'background',<br>9: 'L1', 7: 'L2',<br>5: 'L3', 3: 'L4',<br>1: 'L5',<br>8: 'L1-L2', 6: 'L2-L3',<br>4: 'L3-L4', 2: 'L4-L5',<br>12: 'L5-S1',<br>27: 'T8', 23: 'T9',<br>19: 'T10', 15: 'T11',<br>11: 'T12',<br>13: 'S1', 17: 'S2',<br>21: 'S3', 25: 'S4',<br>26: 'T8-T9', 22: 'T9-T10',<br>18: 'T10-T11',<br>14: 'T11-T12',<br>10: 'T12-L1',<br>16: 'S1-S2', 20: 'S2-S3',<br>24: 'S3-S4'	Bone, Cartilage	Lumbar Spine	T1 Sagittal	2D	Knee_2D_MAPSS-echo1<br>Research_Compositional_22	22	0: 'background',<br>1: 'medial femoral',<br>2: 'lateral femoral',<br>3: 'lateral tibial',<br>4: 'medial tibial',<br>5: 'trochlear',<br>6: 'patellar'	Cartilage	Knee	MAPSS-echo1	2D
Shoulder_3D_CUBE<br>Research_Anatomical_28	28	0: 'background',<br>1: 'scapula'	Bone	Shoulder	3D CUBE	3D	Knee_3D_CUBE<br>Research_Anatomical_300	300	0: 'background',<br>1: 'femoral cartilage',<br>2: 'tibial cartilage',<br>3: 'patellar cartilage',<br>4: 'femur',<br>5: 'tibia',<br>6: 'patella'	Bone, Cartilage	Knee	3D CUBE	3D

Table S0: Summary of MRI Datasets with Anatomical Labels, Imaging Sequences, and Dimensionality. This table summarizes the MRI datasets used in the study, which may serve as a reference for the dataset names, number of subjects, anatomical labels and types, MRI sequences, and dimensionality (2D or 3D). For each dataset, class labels corresponding to anatomical structures such as cartilage, muscles, and bones are included to ensure clarity in segmentation. The datasets are organized by imaged anatomy, MRI sequence type, and dataset size. The naming convention follows the structure "Anatomy, 2D/3D, MRI sequence, Clinical/Research, Anatomical/Compositional, Dataset Sample Size" to offer a consistent reference point throughout the study.

Demographic Summary of Musculoskeletal MRI Datasets: Age, Weight, and Sex Distribution

Dataset	*Age (Mean ± SD)*	*Weight (kg, Mean ± SD)*	*Female (n, %)*	*Male (n, %)*	*Other (n, %)*	*Total (n)*
Knee_2D_MAPSS-echo1_Research_Compositional_39	28.46 ± 19.17	73.91 ± 12.31	20 (51.28%)	19 (48.72%)	--	39
Spine_2D_T1ax_Clinical_Anatomical_59	57.46 ± 14.83	78.90 ± 20.30	35 (59.32%)	24 (40.68%)	--	59
Spine_2D_T1sag_Clinical_Anatomical_111	58.46 ± 16.66	76.95 ± 19.07	62 (55.86%)	49 (44.14%)	--	111
Spine_2D_T2ax_Clinical_Anatomical_38	60.97 ± 16.99	78.38 ± 25.22	23 (60.53%)	15 (39.47%)	--	38
Spine_2D_T1sag_Clinical_Anatomical_88	59.93 ± 7.88	78.36 ± 20.22	54 (63.53%)	31 (36.47%)	3 (1.00%)	88
Shoulder_3D_CUBE_Research_Anatomical_28	51.71 ± 15.55	84.43 ± 16.69	10 (35.71%)	18 (64.29%)	--	28
Knee_3D_undersampled_CUBE_Research_Anatomical_50	44.54 ± 14.66	70.49 ± 16.78	26 (52.00%)	24 (48.00%)	--	50
Hip_3D_CUBE_Research_Anatomical_42	54.21 ± 12.95	71.71 ± 13.82	19 (45.24%)	23 (54.76%)	--	42
Knee_3D_DESS_Research_Anatomical_86	61.60 ± 9.80	89.77 ± 15.50	43 (50.00%)	43 (50.00%)	--	86
Thigh_2D_T1ax_Clinical_Anatomical_50	58.88 ± 10.00	80.51 ± 14.87	21 (42.00%)	29 (58.00%)	--	50
Knee_2D_MAPSS-echo1_Research_Compositional_22	26.86 ± 8.20	78.39 ± 17.32	8 (36.36%)	14 (63.64%)	--	22
Knee_3D_CUBE_Research_Anatomical_300	44.27 ± 13.92	75.65 ± 14.93	140 (46.67%)	160 (53.33%)	--	300

Anatomical Group	*Age (Mean ± SD)*	*Weight (kg, Mean ± SD)*	*Female (n, %)*	*Male (n, %)*	*Other (n, %)*	*Total (n)*
Knee	41.15 ± 14.18	77.64 ± 7.36	237 (47.69%)	260 (52.31%)	0 (0.00%)	497
Lumbar Spine	59.21 ± 1.55	78.15 ± 0.84	174 (58.78%)	119 (40.20%)	3 (1.01%)	296
Thigh	58.88 ± 10.00	80.51 ± 14.87	21 (42.00%)	29 (58.00%)	0 (0.00%)	50
Hip	54.21 ± 12.95	71.71 ± 13.82	19 (45.24%)	23 (54.76%)	0 (0.00%)	42
Shoulder	51.71 ± 15.55	84.43 ± 16.69	10 (35.71%)	18 (64.29%)	0 (0.00%)	28
						Total MSK Subjects: 913

Table S1: Demographic Summary of Musculoskeletal MRI Datasets. This table provides an overview of the age, weight, and sex distribution across different datasets. Sample sizes (n) represent distinct subjects. Age and weight are presented as mean ± standard deviation (SD), and sex distribution is shown as counts and percentages.

TOC

MRI Imaging Parameters and Scanner Specifications for Musculoskeletal Datasets

Dataset	*Anatomy*	*Imaging Parameters*											*Scanner Info*			
		MRI Sequence	*2D / 3D Sequence*	*(TE / TR / SAR)*	*Slice Spacing*	*Pixel Spacing*	*Slice Thickness*	*Percent Phase FOV*	*Image Size (Rows x Columns)*	*Field Strength*	*Flip Angle*	*ETL*	*Scanner Vendor*	*Scanner Model*	*Scanner Name*	*Receive Coil*
Knee_2D_MAPSS-echo1_Research_Compositional_39	Knee	MAPSS-echo1	2D	0.00 / 5.40 / --	4.00	['0.5469', '0.5469']	4.00	100	256 x 256	3.00	60	1	GE Healthcare	--	MR30OW	HD TR Knee PA
Knee_3D_undersampled_CUBE_Research_Anatomical_50	Knee	8x_undersampled_3D_CUBE	3D	29.00 / 1002.00 / 0.09	--	['0.293', '0.293']	0.60	--	512 x 512	3.00	90	36	GE Healthcare	--	GE Discovery	18Ch Knee
Knee_3D_DESS_Research_Anatomical_86	Knee	3D DESS	2D	4.71 / 16.32 / 0.00	--	['0.3646', '0.3646']	0.70	100	384 x 384	2.89	25	1	Siemens	--	Trio	quadrature transmit-receive knee coils (USA Instruments, Aurora, OH)
Knee_2D_MAPSS-echo1_Research_Compositional_22	Knee	MAPSS-echo1	2D	0.00 / 8.73 / --	4.00	['0.5469', '0.5469']	4.00	100	256 x 256	3.00	60	1	GE Healthcare	UC-CB3TW	DISCOVERY MR750w	HD TR Knee PA
Knee_3D_CUBE_Research_Anatomical_300	Knee	3D CUBE	3D	29.00 / 1002.00 / 0.09	--	['0.293', '0.293']	0.60	--	512 x 512	3.00	90	36	GE Healthcare	--	GE Discovery	18Ch Knee
Spine_2D_T1ax_Clinical_Anatomical_59	Lumbar Spine	T1 Axial	2D	12.64 / 670.11 / 2.22	4.06	['0.3516', '0.3516']	4.00	100	512 x 512	1.5 / 3	90	4	GE Healthcare	mrc1	Signa HDxt	HNS CTL456
Spine_2D_T1sag_Clinical_Anatomical_111	Lumbar Spine	T1 Sagittal	2D	11.05 / 620.24 / 2.47	3.05	['0.4883', '0.4883']	3.00	100	512 x 512	1.5 / 3	90	4	GE Healthcare	mrc2	Signa HDxt	HNS CTL456
Spine_2D_T2ax_Clinical_Anatomical_38	Lumbar Spine	T2 Axial	2D	85.95 / 5893.83 / 2.39	3.96	['0.3516', '0.3516']	4.00	100	512 x 512	1.5 / 3	90	16	GE Healthcare	mrc1	Signa HDxt	HNS CTL456
Spine_2D_T1sag_Clinical_Anatomical_88	Lumbar Spine	T1 Sagittal	2D	8.62 / 736.76 / --	3.00	[0.6771, 0.6771]	3.00	--	384 x 384	3.00	125	4	GE Healthcare	AWP45205	Skyra	60PA
Shoulder_3D_CUBE_Research_Anatomical_28	Shoulder	3D_CUBE	3D	46.85 / 1352.00 / 1.58	0.50	['0.375', '0.375']	1.00	90	512 x 512	3.00	90	30	GE Healthcare	OIMR1	DISCOVERY MR750	GEM Flex Medium
Hip_3D_CUBE_Research_Anatomical_42	Hip	3D_CUBE	3D	20.50 / 1201.19 / 1.52	0.40	['0.3521', '0.3521']	0.80	220	512 x 512	3.00	90	30	GE Healthcare	UCSFCBMR7	SIGNA Premier	30AA+60PA
Thigh_2D_T1ax_Clinical_Anatomical_50	Thigh	T1 Axial	2D	10.00 / 582.00 / 0.65	5.00	['0.9766', '0.9766']	5.00	50	256 x 512	2.89	90	1	Siemens	--	Trio	quadrature transmit-receive knee coils (USA Instruments, Aurora, OH)

TE = Time to Echo

TR = Repitition Time

SAR = Specific Absorption Rate

ETL = Echo Train Length

Table S2: MRI Imaging Parameters and Scanner Specifications for Musculoskeletal Datasets. This table provides a detailed summary of the MRI imaging parameters and scanner specifications for the musculoskeletal (MSK) datasets used in this study. Each entry corresponds to a specific dataset and anatomical region (knee, lumbar spine, hip, shoulder, or thigh). Key imaging parameters are listed, including MRI sequence type, dimensionality (2D/3D), echo time (TE), repetition time (TR), and specific absorption rate (SAR). Acquisition settings such as slice spacing, pixel spacing, slice thickness, percent phase field of view (FOV), and image resolution (rows $\times$ columns) are also detailed. Also, the table includes field strength, flip angle, and echo train length (ETL), parameters that affect image quality and acquisition duration. In this way, this table outlines the range of MRI parameters across datasets, and offers a clear perspective on the imaging diversity within the study.

Comparison of Mean DICE Scores for Musculoskeletal MRI Datasets Using MedSAM, SAM, and SAM2 Base Model Weights			
Dataset	*Mean DICE Score*		
	MedSAM	*SAM*	*SAM2*
Knee_2D_MAPSS-echo1_Research_Compositional_39	0.303	0.481	**0.486**
Shoulder_3D_CUBE_Research_Anatomical_28	0.320	0.547	**0.680**
Knee_3D_undersampled_CUBE_Research_Anatomical_50	0.618	**0.738**	0.719
Hip_3D_CUBE_Research_Anatomical_42	0.890	**0.943**	0.927
Knee_3D_DESS_Research_Anatomical_86	0.488	0.610	**0.624**
Thigh_2D_T1ax_Clinical_Anatomical_50	0.580	0.831	**0.843**
Knee_2D_MAPSS-echo1_Research_Compositional_22	0.304	**0.589**	0.572
Knee_3D_CUBE_Research_Anatomical_300	0.627	**0.774**	0.739
Spine_2D_T1ax_Clinical_Anatomical_59	0.803	0.866	**0.877**
Spine_2D_T1sag_Clinical_Anatomical_111	0.926	**0.929**	0.928
Spine_2D_T2ax_Clinical_Anatomical_38	0.859	**0.899**	0.883
Spine_2D_T1sag_Clinical_Anatomical_88	0.899	0.916	**0.917**
Musculoskeletal Mean DICE:	0.635	0.760	**0.766**
Knee Set Mean DICE:	0.468	**0.638**	0.628
Spine Set Mean DICE:	0.872	**0.902**	0.901

Table S3: Comparison of Mean DICE Scores for Musculoskeletal MRI Datasets Using MedSAM, SAM, and SAM2 Base Model Weights. This table presents a comparative analysis of mean DICE scores (unitless; range 0–1) across musculoskeletal MRI datasets, evaluating the performance of three segmentation models: MedSAM, SAM, and SAM2. The table lists the datasets used, covering various anatomical regions, including the knee, shoulder, hip, thigh, and spine. For each dataset, the mean DICE score is provided for MedSAM, a model adapted for medical imaging, SAM, a general-purpose segmentation model, and SAM2, an updated version of SAM with extended training on both natural images and video. The overall musculoskeletal mean DICE scores, along with the specific mean scores for the knee and spine datasets, are also included. These metrics offer a clear comparison of each model's segmentation accuracy across different anatomical regions and imaging protocols.

Friedman Test Results with Benjamini-Hochberg Correction for Statistical Significance of DICE Scores Across Base Models (MedSAM, SAM, SAM2)			
Dataset	*Friedman Test*		
	Statistic	*P-Value*	*P-Value (BH FDR)*
Knee_2D_MAPSS-echo1_Research_Compositional_39	1.06E+01	5.06E-03	1.01E-02
Knee_2D_MAPSS-echo1_Research_Compositional_22	8.40E+00	1.50E-02	2.00E-02
Shoulder_3D_CUBE_Research_Anatomical_28	8.00E+00	1.83E-02	2.20E-02
Knee_3D_DESS_Research_Anatomical_86	2.11E+01	2.56E-05	1.54E-04
Knee_3D_undersampled_CUBE_Research_Anatomical_50	1.30E+01	1.50E-03	3.61E-03
Knee_3D_CUBE_Research_Anatomical_300	8.11E+01	2.44E-18	2.93E-17
Thigh_2D_T1ax_Clinical_Anatomical_50	1.40E+01	9.12E-04	2.74E-03
Spine_2D_T1ax_Clinical_Anatomical_59	1.68E+01	2.25E-04	8.99E-04
Spine_2D_T2ax_Clinical_Anatomical_38	3.43E+00	1.80E-01	1.80E-01
Spine_2D_T1sag_Clinical_Anatomical_88	8.40E+00	1.50E-02	2.00E-02
Hip_3D_CUBE_Research_Anatomical_42	8.86E+00	1.19E-02	2.00E-02
Spine_2D_T1sag_Clinical_Anatomical_111	4.30E+00	1.16E-01	1.27E-01

Table S4: Friedman Test Results with Benjamini-Hochberg FDR Correction for DICE Scores Across Base Models. This table presents the results of the Friedman test, conducted to assess statistically significant differences in mean DICE scores across three segmentation models (MedSAM, SAM, SAM2) for each dataset. The DICE scores represent the mean performance across all segmentation labels and test set subjects within each dataset. The Friedman test was applied to repeated measures for each subject, comparing the same individuals across the three models. The test is two-sided and assesses whether there are significant differences in the DICE score distributions between the models.

For each dataset, the table provides the Friedman test statistic (following a chi-squared distribution), the degrees of freedom (df), the unadjusted p-value, and the Benjamini-Hochberg FDR-adjusted p-value. The FDR correction was applied to control the false discovery rate due to multiple comparisons. A corrected p- value below the significance threshold ($\alpha = 0.05$) indicates a statistically significant difference in DICE scores between the models for the respective dataset.

Wilcoxon Rank Sum Test with Benjamini-Hochberg Correction for Pairwise Comparisons of DICE Scores Among MedSAM, SAM, and SAM2 Across Musculoskeletal Datasets				
Dataset	*Wilcoxon Rank Sum Test*			
	Comparison	*U-Statistic*	*P-Value*	*P-Value (BH FDR)*
Knee_2D_MAPSS-echo1_Research_Compositional_39	SAM vs MedSAM	0.00E+00	1.56E-02	3.31E-02
Knee_2D_MAPSS-echo1_Research_Compositional_39	SAM vs SAM2	1.30E+01	9.38E-01	9.38E-01
Knee_2D_MAPSS-echo1_Research_Compositional_39	MedSAM vs SAM2	0.00E+00	1.56E-02	3.31E-02
Knee_2D_MAPSS-echo1_Research_Compositional_22	SAM vs MedSAM	0.00E+00	6.25E-02	1.07E-01
Knee_2D_MAPSS-echo1_Research_Compositional_22	SAM vs SAM2	5.00E+00	6.25E-01	6.82E-01
Knee_2D_MAPSS-echo1_Research_Compositional_22	MedSAM vs SAM2	0.00E+00	6.25E-02	1.07E-01
Shoulder_3D_CUBE_Research_Anatomical_28	SAM vs MedSAM	0.00E+00	1.25E-01	1.72E-01
Shoulder_3D_CUBE_Research_Anatomical_28	SAM vs SAM2	0.00E+00	1.25E-01	1.72E-01
Shoulder_3D_CUBE_Research_Anatomical_28	MedSAM vs SAM2	0.00E+00	1.25E-01	1.72E-01
Knee_3D_DESS_Research_Anatomical_86	SAM vs MedSAM	0.00E+00	1.22E-04	8.79E-04
Knee_3D_DESS_Research_Anatomical_86	SAM vs SAM2	4.10E+01	5.02E-01	5.64E-01
Knee_3D_DESS_Research_Anatomical_86	MedSAM vs SAM2	0.00E+00	1.22E-04	8.79E-04
Knee_3D_undersampled_CUBE_Research_Anatomical_50	SAM vs MedSAM	0.00E+00	7.81E-03	2.34E-02
Knee_3D_undersampled_CUBE_Research_Anatomical_50	SAM vs SAM2	7.00E+00	1.48E-01	1.91E-01
Knee_3D_undersampled_CUBE_Research_Anatomical_50	MedSAM vs SAM2	0.00E+00	7.81E-03	2.34E-02
Knee_3D_CUBE_Research_Anatomical_300	SAM vs MedSAM	0.00E+00	5.68E-14	1.02E-12
Knee_3D_CUBE_Research_Anatomical_300	SAM vs SAM2	2.00E+01	2.11E-11	2.53E-10
Knee_3D_CUBE_Research_Anatomical_300	MedSAM vs SAM2	0.00E+00	5.68E-14	1.02E-12
Thigh_2D_T1ax_Clinical_Anatomical_50	SAM vs MedSAM	0.00E+00	3.91E-03	1.56E-02
Thigh_2D_T1ax_Clinical_Anatomical_50	SAM vs SAM2	9.00E+00	1.29E-01	1.72E-01
Thigh_2D_T1ax_Clinical_Anatomical_50	MedSAM vs SAM2	0.00E+00	3.91E-03	1.56E-02
Spine_2D_T1ax_Clinical_Anatomical_59	SAM vs MedSAM	0.00E+00	1.95E-03	1.00E-02
Spine_2D_T1ax_Clinical_Anatomical_59	SAM vs SAM2	3.00E+00	9.77E-03	2.70E-02
Spine_2D_T1ax_Clinical_Anatomical_59	MedSAM vs SAM2	0.00E+00	1.95E-03	1.00E-02
Spine_2D_T2ax_Clinical_Anatomical_38	SAM vs MedSAM	3.00E+00	7.81E-02	1.28E-01
Spine_2D_T2ax_Clinical_Anatomical_38	SAM vs SAM2	7.00E+00	2.97E-01	3.45E-01
Spine_2D_T2ax_Clinical_Anatomical_38	MedSAM vs SAM2	5.00E+00	1.56E-01	1.94E-01
Spine_2D_T1sag_Clinical_Anatomical_88	SAM vs MedSAM	1.70E+01	1.25E-02	3.20E-02
Spine_2D_T1sag_Clinical_Anatomical_88	SAM vs SAM2	5.60E+01	8.47E-01	8.94E-01
Spine_2D_T1sag_Clinical_Anatomical_88	MedSAM vs SAM2	1.40E+01	6.71E-03	2.34E-02
Hip_3D_CUBE_Research_Anatomical_42	SAM vs MedSAM	0.00E+00	1.56E-02	3.31E-02
Hip_3D_CUBE_Research_Anatomical_42	SAM vs SAM2	4.00E+00	1.09E-01	1.71E-01
Hip_3D_CUBE_Research_Anatomical_42	MedSAM vs SAM2	1.00E+00	3.13E-02	6.25E-02
Spine_2D_T1sag_Clinical_Anatomical_111	SAM vs MedSAM	7.50E+01	2.77E-01	3.33E-01
Spine_2D_T1sag_Clinical_Anatomical_111	SAM vs SAM2	5.30E+01	5.32E-02	1.01E-01
Spine_2D_T1sag_Clinical_Anatomical_111	MedSAM vs SAM2	1.00E+02	8.69E-01	8.94E-01

Table S5: Wilcoxon Rank Sum Test with Benjamini-Hochberg FDR Correction for Pairwise Comparisons of DICE Scores Among MedSAM, SAM, and SAM2 Across Musculoskeletal Datasets. This table presents the results of the Wilcoxon signed-rank test, comparing the distribution of DICE scores between model pairs (MedSAM, SAM, SAM2) across musculoskeletal datasets. DICE scores represent the mean values across all segmentation labels and test set subjects within each dataset. Pairwise comparisons were conducted between SAM vs. MedSAM, SAM vs. SAM2, and MedSAM vs. SAM2 for each dataset. The table reports the Wilcoxon test statistic, unadjusted p-values, and Benjamini-Hochberg FDR-adjusted p-values. A corrected p-value below $\alpha = 0.05$ indicates a statistically significant difference in DICE scores between models for the corresponding dataset.

Finetuning Experiments of SAM, MedSAM, and SAM2 Models on Musculoskeletal MRI Datasets

Segment Anything (SAM)			MedSAM			SAM2		
Experiment Name	*Experiment Type*	*Experimental Dataset Grouping*	*Experiment Name*	*Experiment Type*	*Experimental Dataset Grouping*	*Experiment Name*	*Experiment Type*	*Experimental Dataset Grouping*
SAM	0	N/A	MedSAM	0	N/A	SAM2	0	N/A
shoulder_CUBE_SAM	1	Shoulder_3D_CUBE_Research_Anatomical_28	shoulder_CUBE_MedSAM	1	Shoulder_3D_CUBE_Research_Anatomical_28	shoulder_CUBE_SAM2	1	Shoulder_3D_CUBE_Research_Anatomical_28
knee_MAPSS_SAM	1	Knee_2D_MAPSS-echo1_Research_Compositional_22,<br>Knee_2D_MAPSS-echo1_Research_Compositional_39	knee_MAPSS_MedSAM	1	Knee_2D_MAPSS-echo1_Research_Compositional_22,<br>Knee_2D_MAPSS-echo1_Research_Compositional_39	knee_MAPSS_SAM2	1	Knee_2D_MAPSS-echo1_Research_Compositional_22,<br>Knee_2D_MAPSS-echo1_Research_Compositional_39
knee_3D_DESS_SAM	1	Knee_3D_DESS_Research_Anatomical_86	knee_3D_DESS_MedSAM	1	Knee_3D_DESS_Research_Anatomical_86	knee_3D_DESS_SAM2	1	Knee_3D_DESS_Research_Anatomical_86
knee_3D_CUBE_SAM	1	Knee_3D_CUBE_Research_Anatomical_300	knee_3D_CUBE_MedSAM	1	Knee_3D_CUBE_Research_Anatomical_300	knee_3D_CUBE_SAM2	1	Knee_3D_CUBE_Research_Anatomical_300
mixed_Knee_SAM	2	Knee_2D_MAPSS-echo1_Research_Compositional_22,<br>Knee_2D_MAPSS-echo1_Research_Compositional_39,<br>Knee_3D_DESS_Research_Anatomical_86,<br>Knee_3D_CUBE_Research_Anatomical_300	–	–	–	–	–	–
spine_T1ax_SAM	1	Spine_2D_T1ax_Clinical_Anatomical_59	spine_T1ax_MedSAM	1	Spine_2D_T1ax_Clinical_Anatomical_59	spine_T1ax_SAM2	1	Spine_2D_T1ax_Clinical_Anatomical_59
spine_T2ax_SAM	1	Spine_2D_T2ax_Clinical_Anatomical_38	spine_T2ax_MedSAM	1	Spine_2D_T2ax_Clinical_Anatomical_38	spine_T2ax_SAM2	1	Spine_2D_T2ax_Clinical_Anatomical_38
spine_T1sag_SAM	1	Spine_2D_T1sag_Clinical_Anatomical_111,<br>Spine_2D_T1sag_Clinical_Anatomical_88	spine_T1sag_MedSAM	1	Spine_2D_T1sag_Clinical_Anatomical_111,<br>Spine_2D_T1sag_Clinical_Anatomical_88	spine_T1sag_SAM2	1	Spine_2D_T1sag_Clinical_Anatomical_111,<br>Spine_2D_T1sag_Clinical_Anatomical_88
spine_mixed_axes_SAM	2	Spine_2D_T1ax_Clinical_Anatomical_59,<br>Spine_2D_T2ax_Clinical_Anatomical_38,<br>Spine_2D_T1sag_Clinical_Anatomical_111,<br>Spine_2D_T1sag_Clinical_Anatomical_88	–	–	–	–	–	–
thigh_T1ax_SAM	1	Thigh_2D_T1ax_Clinical_Anatomical_50	thigh_T1ax_MedSAM	1	Thigh_2D_T1ax_Clinical_Anatomical_50	thigh_T1ax_SAM2	1	Thigh_2D_T1ax_Clinical_Anatomical_50
mixed_Muscle_SAM	2	Thigh_2D_T1ax_Clinical_Anatomical_50,<br>Spine_2D_T1ax_Clinical_Anatomical_59	–	–	–	–	–	–
mskSAM	3	Hip_3D_CUBE_Research_Anatomical_42,<br>Shoulder_3D_CUBE_Research_Anatomical_28,<br>Knee_2D_MAPSS-echo1_Research_Compositional_22,<br>Knee_2D_MAPSS-echo1_Research_Compositional_39,<br>Knee_3D_DESS_Research_Anatomical_86,<br>Knee_3D_CUBE_Research_Anatomical_300,<br>Spine_2D_T1ax_Clinical_Anatomical_59,<br>Spine_2D_T2ax_Clinical_Anatomical_38,<br>Spine_2D_T1sag_Clinical_Anatomical_111,<br>Spine_2D_T1sag_Clinical_Anatomical_88,<br>Thigh_2D_T1ax_Clinical_Anatomical_50	msk_MedSAM	3	Hip_3D_CUBE_Research_Anatomical_42,<br>Shoulder_3D_CUBE_Research_Anatomical_28,<br>Knee_2D_MAPSS-echo1_Research_Compositional_22,<br>Knee_2D_MAPSS-echo1_Research_Compositional_39,<br>Knee_3D_DESS_Research_Anatomical_86,<br>Knee_3D_CUBE_Research_Anatomical_300,<br>Spine_2D_T1ax_Clinical_Anatomical_59,<br>Spine_2D_T2ax_Clinical_Anatomical_38,<br>Spine_2D_T1sag_Clinical_Anatomical_111,<br>Spine_2D_T1sag_Clinical_Anatomical_88,<br>Thigh_2D_T1ax_Clinical_Anatomical_50	msk_SAM2	3	Hip_3D_CUBE_Research_Anatomical_42,<br>Shoulder_3D_CUBE_Research_Anatomical_28,<br>Knee_2D_MAPSS-echo1_Research_Compositional_22,<br>Knee_2D_MAPSS-echo1_Research_Compositional_39,<br>Knee_3D_DESS_Research_Anatomical_86,<br>Knee_3D_CUBE_Research_Anatomical_300,<br>Spine_2D_T1ax_Clinical_Anatomical_59,<br>Spine_2D_T2ax_Clinical_Anatomical_38,<br>Spine_2D_T1sag_Clinical_Anatomical_111,<br>Spine_2D_T1sag_Clinical_Anatomical_88,<br>Thigh_2D_T1ax_Clinical_Anatomical_50

Table S6: Fine-tuning Experiments of SAM, MedSAM, and SAM2 Models on Musculoskeletal MRI Datasets.
This table summarizes the fine-tuning experiments conducted with the SAM, MedSAM, and SAM2 models across various musculoskeletal (MSK) MRI datasets. Each experiment lists the name, type, and the datasets used for fine-tuning. The experiments encompass anatomical regions such as the knee, shoulder, spine, hip, and thigh, with models fine-tuned on individual datasets or combinations thereof (e.g., mixed or fused datasets). The experiment type indicates whether training involved a single dataset or multiple datasets. This overview of fine-tuning strategies facilitates the evaluation of model adaptability and performance across diverse MSK MRI imaging conditions and dataset configurations.

Top 5 Mean DICE Scores Across Musculoskeletal Datasets: Finetuning Strategy, Base Models, and Experiment Parameters

Dataset	*Anatomy*	*Number of Labels*	*Experiment Name*	*Base Model*	*Experiment Type*	*Mean DICE (all labels)*	*Mean Bone DICE*	*Mean Cartilage DICE*	*Mean Muscle DICE*	*Mean Fat DICE*	*Mean Nerve DICE*	*image_encoder*	*bbox_shift*	*max_subject_set*
Knee_2D_MAPSS-echo1_Research_Compositional_39	knee	6	knee_MAPSS_SAM2	SAM2	1	0.84239	---	0.84239	---	---	---	TRUE	0	full
Knee_2D_MAPSS-echo1_Research_Compositional_39	knee	6	knee_MAPSS_SAM	SAM	1	0.83132	---	0.83132	---	---	---	TRUE	0	full
Knee_2D_MAPSS-echo1_Research_Compositional_39	knee	6	mixed_Knee_SAM	SAM	2	0.83048	---	0.83048	---	---	---	FALSE	0	full
Knee_2D_MAPSS-echo1_Research_Compositional_39	knee	6	mskSAM	SAM	3	0.82968	---	0.82968	---	---	---	FALSE	0	full
Knee_2D_MAPSS-echo1_Research_Compositional_39	knee	6	mskSAM2	SAM2	3	0.82911	---	0.82911	---	---	---	TRUE	0	full
Shoulder_3D_CUBE_Research_Anatomical_28	shoulder	1	mskSAM2	SAM2	3	0.92343	0.92343	---	---	---	---	TRUE	0	full
Shoulder_3D_CUBE_Research_Anatomical_28	shoulder	1	shoulder_CUBE_SAM2	SAM2	1	0.92302	0.92302	---	---	---	---	TRUE	0	full
Shoulder_3D_CUBE_Research_Anatomical_28	shoulder	1	shoulder_CUBE_MedSAM	MedSAM	1	0.92166	0.92166	---	---	---	---	TRUE	0	full
Shoulder_3D_CUBE_Research_Anatomical_28	shoulder	1	mskSAM	SAM	3	0.91972	0.91972	---	---	---	---	TRUE	0	full
Shoulder_3D_CUBE_Research_Anatomical_28	shoulder	1	mskSAM_MedSAM	MedSAM	3	0.91887	0.91887	---	---	---	---	TRUE	0	full
Knee_3D_undersampled_CUBE_Research_Anatomical_50	knee	6	knee_3D_CUBE_MedSAM	MedSAM	1	0.92258	0.96946	0.87570	---	---	---	TRUE	0	full
Knee_3D_undersampled_CUBE_Research_Anatomical_50	knee	6	knee_3D_CUBE_SAM	SAM	1	0.92185	0.96936	0.87433	---	---	---	TRUE	0	full
Knee_3D_undersampled_CUBE_Research_Anatomical_50	knee	6	mskSAM	SAM	3	0.92105	0.97058	0.87152	---	---	---	TRUE	0	full
Knee_3D_undersampled_CUBE_Research_Anatomical_50	knee	6	mskSAM2	SAM2	3	0.92026	0.96993	0.87059	---	---	---	TRUE	0	full
Knee_3D_undersampled_CUBE_Research_Anatomical_50	knee	6	mskSAM_MedSAM	MedSAM	3	0.92006	0.97024	0.86989	---	---	---	TRUE	0	full
Hip_3D_CUBE_Research_Anatomical_42	hip	1	mskSAM2	SAM2	3	0.97648	0.97648	---	---	---	---	TRUE	0	full
Hip_3D_CUBE_Research_Anatomical_42	hip	1	mskSAM	SAM	3	0.97634	0.97634	---	---	---	---	TRUE	0	full
Hip_3D_CUBE_Research_Anatomical_42	hip	1	mskSAM_MedSAM	MedSAM	3	0.97517	0.97517	---	---	---	---	TRUE	0	full
Hip_3D_CUBE_Research_Anatomical_42	hip	1	mskSAM	SAM	3	0.97163	0.97163	---	---	---	---	FALSE	0	full
Hip_3D_CUBE_Research_Anatomical_42	hip	1	mskSAM	SAM	3	0.96904	0.96904	---	---	---	---	TRUE	20	full
Knee_3D_DESS_Research_Anatomical_86	knee	6	knee_3D_DESS_SAM2	SAM2	1	0.93923	---	0.93923	---	---	---	TRUE	0	full
Knee_3D_DESS_Research_Anatomical_86	knee	6	mskSAM2	SAM2	3	0.93469	---	0.93469	---	---	---	TRUE	0	full
Knee_3D_DESS_Research_Anatomical_86	knee	6	knee_3D_DESS_SAM2	SAM2	1	0.92169	---	0.92169	---	---	---	TRUE	20	full
Knee_3D_DESS_Research_Anatomical_86	knee	6	mskSAM	SAM	3	0.92051	---	0.92051	---	---	---	TRUE	0	full
Knee_3D_DESS_Research_Anatomical_86	knee	6	knee_3D_DESS_SAM	SAM	1	0.92043	---	0.92043	---	---	---	TRUE	0	full
Thigh_2D_T1ax_Clinical_Anatomical_50	thigh	10	mixed_Muscle_SAM	SAM	2	0.91934	0.92758	---	0.94181	0.89803	0.90996	TRUE	0	full
Thigh_2D_T1ax_Clinical_Anatomical_50	thigh	10	thigh_T1ax_SAM	SAM	1	0.91675	0.92764	---	0.94094	0.89015	0.90828	TRUE	0	full
Thigh_2D_T1ax_Clinical_Anatomical_50	thigh	10	mskSAM_MedSAM	MedSAM	3	0.91513	0.92355	---	0.93687	0.89831	0.90179	TRUE	0	full
Thigh_2D_T1ax_Clinical_Anatomical_50	thigh	10	thigh_T1ax_MedSAM	MedSAM	1	0.91320	0.92119	---	0.93640	0.89663	0.89858	TRUE	0	full
Thigh_2D_T1ax_Clinical_Anatomical_50	thigh	10	mskSAM	SAM	3	0.91212	0.91583	---	0.93958	0.89114	0.90193	TRUE	0	full
Knee_2D_MAPSS-echo1_Research_Compositional_22	knee	6	knee_MAPSS_SAM2	SAM2	1	0.87967	---	0.87967	---	---	---	TRUE	0	full
Knee_2D_MAPSS-echo1_Research_Compositional_22	knee	6	mskSAM2	SAM2	3	0.87667	---	0.87667	---	---	---	TRUE	0	full
Knee_2D_MAPSS-echo1_Research_Compositional_22	knee	6	mixed_Knee_SAM	SAM	2	0.87450	---	0.87450	---	---	---	TRUE	0	full
Knee_2D_MAPSS-echo1_Research_Compositional_22	knee	6	mskSAM_MedSAM	MedSAM	3	0.86944	---	0.86944	---	---	---	TRUE	0	full
Knee_2D_MAPSS-echo1_Research_Compositional_22	knee	6	mskSAM	SAM	3	0.86849	---	0.86849	---	---	---	TRUE	0	full
Knee_3D_CUBE_Research_Anatomical_300	knee	6	knee_3D_CUBE_SAM	SAM	1	0.95187	0.98302	0.92073	---	---	---	TRUE	0	full
Knee_3D_CUBE_Research_Anatomical_300	knee	6	knee_3D_CUBE_SAM2	SAM2	1	0.95175	0.98159	0.92191	---	---	---	TRUE	0	full
Knee_3D_CUBE_Research_Anatomical_300	knee	6	mskSAM2	SAM2	3	0.95127	0.98081	0.92174	---	---	---	TRUE	0	full
Knee_3D_CUBE_Research_Anatomical_300	knee	6	mskSAM	SAM	3	0.95125	0.98276	0.91975	---	---	---	TRUE	0	full
Knee_3D_CUBE_Research_Anatomical_300	knee	6	mixed_Knee_SAM	SAM	2	0.94973	0.98270	0.91676	---	---	---	TRUE	0	full
Spine_2D_T1ax_Clinical_Anatomical_59	lumbar_spine	4	mskSAM	SAM	3	0.93301	---	---	0.93301	---	---	TRUE	0	full
Spine_2D_T1ax_Clinical_Anatomical_59	lumbar_spine	4	mixed_Muscle_SAM	SAM	2	0.93279	---	---	0.93279	---	---	TRUE	0	full
Spine_2D_T1ax_Clinical_Anatomical_59	lumbar_spine	4	spine_mixed_axes_SAM	SAM	2	0.93142	---	---	0.93142	---	---	TRUE	0	full
Spine_2D_T1ax_Clinical_Anatomical_59	lumbar_spine	4	mskSAM_MedSAM	MedSAM	3	0.93046	---	---	0.93046	---	---	TRUE	0	full
Spine_2D_T1ax_Clinical_Anatomical_59	lumbar_spine	4	spine_T1ax_SAM	SAM	1	0.93033	---	---	0.93033	---	---	TRUE	0	full
Spine_2D_T1sag_Clinical_Anatomical_111	lumbar_spine	2	spine_T1sag_SAM2	SAM2	1	0.96166	0.96502	0.92695	---	---	---	TRUE	0	full
Spine_2D_T1sag_Clinical_Anatomical_111	lumbar_spine	2	spine_T1sag_MedSAM	MedSAM	1	0.96162	0.96514	0.92772	---	---	---	TRUE	0	full
Spine_2D_T1sag_Clinical_Anatomical_111	lumbar_spine	2	spine_mixed_axes_SAM	SAM	2	0.96142	0.96445	0.92960	---	---	---	TRUE	0	full
Spine_2D_T1sag_Clinical_Anatomical_111	lumbar_spine	2	mskSAM_MedSAM	MedSAM	3	0.96104	0.96441	0.92685	---	---	---	TRUE	0	full
Spine_2D_T1sag_Clinical_Anatomical_111	lumbar_spine	2	mskSAM	SAM	3	0.96049	0.96369	0.92579	---	---	---	TRUE	0	full
Spine_2D_T2ax_Clinical_Anatomical_38	lumbar_spine	3	spine_T2ax_SAM2	SAM2	1	0.97236	0.96778	0.97903	---	---	0.97028	TRUE	0	full
Spine_2D_T2ax_Clinical_Anatomical_38	lumbar_spine	3	spine_mixed_axes_SAM	SAM	2	0.97165	0.96639	0.97831	---	---	0.97024	TRUE	0	full
Spine_2D_T2ax_Clinical_Anatomical_38	lumbar_spine	3	spine_T2ax_SAM	SAM	1	0.97142	0.96741	0.97678	---	---	0.97009	TRUE	0	full
Spine_2D_T2ax_Clinical_Anatomical_38	lumbar_spine	3	mskSAM	SAM	3	0.97087	0.96652	0.97716	---	---	0.96894	TRUE	0	full
Spine_2D_T2ax_Clinical_Anatomical_38	lumbar_spine	3	spine_T2ax_MedSAM	MedSAM	1	0.97084	0.96654	0.97901	---	---	0.96698	TRUE	0	full
Spine_2D_T1sag_Clinical_Anatomical_88	lumbar_spine	27	spine_T1sag_SAM2	SAM2	1	0.96370	0.96779	0.95961	---	---	---	TRUE	0	full
Spine_2D_T1sag_Clinical_Anatomical_88	lumbar_spine	27	spine_mixed_axes_SAM	SAM	2	0.96193	0.96758	0.95628	---	---	---	TRUE	0	full
Spine_2D_T1sag_Clinical_Anatomical_88	lumbar_spine	27	spine_T1sag_MedSAM	MedSAM	1	0.96133	0.96717	0.95550	---	---	---	TRUE	0	full
Spine_2D_T1sag_Clinical_Anatomical_88	lumbar_spine	27	mskSAM	SAM	3	0.96097	0.96623	0.95572	---	---	---	TRUE	0	full
Spine_2D_T1sag_Clinical_Anatomical_88	lumbar_spine	27	mskSAM2	SAM2	3	0.95993	0.96402	0.95584	---	---	---	TRUE	0	full

Table S7: Top 5 Mean DICE Scores Across Musculoskeletal Datasets: Fine-tuning Strategy, Base Models, and Experiment Parameters
This table provides an overview of the top five mean DICE scores for musculoskeletal (MSK) MRI datasets, summarizing the performance of various fine-tuning strategies across different base models (SAM, MedSAM, SAM2). For each dataset, the table lists the anatomy, number of segmentation labels, experiment name, base model used, experiment type, and mean DICE scores across all labels. Additionally, the experiment parameters such as the state of the image encoder (frozen or fine-tuned), presence of bounding box shift (bbox_shift), and the maximum subject set used in training (max_subject_set) are also included. This table summarizes the top-performing models across MSK MRI tasks, which enables comparison of fine-tuning strategies and model adaptability across different anatomical regions and segmentation complexities.

TOC

Top DICE Scores by Tissue Type Across Musculoskeletal Datasets: Overall, Bone, Cartilage, Muscle, and Fat Label Performance

Dataset	*Anatomy*	*Number of Labels*	*Experiment Name*	*Base Model*	*Experiment Type*	*Mean DICE (all labels)*	*Mean Bone Label DICE*	*Mean Cartilage Label DICE*	*Mean Muscle Label DICE*	*Mean Nerve Label DICE*	*Mean Fat Label DICE*	*image encoder*	*bbox shift*	*Model Type*
Spine_2D_T1sag_Clinical_Anatomical_88	lumbar_spine	27	spine_T1sag_SAM2	SAM2	1	**0.96370**	0.96779	0.95961	---	---	---	TRUE	0	General
Spine_2D_T2ax_Clinical_Anatomical_38	lumbar_spine	3	spine_T2ax_SAM2	SAM2	1	**0.97236**	0.96778	0.97903	---	0.97028	---	TRUE	0	General
Spine_2D_T1sag_Clinical_Anatomical_111	lumbar_spine	2	spine_T1sag_SAM2	SAM2	1	**0.96166**	0.96502	0.92695	---	---	---	TRUE	0	General
Spine_2D_T1ax_Clinical_Anatomical_59	lumbar_spine	4	mskSAM	SAM	3	**0.93301**	---	---	---	---	---	TRUE	0	General
Knee_3D_CUBE_Research_Anatomical_300	knee	6	knee_3D_CUBE_SAM	SAM	1	**0.95187**	0.98302	0.92073	---	---	---	TRUE	0	General
Knee_2D_MAPSS-echo1_Research_Compositional_22	knee	6	knee_MAPSS_SAM2	SAM2	1	**0.87967**	---	---	---	---	---	TRUE	0	General
Thigh_2D_T1ax_Clinical_Anatomical_50	thigh	10	mixed_Muscle_SAM	SAM	2	**0.91934**	0.92758	---	0.94181	0.90996	0.89803	TRUE	0	General
Knee_3D_DESS_Research_Anatomical_86	knee	6	knee_3D_DESS_SAM2	SAM2	1	**0.93923**	---	---	---	---	---	TRUE	0	General
Hip_3D_CUBE_Research_Anatomical_42	hip	1	mskSAM2	SAM2	3	**0.97648**	---	---	---	---	---	TRUE	0	General
Knee_3D_undersampled_CUBE_Research_Anatomical_50	knee	6	knee_3D_CUBE_MedSAM	MedSAM	1	**0.92258**	0.96946	0.87570	---	---	---	TRUE	0	General
Shoulder_3D_CUBE_Research_Anatomical_28	shoulder	1	mskSAM2	SAM2	3	**0.92343**	---	---	---	---	---	TRUE	0	General
Knee_2D_MAPSS-echo1_Research_Compositional_39	knee	6	knee_MAPSS_SAM2	SAM2	1	**0.84239**	---	---	---	---	---	TRUE	0	General
Thigh_2D_T1ax_Clinical_Anatomical_50	thigh	10	thigh_T1ax_SAM	SAM	1	0.91675	**0.92764**	---	0.94094	0.90828	0.89015	TRUE	0	Bone
Spine_2D_T1sag_Clinical_Anatomical_88	lumbar_spine	27	spine_T1sag_SAM2	SAM2	1	0.96370	**0.96779**	0.95961	---	---	---	TRUE	0	Bone
Spine_2D_T2ax_Clinical_Anatomical_38	lumbar_spine	3	spine_mixed_axes_SAM	SAM	2	0.97140	**0.96799**	0.97816	---	0.96804	---	TRUE	0	Bone
Spine_2D_T1sag_Clinical_Anatomical_111	lumbar_spine	2	spine_T1sag_MedSAM	MedSAM	1	0.96162	**0.96514**	0.92772	---	---	---	TRUE	0	Bone
Shoulder_3D_CUBE_Research_Anatomical_28	shoulder	1	mskSAM2	SAM2	3	0.92343	**0.92343**	---	---	---	---	TRUE	0	Bone
Knee_3D_undersampled_CUBE_Research_Anatomical_50	knee	6	mskSAM	SAM	3	0.92105	**0.97058**	0.87152	---	---	---	TRUE	0	Bone
Hip_3D_CUBE_Research_Anatomical_42	hip	1	mskSAM2	SAM2	3	0.97648	**0.97648**	---	---	---	---	TRUE	0	Bone
Knee_3D_CUBE_Research_Anatomical_300	knee	6	knee_3D_CUBE_SAM	SAM	1	0.95187	**0.98302**	0.92073	---	---	---	TRUE	0	Bone
Spine_2D_T1sag_Clinical_Anatomical_88	lumbar_spine	27	spine_T1sag_SAM2	SAM2	1	0.96370	0.96779	**0.95961**	---	---	---	TRUE	0	Cartilage
Spine_2D_T2ax_Clinical_Anatomical_38	lumbar_spine	3	spine_T2ax_SAM2	SAM2	1	0.97236	0.96778	**0.97903**	---	0.97028	---	TRUE	0	Cartilage
Spine_2D_T1sag_Clinical_Anatomical_111	lumbar_spine	2	spine_mixed_axes_SAM	SAM	2	0.96142	0.96445	**0.92960**	---	---	---	TRUE	0	Cartilage
Knee_3D_CUBE_Research_Anatomical_300	knee	6	knee_3D_CUBE_SAM2	SAM2	1	0.95175	0.98159	**0.92191**	---	---	---	TRUE	0	Cartilage
Knee_2D_MAPSS-echo1_Research_Compositional_22	knee	6	knee_MAPSS_SAM2	SAM2	1	0.87967	---	**0.87967**	---	---	---	TRUE	0	Cartilage
Knee_3D_DESS_Research_Anatomical_86	knee	6	knee_3D_DESS_SAM2	SAM2	1	0.93923	---	**0.93923**	---	---	---	TRUE	0	Cartilage
Knee_3D_undersampled_CUBE_Research_Anatomical_50	knee	6	knee_3D_CUBE_MedSAM	MedSAM	1	0.92258	0.96946	**0.87570**	---	---	---	TRUE	0	Cartilage
Knee_2D_MAPSS-echo1_Research_Compositional_39	knee	6	knee_MAPSS_SAM2	SAM2	1	0.84239		**0.84239**	---	---	---	TRUE	0	Cartilage
Thigh_2D_T1ax_Clinical_Anatomical_50	thigh	10	mixed_Muscle_SAM	SAM	2	0.91934	0.92758	---	**0.94181**	0.90996	0.89803	TRUE	0	Muscle
Spine_2D_T1ax_Clinical_Anatomical_59	lumbar_spine	4	mskSAM	SAM	3	0.93301	---	---	**0.93301**	---	---	TRUE	0	Muscle
Thigh_2D_T1ax_Clinical_Anatomical_50	thigh	10	mixed_Muscle_SAM	SAM	2	0.91934	0.92758	---	**0.94181**	**0.90996**	0.89803	TRUE	0	Nerve
Spine_2D_T2ax_Clinical_Anatomical_38	lumbar_spine	3	spine_T2ax_SAM2	SAM2	1	0.97236	0.96778	0.97903	---	**0.97028**	---	TRUE	0	Nerve
Thigh_2D_T1ax_Clinical_Anatomical_50	thigh	10	mskSAM_MedSAM	MedSAM	3	0.91513	0.92355	---	0.93687	0.90179	**0.89831**	TRUE	0	Fat

Table S8: Top DICE Scores by Tissue Type Across Musculoskeletal Datasets: Overall, Bone, Cartilage, Muscle, and Fat Label Performance. This table presents a detailed breakdown of the top DICE scores across musculoskeletal (MSK) MRI datasets, focusing on tissue-specific segmentation performance. For each dataset, the table lists the anatomy, number of segmentation labels, experiment name, base model (SAM, MedSAM, SAM2), experiment type, and the mean DICE score across all labels. Additionally, it provides DICE scores specific to key tissue types (bone, cartilage, muscle, and fat). Key parameters such as the state of the image encoder, presence of bounding box shift (bbox_shift), and the model type (e.g., general, bone, cartilage, etc.) are also included. This tissue-specific analysis allows for a more granular understanding of model performance. This is useful in determining how different fine-tuning strategies and base models influence segmentation accuracy across various tissue types and anatomical regions.

Evaluation of Mean DICE Scores for the mskSAM Finetuning Experiment Across MedSAM, SAM, and SAM2: Identifying the Optimal Musculoskeletal MRI Foundation Model			
Dataset	*Mean DICE Score*		
	mskSAM_MedSAM	*mskSAM*	*mskSAM2*
Knee_2D_MAPSS-echo1_Research_Compositional_39	0.821	0.812	0.829
Shoulder_3D_CUBE_Research_Anatomical_28	0.919	0.920	0.923
Knee_3D_undersampled_CUBE_Research_Anatomical_50	0.920	0.921	0.920
Hip_3D_CUBE_Research_Anatomical_42	0.975	0.976	0.976
Knee_3D_DESS_Research_Anatomical_86	0.915	0.921	0.935
Thigh_2D_T1ax_Clinical_Anatomical_50	0.915	0.912	0.901
Knee_2D_MAPSS-echo1_Research_Compositional_22	0.869	0.868	0.877
Knee_3D_CUBE_Research_Anatomical_300	0.949	0.951	0.951
Spine_2D_T1ax_Clinical_Anatomical_59	0.930	0.933	0.930
Spine_2D_T1sag_Clinical_Anatomical_111	0.961	0.960	0.960
Spine_2D_T2ax_Clinical_Anatomical_38	0.969	0.971	0.970
Spine_2D_T1sag_Clinical_Anatomical_88	0.959	0.961	0.960
Musculoskeletal Mean DICE:	0.925	0.926	**0.928**
Knee Set Mean DICE:	0.895	0.895	0.902
Spine Set Mean DICE:	0.955	0.956	0.955

Table S9: Evaluation of Mean DICE Scores for the mskSAM Fine-tuning Experiment Across MedSAM, SAM, and SAM2: Identifying the Optimal Musculoskeletal MRI Foundation Model

This table presents the mean DICE scores from the mskSAM fine-tuning experiment, comparing the performance of three foundation models: MedSAM, SAM, and SAM2. For each dataset, the table shows the mean DICE score achieved by mskSAM with MedSAM, SAM, and SAM2 as the baseline weights. The table concludes with the overall musculoskeletal mean DICE scores, as well as the knee and spine set mean DICE scores for each model. These results help identify the optimal baseline model for creating a robust musculoskeletal MRI-specific foundation model. The consistent use of identical parameters (image encoder and bounding box shift) across experiments ensures a direct comparison of the fine-tuning outcomes. As a result, we can better understand which baseline weights provide the strongest performance for MSK MRI tasks.

Kolmogorov-Smirnov (KS) Test for Data Imputation Sensitivity Across Datasets and Global Study

Group	*Column*	*KS Statistic*	*P-Value*
Knee_2D_MAPSS-echo1_Research_Compositional_39	Weight	0	1
Knee_2D_MAPSS-echo1_Research_Compositional_39	Time to Echo (TE)	0	1
Knee_2D_MAPSS-echo1_Research_Compositional_39	Pixel Spacing	0	1
Knee_2D_MAPSS-echo1_Research_Compositional_39	Sex (0=M, 1=F)	0.06068268	0.084928942
Knee_2D_MAPSS-echo1_Research_Compositional_39	Age	0	1
Knee_2D_MAPSS-echo1_Research_Compositional_39	Flip Angle	0	1
Knee_2D_MAPSS-echo1_Research_Compositional_39	Image Row Size	0	1
Shoulder_3D_CUBE_Research_Anatomical_28	Weight	0	1
Shoulder_3D_CUBE_Research_Anatomical_28	Time to Echo (TE)	0	1
Shoulder_3D_CUBE_Research_Anatomical_28	Pixel Spacing	0	1
Shoulder_3D_CUBE_Research_Anatomical_28	Sex (0=M, 1=F)	0	1
Shoulder_3D_CUBE_Research_Anatomical_28	Age	0	1
Shoulder_3D_CUBE_Research_Anatomical_28	Flip Angle	0	1
Shoulder_3D_CUBE_Research_Anatomical_28	Image Row Size	0	1
Knee_3D_undersampled_CUBE_Research_Anatomical_50	Weight	0	1
Knee_3D_undersampled_CUBE_Research_Anatomical_50	Time to Echo (TE)	0	1
Knee_3D_undersampled_CUBE_Research_Anatomical_50	Pixel Spacing	0	1
Knee_3D_undersampled_CUBE_Research_Anatomical_50	Sex (0=M, 1=F)	0	1
Knee_3D_undersampled_CUBE_Research_Anatomical_50	Age	0	1
Knee_3D_undersampled_CUBE_Research_Anatomical_50	Flip Angle	0	1
Knee_3D_undersampled_CUBE_Research_Anatomical_50	Image Row Size	0	1
Hip_3D_CUBE_Research_Anatomical_42	Weight	0	1
Hip_3D_CUBE_Research_Anatomical_42	Time to Echo (TE)	0	1
Hip_3D_CUBE_Research_Anatomical_42	Pixel Spacing	0	1
Hip_3D_CUBE_Research_Anatomical_42	Sex (0=M, 1=F)	0	1
Hip_3D_CUBE_Research_Anatomical_42	Age	0	1
Hip_3D_CUBE_Research_Anatomical_42	Flip Angle	0	1
Hip_3D_CUBE_Research_Anatomical_42	Image Row Size	0	1
Knee_3D_DESS_Research_Anatomical_86	Weight	0	1
Knee_3D_DESS_Research_Anatomical_86	Time to Echo (TE)	0	1
Knee_3D_DESS_Research_Anatomical_86	Pixel Spacing	0	1
Knee_3D_DESS_Research_Anatomical_86	Sex (0=M, 1=F)	0	1
Knee_3D_DESS_Research_Anatomical_86	Age	0	1
Knee_3D_DESS_Research_Anatomical_86	Flip Angle	0	1
Knee_3D_DESS_Research_Anatomical_86	Image Row Size	0	1
Thigh_2D_T1ax_Clinical_Anatomical_50	Weight	0	1
Thigh_2D_T1ax_Clinical_Anatomical_50	Time to Echo (TE)	0	1
Thigh_2D_T1ax_Clinical_Anatomical_50	Pixel Spacing	0	1
Thigh_2D_T1ax_Clinical_Anatomical_50	Sex (0=M, 1=F)	0	1
Thigh_2D_T1ax_Clinical_Anatomical_50	Age	0	1
Thigh_2D_T1ax_Clinical_Anatomical_50	Flip Angle	0	1
Thigh_2D_T1ax_Clinical_Anatomical_50	Image Row Size	0	1
Knee_2D_MAPSS-echo1_Research_Compositional_22	Weight	0	1
Knee_2D_MAPSS-echo1_Research_Compositional_22	Time to Echo (TE)	0	1
Knee_2D_MAPSS-echo1_Research_Compositional_22	Pixel Spacing	0	1
Knee_2D_MAPSS-echo1_Research_Compositional_22	Sex (0=M, 1=F)	0	1
Knee_2D_MAPSS-echo1_Research_Compositional_22	Age	0	1
Knee_2D_MAPSS-echo1_Research_Compositional_22	Flip Angle	0	1
Knee_2D_MAPSS-echo1_Research_Compositional_22	Image Row Size	0	1

Group	*Column*	*KS Statistic*	*P-Value*
Knee_3D_CUBE_Research_Anatomical_300	Weight	0	1
Knee_3D_CUBE_Research_Anatomical_300	Time to Echo (TE)	0	1
Knee_3D_CUBE_Research_Anatomical_300	Pixel Spacing	0	1
Knee_3D_CUBE_Research_Anatomical_300	Sex (0=M, 1=F)	0	1
Knee_3D_CUBE_Research_Anatomical_300	Age	0	1
Knee_3D_CUBE_Research_Anatomical_300	Flip Angle	0	1
Knee_3D_CUBE_Research_Anatomical_300	Image Row Size	0	1
Spine_2D_T1ax_Clinical_Anatomical_59	Weight	0	1
Spine_2D_T1ax_Clinical_Anatomical_59	Time to Echo (TE)	0	1
Spine_2D_T1ax_Clinical_Anatomical_59	Pixel Spacing	0	1
Spine_2D_T1ax_Clinical_Anatomical_59	Sex (0=M, 1=F)	0	1
Spine_2D_T1ax_Clinical_Anatomical_59	Age	0	1
Spine_2D_T1ax_Clinical_Anatomical_59	Flip Angle	0	1
Spine_2D_T1ax_Clinical_Anatomical_59	Image Row Size	0	1
Spine_2D_T1sag_Clinical_Anatomical_111	Weight	0	1
Spine_2D_T1sag_Clinical_Anatomical_111	Time to Echo (TE)	0	1
Spine_2D_T1sag_Clinical_Anatomical_111	Pixel Spacing	0	1
Spine_2D_T1sag_Clinical_Anatomical_111	Sex (0=M, 1=F)	0	1
Spine_2D_T1sag_Clinical_Anatomical_111	Age	0	1
Spine_2D_T1sag_Clinical_Anatomical_111	Flip Angle	0	1
Spine_2D_T1sag_Clinical_Anatomical_111	Image Row Size	0	1
Spine_2D_T2ax_Clinical_Anatomical_38	Weight	0	1
Spine_2D_T2ax_Clinical_Anatomical_38	Time to Echo (TE)	0	1
Spine_2D_T2ax_Clinical_Anatomical_38	Pixel Spacing	0	1
Spine_2D_T2ax_Clinical_Anatomical_38	Sex (0=M, 1=F)	0	1
Spine_2D_T2ax_Clinical_Anatomical_38	Age	0	1
Spine_2D_T2ax_Clinical_Anatomical_38	Flip Angle	0	1
Spine_2D_T2ax_Clinical_Anatomical_38	Image Row Size	0	1
Spine_2D_T1sag_Clinical_Anatomical_88	Weight	0	1
Spine_2D_T1sag_Clinical_Anatomical_88	Time to Echo (TE)	0	1
Spine_2D_T1sag_Clinical_Anatomical_88	Pixel Spacing	0	1
Spine_2D_T1sag_Clinical_Anatomical_88	Sex (0=M, 1=F)	0.023809524	0.710990293
Spine_2D_T1sag_Clinical_Anatomical_88	Age	0.483333333	3.94E-79
Spine_2D_T1sag_Clinical_Anatomical_88	Flip Angle	0	1
Spine_2D_T1sag_Clinical_Anatomical_88	Image Row Size	0	1

Global Dataset Level		
Column	*KS Statistic*	*P-Value*
Weight	0	1
Time to Echo (TE)	0	1
Pixel Spacing	0	1
Sex (0=M, 1=F)	0.006566855	0.855921346
Age	0.065668732	3.57834E-31
Flip Angle	0	1
Image Row Size	0	1

Table S10: Kolmogorov-Smirnov (KS) Test for Data Imputation Sensitivity Across Datasets and Global Study. This table shows the results of the two-sided Kolmogorov-Smirnov (KS) test, comparing the distributions of original and imputed data for each dataset and the overall study. The KS statistic measures the maximum difference between the cumulative distributions of the two samples, with the p-value indicating whether the observed differences are statistically significant. The null hypothesis posits no difference between the distributions.

Variance Inflation Factor (VIF) Analysis Before and After Multicollinearity Adjustment for MRI Imaging Features		
feature	*Initial VIF*	
const	73.057689	22.99147
Time to Echo (TE)	6.377675	1.255188
Flip Angle	8.843774	8.703929
Echo Train Length (ETL)	53.479301	Removed
Field Strength	6.40147	1.457491
Scanner Vendor (default Siemens)	22.819464	3.413884
Slice Thickness	5.770755	5.067927
Image Row Size	4.158205	3.869231
Specific Absorption Rate (SAR)	17.231888	Removed
Pixel Spacing	5.167468	5.163628
Acquisition Mode (default 3D)	26.310678	2.167837

Table S11: Variance Inflation Factor (VIF) Analysis Before and After Multicollinearity Adjustment for MRI Imaging Features. This table presents the VIF analysis conducted to identify and mitigate multicollinearity among MRI imaging features prior to hierarchical mixed-effects modeling. The first section (Initial VIF) displays the initial VIF values, highlighting several features with VIF values exceeding 10, indicating high multicollinearity (e.g., “Echo Train Length (ETL)”, “SAR”). In the second section (Post-VIF Check), features with high collinearity were removed, and the remaining features were re-assessed, confirming that all variables had acceptable VIF values (<10), ensuring multicollinearity was adequately addressed before proceeding with further analysis.

Effect of MRI Imaging and Experiment Parameters on Model Performance: Type 1 (Single Dataset Finetuning)

	Coef.	*Std.Err.*	*z*	*P>|z|*	*[0.025*	*0.975]*	*Effect Size [95% CI]*
Intercept	0.384	0.052	7.453	0	0.283	0.483	0.384 [0.283, 0.483]
C(ExperimentType, Treatment(reference=0))[T.1.0]	0.441	0.025	17.532	0	0.392	0.491	0.441 [0.392, 0.491]
TE	-0.066	0.028	-2.394	0.017	-0.121	-0.013	-0.066 [-0.121, -0.013]
PixelSpacing	-0.061	0.042	-1.452	0.146	-0.143	0.02	-0.061 [-0.143, 0.02]
SliceThickness	0.05	0.047	1.07	0.285	-0.042	0.141	0.05 [-0.042, 0.141]
FlipAngle	0.714	0.101	7.074	0	0.516	0.909	0.714 [0.516, 0.909]
AcquisitionMode	0.027	0.132	0.204	0.838	-0.231	0.284	0.027 [-0.231, 0.284]
TE:PixelSpacing	0.063	0.06	1.045	0.296	-0.055	0.178	0.063 [-0.055, 0.178]
TE:SliceThickness	0.116	0.041	2.805	0.005	0.035	0.197	0.116 [0.035, 0.197]
TE:ExperimentType	0.018	0.01	1.704	0.088	-0.003	0.039	0.018 [-0.003, 0.039]
PixelSpacing:ExperimentType	0.083	0.015	5.482	0	0.053	0.113	0.083 [0.053, 0.113]
FlipAngle:ExperimentType	-0.585	0.048	-12.145	0	-0.679	-0.498	-0.585 [-0.679, -0.498]
AcquisitionMode:ExperimentType	0.216	0.029	7.463	0	0.159	0.278	0.216 [0.159, 0.278]
TE:ImageEncoderFinetuned	-0.007	0.007	-0.999	0.318	-0.02	0.006	-0.007 [-0.02, 0.006]
TE:BoundingBoxShiftApplied	0.007	0.007	0.985	0.324	-0.007	0.02	0.007 [-0.007, 0.02]
FlipAngle:ImageEncoderFinetuned	0.03	0.015	1.998	0.046	0.001	0.06	0.03 [0.001, 0.06]
FlipAngle:BoundingBoxShiftApplied	-0.044	0.015	-2.96	0.003	-0.073	-0.001	-0.044 [-0.073, -0.001]
AcquisitionMode:ImageEncoderFinetuned	0.018	0.02	0.874	0.382	-0.022	0.057	0.018 [-0.022, 0.057]
AcquisitionMode:BoundingBoxShiftApplied	-0.001	0.02	-0.036	0.971	-0.041	0.03	-0.001 [-0.041, 0.03]
Group Var	0.002	0.037					

Effect of MRI Imaging and Experiment Parameters on Model Performance: Type 2 (Mixed Dataset Finetuning by Anatomy, Sequence, or Label Type)

	Coef.	*Std.Err.*	*z*	*P>|z|*	*[0.025*	*0.975]*	*Effect Size [95% CI]*
Intercept	0.36	0.047	7.732	0	0.269	0.451	0.36 [0.269, 0.451]
C(ExperimentType, Treatment(reference=0))[T.2.0]	0.438	0.042	10.463	0	0.356	0.52	0.438 [0.356, 0.52]
TE	-0.099	0.03	-3.27	0.001	-0.158	-0.04	-0.099 [-0.158, -0.04]
PixelSpacing	-0.052	0.035	-1.467	0.142	-0.122	0.017	-0.052 [-0.122, 0.017]
SliceThickness	0.059	0.038	1.542	0.123	-0.016	0.134	0.059 [-0.016, 0.134]
FlipAngle	0.726	0.093	7.765	0	0.543	0.909	0.726 [0.543, 0.909]
AcquisitionMode	0.115	0.121	0.95	0.342	-0.123	0.353	0.115 [-0.123, 0.353]
TE:PixelSpacing	0.038	0.052	0.744	0.457	-0.063	0.139	0.038 [-0.063, 0.139]
TE:SliceThickness	0.155	0.036	4.352	0	0.085	0.225	0.155 [0.085, 0.225]
TE:ExperimentType	0.003	0.01	0.281	0.778	-0.017	0.023	0.003 [-0.017, 0.023]
PixelSpacing:ExperimentType	0.034	0.013	2.63	0.009	0.009	0.059	0.034 [0.009, 0.059]
FlipAngle:ExperimentType	-0.282	0.041	-6.864	0	-0.362	-0.201	-0.282 [-0.362, -0.201]
AcquisitionMode:ExperimentType	0.075	0.03	2.531	0.011	0.017	0.134	0.075 [0.017, 0.134]
TE:ImageEncoderFinetuned	-0.003	0.017	-0.163	0.87	-0.036	0.03	-0.003 [-0.036, 0.03]
TE:BoundingBoxShiftApplied	0.007	0.017	0.401	0.689	-0.026	0.04	0.007 [-0.026, 0.04]
FlipAngle:ImageEncoderFinetuned	0.018	0.035	0.508	0.611	-0.05	0.085	0.018 [-0.05, 0.085]
FlipAngle:BoundingBoxShiftApplied	-0.045	0.035	-1.294	0.196	-0.112	0.023	-0.045 [-0.112, 0.023]
AcquisitionMode:ImageEncoderFinetuned	0.003	0.051	0.062	0.95	-0.097	0.103	0.003 [-0.097, 0.103]
AcquisitionMode:BoundingBoxShiftApplied	-0.009	0.051	-0.168	0.867	-0.108	0.091	-0.009 [-0.108, 0.091]
Group Var	0.001	0.015					

Effect of MRI Imaging and Experiment Parameters on Model Performance: Type 3 (Complete Musculoskeletal MRI Dataset Combination)

	Coef.	*Std.Err.*	*z*	*P>|z|*	*[0.025*	*0.975]*	*Effect Size [95% CI]*
Intercept	0.385	0.037	10.282	0	0.311	0.458	0.385 [0.311, 0.458]
C(ExperimentType, Treatment(reference=0))[T.3.0]	0.458	0.029	15.853	0	0.402	0.515	0.458 [0.402, 0.515]
TE	-0.058	0.02	-2.91	0.004	-0.096	-0.019	-0.058 [-0.096, -0.019]
PixelSpacing	-0.08	0.028	-2.833	0.005	-0.136	-0.025	-0.08 [-0.136, -0.025]
SliceThickness	0.006	0.028	0.197	0.844	-0.05	0.061	0.006 [-0.05, 0.061]
FlipAngle	0.773	0.074	10.485	0	0.628	0.917	0.773 [0.628, 0.917]
AcquisitionMode	-0.093	0.083	-1.118	0.264	-0.256	0.07	-0.093 [-0.256, 0.07]
TE:PixelSpacing	0.041	0.039	1.05	0.294	-0.035	0.117	0.041 [-0.035, 0.117]
TE:SliceThickness	0.074	0.024	3.02	0.003	0.026	0.122	0.074 [0.026, 0.122]
TE:ExperimentType	0.01	0.004	2.544	0.011	0.002	0.017	0.01 [0.002, 0.017]
PixelSpacing:ExperimentType	0.034	0.005	6.137	0	0.023	0.044	0.034 [0.023, 0.044]
FlipAngle:ExperimentType	-0.209	0.018	-11.456	0	-0.245	-0.173	-0.209 [-0.245, -0.173]
AcquisitionMode:ExperimentType	0.062	0.011	5.835	0	0.041	0.082	0.062 [0.041, 0.082]
TE:ImageEncoderFinetuned	0	0.009	-0.024	0.981	-0.018	0.017	0 [-0.018, 0.017]
TE:BoundingBoxShiftApplied	0.008	0.009	0.914	0.361	-0.009	0.025	0.008 [-0.009, 0.025]
FlipAngle:ImageEncoderFinetuned	0.023	0.02	1.136	0.256	-0.016	0.062	0.023 [-0.016, 0.062]
FlipAngle:BoundingBoxShiftApplied	-0.055	0.02	-2.745	0.006	-0.094	-0.016	-0.055 [-0.094, -0.016]
AcquisitionMode:ImageEncoderFinetuned	0.009	0.022	0.419	0.675	-0.034	0.052	0.009 [-0.034, 0.052]
AcquisitionMode:BoundingBoxShiftApplied	0.002	0.022	0.113	0.91	-0.04	0.045	0.002 [-0.04, 0.045]
Group Var	0.001	0.012					

Table S12: Effect of MRI Imaging and Experimental Parameters on Dataset-level Dice Score Across Different Fine-tuning Strategies This set of tables summarizes the results of a hierarchical mixed-effects analysis investigating the impact of MRI imaging parameters and experimental design factors on dataset-level Dice scores. The analysis includes three different fine-tuning strategies relative to the SAM baseline model (no fine-tuning): Type 1: Single dataset fine-tuning. Type 2: Mixed dataset fine-tuning based on anatomy, tissue type, or label similarity. Type 3: Comprehensive fine-tuning using all musculoskeletal MRI datasets (mskSAM). The response variable, MeanDiceScore, represents the average segmentation performance across all labels and subjects within each dataset. The predictors in the model include both MRI imaging parameters and experimental conditions. The MRI parameters are Echo Time (TE), Pixel Spacing (PixelSpacing), Slice Thickness (SliceThickness), Flip Angle (FlipAngle), and Acquisition Mode (AcquisitionMode) ($0 = 2D$, $1 = 3D$). The experimental conditions include Image Encoder Fine-tuned (ImageEncoderFine-tuned) and Bounding Box Shift Applied (BoundingBoxShiftApplied), both encoded as binary variables ($0 =$ not applied, $1 =$ applied). The interaction terms capture the combined influence of the MRI imaging parameters and experimental conditions across different fine-tuning strategies (ExperimentType, with the SAM baseline model as the reference). The tables present the estimated coefficients (Coef.), standard errors (Std.Err.), z-scores, p-values (P>|z|), and 95% confidence intervals for each parameter, alongside the computed effect sizes with their corresponding 95% confidence intervals.

Shapiro-Wilk and Levene's Test Results for Evaluating Normality and Heterogeneity in Subject-Level Biomarker Distributions Across Segmentation Labels

Dataset / Biomarker	*Label Class*	*Shapiro-Wilk Test*				*Levene's Test*	
		W Statistic Manual	*P-Value Manual Annotation*	*W Statistic Automatic*	*P-Value Automatic*	*F Statistic*	*P-Value*
Knee_2D_MAPSS-echo1_Research_Compositional_39							
Mean T1Rho Mapping							
	medial femoral cartilage	5.28E-01	3.64E-05	5.14E-01	2.47E-05	3.81E-03	9.52E-01
	lateral femoral cartilage	5.85E-01	1.83E-04	6.29E-01	5.98E-04	1.12E-05	9.97E-01
	lateral tibial cartilage	6.08E-01	3.35E-04	5.54E-01	7.59E-05	4.90E-03	9.45E-01
	medial tibial cartilage	5.85E-01	1.83E-04	6.43E-01	8.55E-04	1.98E-02	8.90E-01
	trochlear cartilage	6.23E-01	5.11E-04	6.58E-01	1.28E-03	4.18E-03	9.50E-01
	patellar cartilage	6.01E-01	2.78E-04	5.92E-01	2.20E-04	4.14E-03	9.50E-01
Mean T2 Mapping							
	medial femoral cartilage	9.81E-01	9.65E-01	8.19E-01	6.26E-02	2.34E-01	6.38E-01
	lateral femoral cartilage	9.65E-01	8.61E-01	9.75E-01	9.33E-01	1.95E-01	6.66E-01
	lateral tibial cartilage	9.52E-01	7.52E-01	9.11E-01	4.02E-01	1.24E-02	9.13E-01
	medial tibial cartilage	9.16E-01	4.38E-01	9.38E-01	6.18E-01	1.04E-01	7.53E-01
	trochlear cartilage	8.56E-01	1.39E-01	9.07E-01	3.76E-01	1.52E+00	2.41E-01
	patellar cartilage	7.55E-01	1.43E-02	7.45E-01	1.13E-02	5.86E-01	4.59E-01
Knee_3D_undersampled_CUBE_Research_Anatomical_50							
Total Bone Volume							
	femur	8.87E-01	2.20E-01	8.89E-01	2.30E-01	4.04E-04	9.84E-01
	tibia	9.43E-01	6.36E-01	9.42E-01	6.30E-01	2.28E-04	9.88E-01
	patella	8.98E-01	2.76E-01	8.96E-01	2.67E-01	1.58E-03	9.69E-01
Mean Cartilage Thickness							
	femoral cartilage	9.34E-01	5.57E-01	9.58E-01	7.95E-01	6.10E-01	4.48E-01
	tibial cartilage	9.14E-01	3.81E-01	9.53E-01	7.43E-01	1.32E-02	9.10E-01
	patellar cartilage	9.67E-01	8.76E-01	9.10E-01	3.53E-01	8.72E-02	7.72E-01
Hip_3D_CUBE_Research_Anatomical_42							
Total Bone Volume							
	femur	7.50E-01	1.27E-02	7.51E-01	1.30E-02	1.67E-05	9.97E-01
Knee_3D_DESS_Research_Anatomical_86							
Mean Cartilage Thickness							
	femoral cartilage	8.84E-01	6.56E-02	9.13E-01	1.75E-01	1.56E-02	9.01E-01
	lateral tibial cartilage	9.75E-01	9.34E-01	9.80E-01	9.77E-01	8.38E-03	9.28E-01
	medial tibial cartilage	9.35E-01	3.56E-01	9.59E-01	7.11E-01	2.13E-01	6.48E-01
	patellar cartilage	9.41E-01	4.29E-01	9.44E-01	4.71E-01	9.42E-04	9.76E-01
Knee_2D_MAPSS-echo1_Research_Compositional_22							
Mean T1Rho Mapping							
	medial femoral	9.23E-01	5.49E-01	8.95E-01	3.81E-01	2.90E-01	6.05E-01
	lateral femoral	9.42E-01	6.79E-01	9.40E-01	6.67E-01	6.30E-02	8.08E-01
	lateral tibial	9.34E-01	6.27E-01	9.49E-01	7.33E-01	3.78E-02	8.51E-01
	medial tibial	8.48E-01	1.88E-01	9.49E-01	7.27E-01	2.11E-02	8.88E-01
	trochlear	9.74E-01	8.98E-01	7.41E-01	2.44E-02	2.35E-01	6.41E-01
	patellar	9.31E-01	6.05E-01	9.34E-01	6.26E-01	3.41E-02	8.58E-01
Mean T2 Mapping							
	medial femoral	9.62E-01	8.19E-01	8.83E-01	3.25E-01	7.73E-02	7.88E-01
	lateral femoral	9.47E-01	7.14E-01	9.26E-01	5.67E-01	7.08E-06	9.98E-01
	lateral tibial	8.77E-01	2.94E-01	9.08E-01	4.57E-01	6.32E-02	8.08E-01
	medial tibial	8.93E-01	3.71E-01	9.09E-01	4.61E-01	2.18E-01	6.53E-01
	trochlear	9.83E-01	9.51E-01	9.63E-01	8.26E-01	3.55E-02	8.55E-01
	patellar	8.78E-01	3.00E-01	9.42E-01	6.82E-01	7.22E-02	7.95E-01
Knee_3D_CUBE_Research_Anatomical_300							
Total Bone Volume							
	femur	9.73E-01	3.84E-01	9.76E-01	4.65E-01	1.48E-04	9.90E-01
	tibia	9.77E-01	5.12E-01	9.77E-01	5.01E-01	1.04E-03	9.74E-01
	patella	9.60E-01	1.22E-01	9.60E-01	1.23E-01	1.50E-02	9.03E-01
Mean Cartilage Thickness							
	femoral cartilage	9.82E-01	6.99E-01	9.82E-01	6.90E-01	1.93E-01	6.61E-01
	tibial cartilage	9.66E-01	2.09E-01	9.49E-01	4.81E-02	5.47E-01	4.61E-01
	patellar cartilage	9.67E-01	2.15E-01	9.70E-01	2.86E-01	2.17E-01	6.42E-01
Thigh_2D_T1ax_Clinical_Anatomical_50							
Total Muscle Volume							
	extensors	9.41E-01	5.87E-01	9.47E-01	6.54E-01	3.19E-06	9.99E-01
	hamstrings	9.16E-01	3.58E-01	9.15E-01	3.50E-01	1.06E-02	9.19E-01
	adductors	8.92E-01	2.09E-01	8.92E-01	2.09E-01	5.50E-02	8.18E-01
	sartorius	9.55E-01	7.48E-01	9.38E-01	5.56E-01	1.56E-02	9.02E-01
	gracilis	9.01E-01	2.57E-01	9.12E-01	3.27E-01	3.50E-03	9.54E-01
Spine_2D_T1ax_Clinical_Anatomical_59							
Total Muscle Volume							
	muscle_erector_spinae	8.23E-01	2.78E-02	8.15E-01	2.20E-02	1.19E-03	9.73E-01
	muscle_multifidus	7.29E-01	1.99E-03	7.35E-01	2.35E-03	2.13E-06	9.99E-01
	muscle_psoas	7.12E-01	1.22E-03	7.30E-01	2.04E-03	4.76E-05	9.95E-01
	muscle_quadratus_lumborum	8.34E-01	3.76E-02	8.29E-01	3.25E-02	9.08E-03	9.25E-01
	total	7.79E-01	7.98E-03	7.81E-01	8.46E-03	1.80E-04	9.89E-01
Spine_2D_T1sag_Clinical_Anatomical_88							
Max Disc Height							
	L1-L2	8.72E-01	5.57E-02	8.69E-01	5.06E-02	1.78E-02	8.95E-01
	L2-L3	9.57E-01	6.74E-01	9.75E-01	9.36E-01	1.13E-01	7.40E-01
	L3-L4	9.43E-01	4.17E-01	9.28E-01	2.59E-01	7.36E-03	9.32E-01
	L4-L5	9.66E-01	8.44E-01	9.31E-01	3.49E-01	5.59E-01	4.62E-01
	L5-S1	9.42E-01	5.23E-01	8.76E-01	7.75E-02	7.06E-01	4.10E-01
	T11-T12	9.25E-01	5.06E-01	8.72E-01	1.92E-01	4.22E-03	9.49E-01
	T12-L1	9.15E-01	3.51E-01	9.63E-01	8.30E-01	2.65E-01	6.14E-01

Table S13: Shapiro-Wilk and Levene's Test Results for Evaluating Normality and Homogeneity in Subject-Level Biomarker Distributions Across Segmentation Labels. This table presents the results of the Shapiro-Wilk test for assessing normality and the Levene test for evaluating homogeneity of variances in subject-level biomarker distributions across various segmentation labels. These biomarkers are derived from both manual annotations and automatic model inferences across datasets. Metrics evaluated include mean cartilage thickness, total bone volume, total muscle volume, and disc height, among others. The Shapiro-Wilk test, a two-sided test for normality, was applied to both manually annotated and automatically generated biomarker distributions to evaluate whether the data follow a normal distribution. For each biomarker distribution, the table reports the Shapiro-Wilk W statistic and the associated p-value for both methods. The W statistic measures how well the data conform to a normal distribution, and smaller p-values indicate significant deviations from normality, suggesting non-parametric methods should be used if $p < 0.05$. Levene's test, also two-sided, was used to assess the homogeneity of variances between the biomarker distributions from manual and automatic segmentations. The median was used as the center to compare variances, making the test robust for skewed distributions. For each segmentation label, the table reports the Levene F statistic and the associated p-value. A $p < 0.05$ indicates a significant difference of the variances between the two methods differ significantly, suggesting that homogeneity assumptions for parametric testing may not hold. These tests guide the determination of whether parametric or non-parametric methods should be applied in subsequent analyses based on whether normality and homogeneity assumptions are met.

Intraclass Correlation Coefficient (ICC) Analysis for Biomarker Metrics Comparing Manual and Automated Label-Based Methods Across MRI Datasets

Dataset / Biomarker	*Label Class*	*ICC3*					*ICC - mixed model, bootstrap N=10,000*				
		ICC	*F*	*df*	*P-Value*	*CI 95%*	*Median ICC*	*df*	*CI 95%*	*CI_95%_Lower*	*CI_95%_Upper*
Knee_2D_MAPSS-echo1_Research_Compositional_39											
Mean T1Rho Mapping	medial femoral cartilage	0.997336294	749.8335768	6	2.36E-08	[0.9846, 0.9995]	0.997429121	7	[0.000 1.000]	0.00011805	0.99954082
	lateral femoral cartilage	0.996232966	529.9217364	6	6.66E-08	[0.9783, 0.9994]	0.988337388	7	[0.348 0.997]	0.34821289	0.996723536
	lateral tibial cartilage	0.993573796	310.2257282	6	3.30E-07	[0.9632, 0.9989]	0.993175851	7	[0.231 0.999]	0.231157691	0.999189134
	medial tibial cartilage	0.993355314	299.9924156	6	3.65E-07	[0.9619, 0.9989]	0.994880552	7	[0.652 0.997]	0.651616073	0.997461613
	trochlear cartilage	0.994070738	336.3100907	6	2.59E-07	[0.966, 0.999]	0.992069264	7	[0.664 0.999]	0.663700682	0.999411992
	patellar cartilage	0.992140057	253.454777	6	6.03E-07	[0.9551, 0.9986]	0.979976305	7	[0.008 0.992]	0.007740485	0.991824647
Mean T2 Mapping											
	medial femoral cartilage	0.844834988	11.88950373	6	4.14E-03	[0.3427, 0.9715]	0.857664948	7	[0.638, 0.949]	0.637796344	0.948585194
	lateral femoral cartilage	0.934661806	29.60996736	6	3.32E-04	[0.6715, 0.9885]	0.794354538	7	[0.394, 0.924]	0.394439447	0.924272217
	lateral tibial cartilage	0.751871406	7.060336642	6	1.57E-02	[0.0963, 0.9525]	0.759070355	7	[0.072, 0.916]	0.071766374	0.915928117
	medial tibial cartilage	0.644089808	4.619395137	6	4.24E-02	[-0.115, 0.9283]	0.606472254	7	[0.349, 0.813]	0.349471359	0.813335414
	trochlear cartilage	0.748350195	6.947552343	6	1.63E-02	[0.0883, 0.9517]	0.683410024	7	[0.462, 0.964]	0.460340902	0.963950746
	patellar cartilage	0.832665164	10.9520839	6	5.15E-03	[0.306, 0.9691]	0.505493113	7	[0.000, 0.688]	5.19E-09	0.688133124
Knee_3D_undersampled_CUBE_Research_Anatomical_50											
Total Bone Volume											
	femur	0.999913575	23140.55025	7	9.88E-15	[0.9996, 1.0]					
	tibia	0.999802584	10129.87672	7	1.78E-13	[0.999, 1.0]					
	patella	0.999579174	4751.555564	7	2.52E-12	[0.9979, 0.9999]					
Mean Cartilage Thickness											
	femoral cartilage	0.948603676	37.91328854	7	4.82E-05	[0.7672, 0.9895]					
	tibial cartilage	0.986104164	142.9280134	7	5.14E-07	[0.9325, 0.9972]					
	patellar cartilage	0.974175046	76.44447397	7	4.44E-06	[0.8773, 0.9948]					
Hip_3D_CUBE_Research_Anatomical_42											
Total Bone Volume											
	femur	0.999994409	357717.3746	6	2.18E-16	[1.0, 1.0]	0.99999316	7	[1.000, 1.000]	0.999875802	0.999996566
Knee_3D_DESS_Research_Anatomical_86											
Mean Cartilage Thickness											
	femoral cartilage	0.988999151	180.8041469	13	1.78E-12	[0.9661, 0.9965]					
	lateral tibial cartilage	0.995927177	490.0599027	13	2.83E-15	[0.9874, 0.9987]					
	medial tibial cartilage	0.958082393	46.71264707	13	9.87E-09	[0.875, 0.9863]					
	patellar cartilage	0.994414925	357.0972645	13	2.20E-14	[0.9827, 0.9982]					
Knee_2D_MAPSS-echo1_Research_Compositional_22											
Mean T1Rho Mapping											
	medial femoral	0.920776901	24.24516233	4	4.58E-03	[0.4325, 0.9914]	0.935490826	5	[0.703 0.960]	0.703111143	0.960493069
	lateral femoral	0.981800944	108.8958079	4	2.47E-04	[0.8379, 0.9981]	0.981362885	5	[0.937 1.000]	0.936884148	0.999720519
	lateral tibial	0.953857646	42.344126	4	1.57E-03	[0.6302, 0.9951]	0.959451263	5	[0.576 0.992]	0.576468055	0.992165383
	medial tibial	0.965934281	57.71004865	4	8.60E-04	[0.7146, 0.9964]	0.953580218	5	[0.792 0.996]	0.791884213	0.996223434
	trochlear	0.652888796	4.76184225	4	7.99E-02	[-0.3371, 0.9572]	0.556632733	5	[0.000 0.954]	7.67E-09	0.954393969
	patellar	0.980462483	101.3671537	4	2.84E-04	[0.8269, 0.9979]	0.961319047	5	[0.658 0.989]	0.658297727	0.989056628
Mean T2 Mapping											
	medial femoral	0.954979175	43.42388599	4	1.50E-03	[0.6378, 0.9952]					
	lateral femoral	0.982449851	112.9591471	4	2.30E-04	[0.8433, 0.9982]					
	lateral tibial	0.950874044	39.71167572	4	1.78E-03	[0.6105, 0.9948]					
	medial tibial	0.907470331	20.61468883	4	6.22E-03	[0.3643, 0.9899]					
	trochlear	0.930079832	27.60405029	4	3.58E-03	[0.4837, 0.9925]					
	patellar	0.960786537	50.00289139	4	1.14E-03	[0.6777, 0.9958]					
Knee_3D_CUBE_Research_Anatomical_300											
Total Bone Volume											
	femur	0.998915605	1843.346011	44	1.47E-60	[0.998, 0.9994]					
	tibia	0.999763231	8446.061464	44	4.30E-75	[0.9996, 0.9999]					
	patella	0.998719084	1560.382522	44	5.74E-59	[0.9977, 0.9993]					
Mean Cartilage Thickness											
	femoral cartilage	0.916924451	23.0744724	44	1.81E-19	[0.8538, 0.9535]	0.897717842	45	[0.845 0.939]	0.844599244	0.938684169
	tibial cartilage	0.910620589	21.37651801	44	8.48E-19	[0.8431, 0.9499]	0.892426435	45	[0.846 0.938]	0.845777335	0.937964017
	patellar cartilage	0.976363836	83.61610075	44	3.27E-31	[0.9574, 0.9869]	0.956611184	45	[0.935 0.974]	0.935433675	0.974297055
Thigh_2D_T1ax_Clinical_Anatomical_50											
Total Muscle Volume											
	extensors	0.999089397	2195.347233	8	1.50E-12	[0.996, 0.9998]					
	hamstrings	0.992963375	283.2271812	8	5.32E-09	[0.9692, 0.9984]					
	adductors	0.98536529	135.6614029	8	9.86E-08	[0.9367, 0.9967]					
	sartorius	0.993485584	306.0114047	8	3.91E-09	[0.9714, 0.9985]					
	gracilis	0.994161274	341.5404912	8	2.52E-09	[0.9744, 0.9987]					
Spine_2D_T1ax_Clinical_Anatomical_59											
Total Muscle Volume											
	muscle_erector_spinae	0.999332738	2996.324502	9	1.50E-14	[0.9973, 0.9998]	0.999393671	10	[0.997 1.000]	0.996835548	0.999631151
	muscle_multifidus	0.99987968	16621.27205	9	6.73E-18	[0.9995, 1.0]	0.999653658	10	[0.995 1.000]	0.995468268	0.999892071
	muscle_psoas	0.998787003	1647.808813	9	2.20E-13	[0.9951, 0.9997]	0.998669084	10	[0.989 1.000]	0.989153472	0.999567129
	muscle_quadratus_lumborum	0.996796393	623.2962867	9	1.74E-11	[0.9872, 0.9992]	0.996413831	10	[0.989 0.999]	0.988857253	0.999373889
	total	0.999459197	3697.203129	9	5.82E-15	[0.9978, 0.9999]	0.999399645	10	[0.998 1.000]	0.997901653	0.999858714
Spine_2D_T1sag_Clinical_Anatomical_88											
Max Disc Height											
	L1-L2	0.967883549	61.27338074	12	7.39E-09	[0.8985, 0.9901]					
	L2-L3	0.960181801	49.2282884	13	7.10E-09	[0.881, 0.987]					
	L3-L4	0.960866297	50.10684317	14	1.70E-09	[0.8878, 0.9867]					
	L4-L5	0.887347585	16.75372502	12	1.15E-05	[0.6728, 0.9642]					
	L5-S1	0.927163763	26.45885956	11	2.56E-06	[0.7679, 0.9785]					
	T11-T12	0.97062081	67.07539578	6	3.10E-05	[0.8403, 0.9949]					
	T12-L1	0.807702916	9.400573707	8	2.35E-03	[0.3591, 0.9531]					

Table S14: Intraclass Correlation Coefficient (ICC) Analysis for Biomarker Metrics Comparing Manual and Automated Segmentation Across MRI Datasets. This table presents the results of the Intraclass Correlation Coefficient (ICC) analysis, evaluating the consistency of biomarker metrics between manual annotations and automated segmentation methods across MRI datasets. Two ICC approaches are displayed: a parametric ICC3 model for biomarker distributions meeting normality and homogeneity assumptions, and a non-parametric ICC derived from a linear mixed-effects model with bootstrap resampling (N = 10,000) for distributions failing those assumptions.

The table includes ICC values, F-statistics, degrees of freedom (df), p-values, and 95% confidence intervals (CI) for both approaches. While the parametric ICC3 results are included for clarity and comparison, the non-parametric ICC is emphasized as it better captures complex patterns and provides a more accurate representation of agreement when distributional assumptions are not met. The mixed model incorporates subject-level variability by treating subjects as random effects, producing ICC values based on variance components.

Orange-highlighted cells indicate cases where the linear ICC was computed for non-normal distributions, showing overly optimistic performance compared to the more conservative and realistic non-parametric ICC values in the adjacent columns. Higher ICC values in the non- parametric results reflect stronger agreement between manual and automated methods; this provides a robust basis for assessing segmentation reliability across varying biomarker metrics.

Linear Regression Results for Normal and Homogeneous Datasets: Comparison of Automated and Manual Segmentation-Based Biomarker Metrics

Dataset / Biomarker	*Label Class*	*Linear Regression*					
		Intercept	*Slope*	*R-Value*	*R^2-Value*	*P-Value*	*Standard Err*
Knee_2D_MAPSS-echo1_Research_Compositional_39							
Mean T2 Mapping							
	medial femoral cartilage	2.823	0.925	0.848	0.719	1.59E-02	0.259
	lateral femoral cartilage	7.317	0.848	0.940	0.883	1.67E-03	0.138
	lateral tibial cartilage	8.336	0.681	0.756	0.572	4.93E-02	0.263
	medial tibial cartilage	6.708	0.774	0.653	0.427	1.11E-01	0.401
	trochlear cartilage	-16.186	1.544	0.873	0.762	1.03E-02	0.385
	patellar cartilage	-12.572	1.548	0.939	0.882	1.71E-03	0.254
Knee_3D_DESS_Research_Anatomical_86							
Mean Cartilage Thickness							
	femoral cartilage	0.074	0.965	0.989	0.979	2.10E-11	0.041
	lateral tibial cartilage	0.076	0.973	0.996	0.992	4.21E-14	0.024
	medial tibial cartilage	-0.194	1.116	0.968	0.937	1.49E-08	0.084
	patellar cartilage	-0.012	1.015	0.995	0.989	3.48E-13	0.031
Knee_2D_MAPSS-echo1_Research_Compositional_22							
Mean T2 Mapping							
	medial femoral	-8.026	1.237	0.981	0.962	3.18E-03	0.142
	lateral femoral	0.568	0.996	0.983	0.965	2.76E-03	0.109
	lateral tibial	-2.280	1.065	0.956	0.915	1.09E-02	0.188
	medial tibial	4.498	0.806	0.915	0.837	2.95E-02	0.205
	trochlear	0.498	1.007	0.933	0.870	2.07E-02	0.225
	patellar	-5.064	1.201	0.981	0.962	3.22E-03	0.138
Knee_3D_CUBE_Research_Anatomical_300							
Total Bone Volume							
	femur	0.582	0.998	0.999	0.998	6.40E-59	0.007
	tibia	0.536	0.997	1.000	1.000	2.77E-73	0.003
	patella	0.082	0.991	0.999	0.997	1.39E-57	0.008
Thigh_2D_T1ax_Clinical_Anatomical_50							
Total Muscle Volume							
	extensors	10.096	0.995	0.999	0.998	7.29E-11	0.016
	hamstrings	26.349	0.932	0.995	0.990	2.74E-08	0.035
	adductors	-7.133	1.131	0.994	0.987	6.69E-08	0.048
	sartorius	2.196	0.954	0.994	0.989	4.41E-08	0.038
	gracilis	0.107	0.979	0.994	0.989	4.62E-08	0.040
Spine_2D_T1sag_Clinical_Anatomical_88							
Max Disc Height							
	L1-L2	1.289	0.894	0.971	0.943	3.37E-08	0.066
	L2-L3	1.694	0.873	0.965	0.931	2.47E-08	0.069
	L3-L4	-0.265	0.998	0.962	0.925	1.14E-08	0.079
	L4-L5	3.669	0.716	0.914	0.835	1.25E-05	0.096
	L5-S1	-0.994	1.045	0.933	0.871	9.41E-06	0.127
	T11-T12	0.347	0.952	0.971	0.942	2.75E-04	0.105
	T12-L1	0.556	0.962	0.818	0.670	7.01E-03	0.255

Table S15: Linear Regression Analysis of Biomarker Metrics from Automated Segmentation Models and Manual Expert Annotations. This table presents the results of two-sided linear regression analyses comparing biomarker metrics derived from automated segmentation models with those obtained from manual expert annotations. For each segmentation label and biomarker, the table reports the intercept, slope, R-value (Pearson correlation coefficient), R^2 value (indicating the proportion of variance explained), p-value, and standard error (which reflects the variability of the slope coefficient). The analyses were conducted on datasets that met the assumptions of normality and homogeneity, with subject-level comparisons made between manual and automated methods.

Spearman's Rank Correlation Results for Non-Normal and Non-Homogeneous Datasets: Comparing Manual and Automated Segmentation-Based Biomarker Metrics			
Dataset	*Label Class*	*Spearman's Rank Correlation*	
		Spearman's ρ	*P-Value*
Knee_2D_MAPSS-echo1_Research_Compositional_39			
Mean T1Rho Mapping			
	medial femoral cartilage	4.29E-01	3.37E-01
	lateral femoral cartilage	9.64E-01	4.54E-04
	lateral tibial cartilage	6.79E-01	9.38E-02
	medial tibial cartilage	7.14E-01	7.13E-02
	trochlear cartilage	8.93E-01	6.81E-03
	patellar cartilage	8.93E-01	6.81E-03
Mean T2 Mapping			
	medial femoral cartilage	8.21E-01	2.34E-02
	lateral femoral cartilage	9.64E-01	4.54E-04
	lateral tibial cartilage	6.79E-01	9.38E-02
	medial tibial cartilage	5.36E-01	2.15E-01
	trochlear cartilage	8.21E-01	2.34E-02
	patellar cartilage	6.43E-01	1.19E-01
Hip_3D_CUBE_Research_Anatomical_42			
Total Bone Volume			
	femur	1.00E+00	0.00E+00
Knee_2D_MAPSS-echo1_Research_Compositional_22			
Mean T1Rho Mapping			
	medial femoral	9.00E-01	3.74E-02
	lateral femoral	1.00E+00	1.40E-24
	lateral tibial	9.00E-01	3.74E-02
	medial tibial	1.00E+00	1.40E-24
	trochlear	4.00E-01	5.05E-01
	patellar	1.00E+00	1.40E-24
Knee_3D_CUBE_Research_Anatomical_300			
Mean Cartilage Thickness			
	femoral cartilage	9.03E-01	2.04E-17
	tibial cartilage	9.09E-01	6.25E-18
	patellar cartilage	9.80E-01	7.81E-32
Spine_2D_T1ax_Clinical_Anatomical_59			
Total Muscle Volume			
	muscle_erector_spinae	1.00E+00	6.65E-64
	muscle_multifidus	1.00E+00	6.65E-64
	muscle_psoas	9.88E-01	9.31E-08
	muscle_quadratus_lumborum	1.00E+00	6.65E-64
	total	9.88E-01	9.31E-08
Spine_2D_T1sag_Clinical_Anatomical_88			
Max Disc Height			
	L1-L2	9.18E-01	9.91E-06
	L2-L3	9.32E-01	1.26E-06
	L3-L4	9.27E-01	6.53E-07
	L4-L5	9.07E-01	1.93E-05
	L5-S1	9.16E-01	2.84E-05
	T11-T12	8.57E-01	1.37E-02
	T12-L1	8.17E-01	7.22E-03

Table S16: Spearman's Rank Correlation Analysis for Non-Normal and Non-Homogeneous Biomarker Distributions. This table presents the results of two-sided Spearman's rank correlation, a non-parametric test used to assess the monotonic relationship between biomarker metrics derived from automated segmentation models and manual expert annotations. For each segmentation label and biomarker, the table reports the Spearman's correlation coefficient (ρ), reflecting the strength and direction of the monotonic association, and the p-value, with an alpha significance level of 0.05. Analyses were performed on datasets where normality or equal variance assumptions were violated, with higher ρ values indicating stronger correlations between methods.

9.6. Autolabel Pipeline Evaluation

Wilcoxon Signed-Rank Test with Benjamini-Hochberg FDR Correction for Comparing DICE Scores Using Ground Truth vs. Autolabel Bounding Box Prompts Across Models and Musculoskeletal Datasets				
Dataset	*Wilcoxon Rank Sum Test*			
	Model Type Compared	*U-Statistic*	*P-Value*	*P-Value (BH FDR)*
Knee_3D_DESS_Research_Anatomical_86	MedSAM	0.00E+00	1.22E-04	0.0004
Knee_3D_DESS_Research_Anatomical_86	SAM	1.00E+00	2.44E-04	0.0007
Knee_3D_DESS_Research_Anatomical_86	SAM2	5.10E+01	9.52E-01	0.9515
Knee_3D_DESS_Research_Anatomical_86	Finetuned (No Aug.)	0.00E+00	1.22E-04	0.0004
Knee_3D_DESS_Research_Anatomical_86	Finetuned (Bbox Aug.)	0.00E+00	1.22E-04	0.0004
Shoulder_3D_CUBE_Research_Anatomical_28	MedSAM	1.00E+00	2.50E-01	0.2717
Shoulder_3D_CUBE_Research_Anatomical_28	SAM	1.00E+00	2.50E-01	0.2717
Shoulder_3D_CUBE_Research_Anatomical_28	SAM2	3.00E+00	6.25E-01	0.6510
Shoulder_3D_CUBE_Research_Anatomical_28	Finetuned (No Aug.)	0.00E+00	1.25E-01	0.1488
Shoulder_3D_CUBE_Research_Anatomical_28	Finetuned (Bbox Aug.)	0.00E+00	1.25E-01	0.1488
Spine_2D_T1ax_Clinical_Anatomical_59	MedSAM	0.00E+00	1.95E-03	0.0035
Spine_2D_T1ax_Clinical_Anatomical_59	SAM	0.00E+00	1.95E-03	0.0035
Spine_2D_T1ax_Clinical_Anatomical_59	SAM2	0.00E+00	1.95E-03	0.0035
Spine_2D_T1ax_Clinical_Anatomical_59	Finetuned (No Aug.)	0.00E+00	1.95E-03	0.0035
Spine_2D_T1ax_Clinical_Anatomical_59	Finetuned (Bbox Aug.)	0.00E+00	1.95E-03	0.0035
Spine_2D_T1sag_Clinical_Anatomical_88	MedSAM	0.00E+00	6.10E-05	0.0004
Spine_2D_T1sag_Clinical_Anatomical_88	SAM	0.00E+00	6.10E-05	0.0004
Spine_2D_T1sag_Clinical_Anatomical_88	SAM2	1.00E+00	1.22E-04	0.0004
Spine_2D_T1sag_Clinical_Anatomical_88	Finetuned (No Aug.)	0.00E+00	6.10E-05	0.0004
Spine_2D_T1sag_Clinical_Anatomical_88	Finetuned (Bbox Aug.)	0.00E+00	6.10E-05	0.0004
Thigh_2D_T1ax_Clinical_Anatomical_50	MedSAM	0.00E+00	3.91E-03	0.0051
Thigh_2D_T1ax_Clinical_Anatomical_50	SAM	0.00E+00	3.91E-03	0.0051
Thigh_2D_T1ax_Clinical_Anatomical_50	SAM2	0.00E+00	3.91E-03	0.0051
Thigh_2D_T1ax_Clinical_Anatomical_50	Finetuned (No Aug.)	0.00E+00	3.91E-03	0.0051
Thigh_2D_T1ax_Clinical_Anatomical_50	Finetuned (Bbox Aug.)	0.00E+00	3.91E-03	0.0051

Table S17: Wilcoxon Signed-Rank Test with Benjamini-Hochberg FDR Correction for Comparing DICE Scores Using Ground Truth vs. Autolabel Bounding Box Prompts Across Models and Musculoskeletal Datasets. This table presents the results of the Wilcoxon signed-rank test comparing the DICE scores obtained from two methodologies of bounding box prompts (derived from ground truth segmentation masks and generated via an Autolabel pipeline) across various musculoskeletal MRI datasets. The models evaluated include baseline models (SAM, SAM2, MedSAM) and top- performing fine-tuned models for each dataset, with and without bounding box prompt shift augmentation applied during fine-tuning. The DICE scores represent the segmentation performance of each model when using bounding box prompts from the two different sources. The Wilcoxon signed-rank test assessed whether segmentation performance differed significantly between the two methods for each model and dataset. The table reports the test statistic (U), unadjusted p-values, and p-values adjusted to control for false discovery rates under multiple comparisons, using the Benjamini-Hochberg method. A corrected p-value below the significance level of $\alpha = 0.05$ indicates a statistically significant difference in DICE scores between the two bounding box prompt methodologies for the corresponding model and dataset.

Performance of the three-stage knee MRI triage pipeline

Operating point	Stage / Task	Tissue / Biomarker set	Model	AUC	AUC lo	AUC hi	sens	sens lo	sens hi	spec%	thr
	Stage A										
	Anomaly vs Healthy		LR	0.747	0.696	0.794	0.358	0.147	0.511	90	0.729
**all**		six biomarkers + demographics	XGB	0.726	0.675	0.774	0.318	0.238	0.421	90	0.920
			HGB	0.705	0.652	0.752	0.324	0.180	0.457	90	0.980
			ENS (mean)	0.747	0.697	0.793	0.358	0.252	0.501	90	0.862
			STACK	0.752	0.701	0.797	0.422	0.226	0.524	90	0.938
	Stage B										
	Cartilage/Bone vs Other		LR	0.746	0.711	0.779	0.178	0.087	0.286	85	0.853
			XGB	0.731	0.697	0.766	0.317	0.117	0.468	85	0.901
		same biomarkers	HGB	0.724	0.687	0.759	0.280	0.091	0.452	85	0.946
			ENS	0.746	0.712	0.780	0.290	0.079	0.452	85	0.871
			STACK	0.751	0.718	0.785	0.247	0.075	0.403	85	0.893
	Stage C part 1										
	Tissue abnormality (bone + cartilage)	femur	Mean ENS (LR + XGB) + Platt	0.753	0.645	0.842	0.464	0.000	0.671	90	--
**B85**		tibia		0.923	0.869	0.967	0.629	0.351	0.938	90	--
		patella		0.851	0.739	0.939	0.418	0.000	0.835	90	--
	Stage C part 2										
	Tissue × Biomarker	femur cartilage		0.704	0.573	0.822	0.261	0.074	0.455	90	--
		tibia cartilage		0.869	0.796	0.929	0.453	0.209	0.676	90	--
		patella cartilage	Mean ENS (LR + XGB) + Platt	0.814	0.673	0.925	0.349	0.000	0.775	90	--
		femur bone		0.810	0.714	0.887	0.435	0.085	0.651	90	--
		tibia bone		0.893	0.821	0.950	0.563	0.308	0.828	90	--
		patella bone		0.848	0.771	0.918	0.520	0.280	0.714	90	--
	Stage B										
	Cartilage/Bone vs Other		LR	0.746	0.711	0.779	0.131	0.038	0.228	90	0.879
			XGB	0.731	0.697	0.766	0.218	0.078	0.394	90	0.917
		same biomarkers	HGB	0.724	0.687	0.759	0.179	0.019	0.382	90	0.960
			ENS	0.746	0.712	0.780	0.167	0.028	0.399	90	0.917
			STACK	0.751	0.718	0.785	0.149	0.028	0.344	90	0.911
	Stage C part 1										
	Tissue abnormality (bone + cartilage)	femur	Mean ENS (LR + XGB) + Platt	0.813	0.676	0.921	0.534	0.000	0.806	90	--
**B90**		tibia		0.957	0.896	0.993	0.739	0.462	1.000	90	--
		patella		0.908	0.800	0.983	0.266	0.000	0.914	90	--
	Stage C part 2										
	Tissue × Biomarker	femur cartilage		0.750	0.602	0.885	0.362	0.000	0.719	90	--
		tibia cartilage		0.918	0.827	0.982	0.636	0.278	0.944	90	--
		patella cartilage	Mean ENS (LR + XGB) + Platt	0.866	0.718	0.976	0.297	0.000	0.854	90	--
		femur bone		0.780	0.640	0.896	0.368	0.000	0.722	90	--
		tibia bone		0.926	0.824	0.995	0.754	0.429	1.000	90	--
		patella bone		0.871	0.757	0.959	0.539	0.217	0.857	90	--

Table S18: Performance of the three stage knee MRI triage pipeline. Stage A screens the full cohort for any abnormality. Knees that pass Stage A enter Stage B, which distinguishes cartilage + bone findings from all other cases. Stage C is applied only to knees that pass Stage B and is divided into (i) Part 1, which flags joint-level abnormality (bone + cartilage) for femur, tibia and patella, and (ii) Part 2, which provides separate bone- versus cartilage-specific scores for each joint.

- AUC values are calculated once on the complete out-of-fold predictions.
- "AUC lo / hi" and "sens lo / hi" are the 2.5th and 97.5th percentiles from 2000 non-parametric bootstrap samples.
- "sens" is the sensitivity at the fixed specificity shown in spec%.
- thr is the probability threshold that attains the target specificity and is reported only for stages that set routing cut-offs (Stage A and Stage B). Stage C inherits the routed subset, so no new threshold is defined, and the column is marked "–".
- Operating-point labels:
  - all – Stage A evaluated at 90% specificity (screening step)
  - B85 / B90 - Stage B evaluated at 85% or 90% specificity, respectively

This format allows direct comparison of point estimates with their 95% confidence intervals at the operating points used in the triage workflow.

Panel A. Demographic breakdown by cascade branch pipeline

Operating point	*Stage / Subset*	*N*	*Male (n)*	*Female (n)*	*Female (%)*	*Age, y (mean ± SD)*	*Weight, kg (mean ± SD)*
**a90p_b85p**	Full cohort	930.0	440.0	490.0	52.7	44.8 ± 15.3	75.2 ± 16.0
	Stage A pass	358.0	161.0	197.0	55.0	58.0 ± 10.6	78.5 ± 16.3
	Stage B eval set	358.0	161.0	197.0	55.0	58.0 ± 10.6	78.5 ± 16.3
	Stage C pass	99.0	27.0	72.0	72.7	63.9 ± 10.3	83.3 ± 16.6
	Stage C p1 - femur	78.0	21.0	57.0	73.1	64.5 ± 10.1	81.4 ± 16.8
	Stage C p1 - tibia	44.0	16.0	28.0	63.6	65.3 ± 11.8	81.9 ± 15.1
	Stage C p1 - patella	88.0	23.0	65.0	73.9	64.2 ± 10.5	82.2 ± 16.3
	Stage C p2 - femur cart	77.0	21.0	56.0	72.7	64.4 ± 10.2	81.4 ± 16.9
	Stage C p2 - tibia cart	39.0	15.0	24.0	61.5	66.2 ± 11.7	80.6 ± 14.3
	Stage C p2 - patella cart	87.0	23.0	64.0	73.6	64.1 ± 10.5	82.0 ± 16.2
	Stage C p2 - femur bone	45.0	12.0	33.0	73.3	64.8 ± 10.5	80.4 ± 18.1
	Stage C p2 - tibia bone	28.0	10.0	18.0	64.3	68.4 ± 12.0	82.1 ± 14.8
	Stage C p2 - patella bone	49.0	8.0	41.0	83.7	63.8 ± 8.9	80.5 ± 16.6
**a90p_b90p**	Full cohort	930.0	440.0	490.0	52.7	44.8 ± 15.3	75.2 ± 16.0
	Stage A pass	358.0	161.0	197.0	55.0	58.0 ± 10.6	78.5 ± 16.3
	Stage B eval set	358.0	161.0	197.0	55.0	58.0 ± 10.6	78.5 ± 16.3
	Stage C pass	47.0	12.0	35.0	74.5	66.8 ± 10.9	86.2 ± 15.0
	Stage C p1 - femur	32.0	9.0	23.0	71.9	68.0 ± 11.1	85.3 ± 15.4
	Stage C p1 - tibia	19.0	7.0	12.0	63.2	70.2 ± 12.9	85.2 ± 12.9
	Stage C p1 - patella	40.0	10.0	30.0	75.0	67.3 ± 11.2	85.5 ± 14.0
	Stage C p2 - femur cart	31.0	9.0	22.0	71.0	67.8 ± 11.2	85.5 ± 15.6
	Stage C p2 - tibia cart	18.0	7.0	11.0	61.1	71.8 ± 11.2	83.6 ± 11.4
	Stage C p2 - patella cart	39.0	10.0	29.0	74.4	67.3 ± 11.3	85.0 ± 13.9
	Stage C p2 - femur bone	19.0	6.0	13.0	68.4	68.1 ± 12.0	84.8 ± 15.7
	Stage C p2 - tibia bone	15.0	6.0	9.0	60.0	71.7 ± 12.2	86.6 ± 12.9
	Stage C p2 - patella bone	21.0	4.0	17.0	81.0	65.2 ± 8.5	87.0 ± 12.9

Panel B. Knee-level cascade counts at the routing thresholds

setting	*forwarded*	*forwarded %*	*TN removed normals*	*FP forwarded normals*	*FN missed abnormals*	*TP forwarded abnormals*	*PPV*	*NPV*	*N norm*	*N abn*
**B85**	99	10.6	275	7	556	92	0.93	0.33	282	648
**B90**	47	5.1	277	5	606	42	0.89	0.31	282	648

Panel C. Tissue-level counts within the Stage-A evaluation set

setting	*cohort*	*target*	*TN removed normals*	*FP forwarded normals*	*FN missed abnormals*	*TP forwarded abnormals*	*PPV*	*NPV*	*N norm*	*N abn*
**B85**	A-pass	femur cartilage	112	22	147	77	0.78	0.43	134	224
		tibia cartilage	199	60	60	39	0.39	0.77	259	99
		patella cartilage	87	12	172	87	0.88	0.34	99	259
		femur bone	181	54	78	45	0.45	0.7	235	123
		tibia bone	194	71	65	28	0.28	0.75	265	93
		patella bone	177	50	82	49	0.49	0.68	227	131
**B90**	A-pass	femur cartilage	118	16	193	31	0.66	0.38	134	224
		tibia cartilage	230	29	81	18	0.38	0.74	259	99
		patella cartilage	91	8	220	39	0.83	0.29	99	259
		femur bone	207	28	104	19	0.4	0.67	235	123
		tibia bone	233	32	78	15	0.32	0.75	265	93
		patella bone	201	26	110	21	0.45	0.65	227	131

Footnote: forwarded % = (forwarded / 930); Normal and abnormal refer to the Stage-B target set (any bone or cartilage label at knee level). Thresholds are same reported in S18 for the stacker (B85 thr 0.893, B90 thr 0.911).

Table S19: Demographic breakdown and cascade counts for the knee triage pipeline at two operating points: a90p_b85p and a90p_b90p. Panel A lists demographics for each branch. Panel B reports knee-level confusion counts at the Stage-B thresholds. Panel C reports tissue-level counts inside the Stage-A evaluation set. "Normal" and "abnormal" refer to the Stage-B target set (any bone or cartilage label). Thresholds match those in Table S18.

48-month landmark → 96-month horizon TKR models								
Stage / Task	*Landmark (m)*	*Model*	*AUC*	*95% CI low*	*95% CI high*	*Calibration slope*	*Brier raw*	*Brier calib*
Model-selection								
	**0**	Logistic Regression	0.699	0.647	0.745	–	–	–
	**0**	Random Forest	0.694	0.648	0.741	–	–	–
	**0**	XGBoost	0.655	0.602	0.705	–	–	–
	**0**	Soft-vote ensemble	0.697	0.648	0.742	–	–	–
	**0**	Stacking ensemble	0.697	0.643	0.748	–	–	–
Stage / Task	*Landmark (m)*	*Model*	*AUC*	*95% CI low*	*95% CI high*	*Calibration slope*	*Brier raw*	*Brier calib*
Discrimination & calibration								
Cox baselines †	**0**	Cox baseline (naïve)	0.700	0.669	0.732	–	–	–
	**0**	Cox baseline (IPCW)	0.724	0.689	0.760	–	–	–
Demographics-only baseline‡ (age, sex, BMI), 48–96 month horizon	**0**	Logistic Regression	0.589	0.531	0.643	0.998	0.246	0.086
	**0**	Random Forest	0.583	0.525	0.637	0.998	0.162	0.086
	**12**	Logistic Regression	0.581	0.529	0.634	0.998	0.245	0.097
	**12**	Random Forest	0.569	0.524	0.616	0.998	0.176	0.098
	**24**	Logistic Regression	0.615	0.564	0.663	0.998	0.243	0.108
	**24**	Random Forest	0.526	0.478	0.576	0.998	0.190	0.110
	**36**	Logistic Regression	0.619	0.568	0.668	0.997	0.243	0.113
	**36**	Random Forest	0.531	0.495	0.567	0.998	0.195	0.115
	**48**	Logistic Regression	0.596	0.547	0.642	0.997	0.246	0.115
	**48**	Random Forest	0.585	0.539	0.630	0.997	0.191	0.115
Biomarker models (cartilage and meniscus trajectories + age, sex, BMI), 48–96 month horizon	**0**	Logistic Regression	0.704	0.652	0.753	0.998	0.216	0.080
	**0**	Random Forest	0.708	0.657	0.758	0.998	0.120	0.081
	**12**	Logistic Regression	0.720	0.675	0.764	0.997	0.206	0.091
	**12**	Random Forest	0.754	0.712	0.797	0.997	0.127	0.090
	**24**	Logistic Regression	0.727	0.682	0.769	0.997	0.209	0.101
	**24**	Random Forest	0.752	0.717	0.790	0.997	0.132	0.099
	**36**	Logistic Regression	0.712	0.668	0.755	0.997	0.214	0.108
	**36**	Random Forest	0.727	0.686	0.768	0.997	0.139	0.105
	**48**	Logistic Regression	0.705	0.659	0.749	0.997	0.212	0.107
	**48**	Random Forest	0.760	0.716	0.802	0.997	0.132	0.099
Stage / Task	*Threshold %*	*Net-benefit RF §*	*Net-benefit LR §*	*Treat-all*	*Treat-none*			
Decision-curve summary								
	Standard FP penalty	4 – 50%	5 – 39%	baseline	0	–	–	–
	MRI triage (FP 0.20)	11 – 50%	11 – 44%	negative baseline	0	–	–	–
	Surgery triage (FP 1.00)	42 – 50%	–	negative baseline	0	–	–	–
Stage / Task	*Landmark (m)*	*At-risk N*	*Events ≤ horizon*	*Event %*	*Age mean*	*Female %*	*BMI mean*	
Landmark demographics								
	**0**	1095	105	9.6	61.6	63.4	29.2	_
	**12**	1082	120	11.1	61.5	63.2	29.3	_
	**24**	1061	134	12.6	61.5	63.1	29.3	–
	**36**	1031	138	13.4	61.4	62.9	29.2	–
	**48**	994	134	13.5	61.3	63.3	29.2	–

† Cox rows use month-0 features only and the same 5-fold grouped CV. The AUC column lists C-index values (Harrell for the naive row; IPCW when indicated). 95% intervals are calculated as mean ± 1.96 × (SD/√5), where SD is the fold standard deviation with ddof=0.

‡ Inputs are age, sex, BMI only; same 5-fold grouped CV and 1,000-draw bootstrap; probabilities isotonic-calibrated, which yields the calibration slope and Brier columns on the same scale as the biomarker models.

§ Positive net-benefit means the model curve remains above both baselines across the entire threshold range and by at least 0.002.

Table S20: Performance of knee-replacement risk models trained on biomarkers up to 48 months and tested on the 48-96 month horizon. Columns report discrimination (AUC or C-index with 95% confidence intervals), calibration slope, raw and isotonic-calibrated Brier scores, decision-curve net-benefit ranges, and demographic counts for knees still at risk at each landmark. Model-selection AUCs are limited to the 0-month landmark. Cox proportional-hazards baselines use month-0 features only†. Demographics-only baselines (age, sex, BMI) are listed at each landmark‡. Net-benefit windows are shown when the model curve exceeds both “treat-all” and “treat-none” by at least 0.002§.

48-month landmark → 120-month horizon TKR models

Stage / Task	*Landmark (m)*	*Model*	*AUC*	*95% CI low*	*95% CI high*			
Model-selection								
	**0**	Logistic Regression	0.725	0.687	0.763	–	–	–
	**0**	Random Forest	0.714	0.674	0.750	–	–	–
	**0**	XGBoost	0.651	0.607	0.693	–	–	–
	**0**	Soft-vote ensemble	0.713	0.672	0.747	–	–	–
	**0**	Stacking ensemble	0.722	0.682	0.762	–	–	–
Stage / Task	*Landmark (m)*	*Model*	*AUC*	*95% CI low*	*95% CI high*	*Calibration slope*	*Brier raw*	*Brier calib*
Discrimination & calibration								
	**0**	Cox baseline (naïve)	0.712 †	0.676 ‡	0.747 ‡	–	–	–
	**0**	Cox baseline (IPCW)	0.719 †	0.681 ‡	0.756 ‡	–	–	–
	**0**	Logistic Regression	0.729	0.691	0.768	0.997	0.213	0.119
	**0**	Random Forest	0.724	0.686	0.760	0.997	0.156	0.120
	**12**	Logistic Regression	0.727	0.685	0.764	0.997	0.213	0.128
	**12**	Random Forest	0.733	0.695	0.772	0.997	0.161	0.126
	**24**	Logistic Regression	0.728	0.688	0.764	0.997	0.211	0.137
	**24**	Random Forest	0.758	0.721	0.795	0.997	0.161	0.132
	**36**	Logistic Regression	0.712	0.670	0.755	0.997	0.215	0.126
	**36**	Random Forest	0.744	0.706	0.785	0.997	0.151	0.121
	**48**	Logistic Regression	0.655	0.623	0.690	0.991	0.236	0.217
	**48**	Random Forest	0.670	0.635	0.704	0.992	0.223	0.214
Stage / Task	*Threshold %*	*Net-benefit RF* §	*Net-benefit LR* §	*Treat-all*	*Treat-none*			
Decision-curve summary								
	Standard FP penalty	12 – 50%	24 – 50%	baseline	0	–	–	–
	MRI triage (FP 0.20)	17 – 39%	17 – 50%	negative baseline	0	–	–	–
	Surgery triage (FP 1.00)	38 – 50%	38 – 50%	negative baseline	0	–	–	–
Stage / Task	*Landmark (m)*	*At-risk N*	*Events ≤ horizon*	*Event %*	*Age mean*	*Female %*	*BMI mean*	
Landmark demographics								
	**0**	1095	172	15.7	61.6	63.4	29.2	–
	**12**	1082	189	17.5	61.5	63.2	29.3	–
	**24**	1061	201	18.9	61.5	63.1	29.3	–
	**36**	1031	171	16.6	61.4	62.9	29.2	–
	**48**	994	376	37.8	61.3	63.3	29.2	–

† C-index is presented in the AUC column for Cox models (Harrell for the naive row; IPCW when indicated).

‡ Confidence intervals for Cox rows are calculated as mean ± 1.96 × (SD/√5), where SD is the fold standard deviation with ddof=0.

§ Positive net-benefit means the model curve remains above both baselines across the entire threshold range and by at least 0.002.

Table S21: Performance of knee-replacement risk models trained on biomarkers up to 48 months and tested on the 48-120 month horizon. Performance of knee-replacement risk models trained on automatically extracted biomarkers and demographics available up to 48 months and evaluated on the 48-120 month prediction horizon. Columns report discrimination (area under the ROC curve, or C-index for Cox models, with 95% confidence intervals), calibration slope, raw and isotonic-calibrated Brier scores, decision-curve net-benefit ranges, and demographic characteristics of knees still at risk at each landmark. Model-selection AUCs are limited to the 0-month landmark. Cox baselines use month-0 features only.

OA incidence short-term (complete-case, 48 → 96m)								
Stage / Task	*Landmark (m)*	*Model*	*AUC*	*95% CI low*	*95% CI high*			
Model-selection								
	**0**	Logistic Regression	0.723	0.694	0.754	–	–	–
	**0**	Random Forest	0.696	0.666	0.729	–	–	–
	**0**	XGBoost	0.680	0.650	0.711	–	–	–
	**0**	Soft-vote ensemble	0.711	0.680	0.742	–	–	–
	**0**	Stacking ensemble	0.721	0.692	0.750	–	–	–
Stage / Task	*Landmark (m)*	*Model*	*AUC*	*95% CI low*	*95% CI high*	*Calibration slope*	*Brier raw*	*Brier calib*
Discrimination & calibration								
Cox baselines†	**0**	Cox baseline (naïve)	0.714	0.683	0.746	–	–	–
	**0**	Cox baseline (IPCW)	0.718	0.686	0.749	–	–	–
Demographics-only baseline‡ *(age, sex, BMI), 48–96 month horizon*	**0**	Logistic Regression	0.533	0.495	0.573	0.991	0.252	0.207
	**0**	Random Forest	0.506	0.498	0.515	0.991	0.252	0.208
	**12**	Logistic Regression	0.575	0.521	0.626	0.995	0.247	0.159
	**12**	Random Forest	0.551	0.500	0.601	0.994	0.219	0.160
	**24**	Logistic Regression	0.515	0.476	0.557	0.988	0.251	0.204
	**24**	Random Forest	0.537	0.493	0.584	0.988	0.242	0.203
	**36**	Logistic Regression	0.533	0.494	0.572	0.990	0.252	0.175
	**36**	Random Forest	0.524	0.476	0.573	0.990	0.235	0.176
	**48**	Logistic Regression	0.520	0.462	0.578	0.983	0.253	0.203
	**48**	Random Forest	0.551	0.495	0.605	0.984	0.238	0.201
Biomarker models (cartilage and meniscus trajectories + age, sex, BMI), 48–96 month horizon	**0**	Logistic Regression	0.605	0.561	0.647	0.992	0.245	0.202
	**0**	Random Forest	0.564	0.520	0.605	0.992	0.231	0.206
	**12**	Logistic Regression	0.574	0.520	0.630	0.995	0.247	0.159
	**12**	Random Forest	0.581	0.531	0.628	0.995	0.197	0.158
	**24**	Logistic Regression	0.620	0.565	0.673	0.989	0.244	0.195
	**24**	Random Forest	0.554	0.502	0.601	0.989	0.231	0.201
	**36**	Logistic Regression	0.641	0.580	0.701	0.991	0.238	0.168
	**36**	Random Forest	0.595	0.535	0.655	0.990	0.203	0.171
	**48**	Logistic Regression	0.634	0.570	0.695	0.985	0.243	0.190
	**48**	Random Forest	0.600	0.541	0.669	0.985	0.223	0.197
Stage / Task	*Threshold %*	*Net-benefit RF* §	*Net-benefit LR* §	*Treat-all*	*Treat-none*			
Decision-curve summary								
	Standard FP penalty	22% - 42%	13% - 50%	baseline	0	–	–	–
	MRI triage (FP 0.20)	17% - 43%	17% - 50%	negative baseline	0	–	–	–
	Preventive counselling (FP 1.00)	–	35% - 50%	negative baseline	0	–	–	–
Stage / Task	*Landmark (m)*	*At-risk N*	*Events ≤ horizon*	*Event %*	*Age mean*	*Female %*	*BMI mean*	
Landmark demographics								
	**0**	743	220	29.6	60.6	62.6	29.0	–
	**12**	603	122	20.2	60.7	63.3	28.9	–
	**24**	491	142	28.9	60.5	62.9	28.8	–
	**36**	398	91	22.9	60.3	62.6	28.8	–
	**48**	341	97	28.4	60.3	60.1	28.7	–

† Cox rows use month-0 features only and the same 5-fold grouped CV. The AUC column lists C-index values (Harrell for the naive row; IPCW when indicated). 95% intervals are calculated as mean ± 1.96 × (SD/√5), where SD is the fold standard deviation with ddof=0.

‡ Inputs are age, sex, BMI only; same 5-fold grouped CV and 1,000-draw bootstrap; probabilities isotonic-calibrated, which yields the calibration slope and Brier columns on the same scale as the biomarker models.

§ Positive net-benefit means the model curve remains above both baselines across the entire threshold range and by at least 0.002.

Table S22: Osteoarthritis-incidence models, complete-case cohort, 48-month landmark $\rightarrow$ 96-month horizon. Performance of risk models trained on biomarkers and demographics recorded up to 48 months and evaluated for first radiographic OA (KL ≥ 2) occurring within the subsequent 48 months. Table entries include discrimination (AUC or C-index with 95% confidence intervals), calibration slope, raw and isotonic-calibrated Brier scores, decision-curve net-benefit ranges, and demographic characteristics of knees still at risk at each landmark. Model-selection AUCs are shown for the 0-month landmark only. Cox baselines rely on month-0 features†. Demographics-only baselines (age, sex, BMI) are listed at each landmark‡. Net-benefit windows are shown when the model curve exceeds both "treat-all" and "treat-none" by at least 0.002§.

OA incidence long-term (complete-case, 48 → 120m)								
Stage / Task	*Landmark (m)*	*Model*	*AUC*	*95% CI low*	*95% CI high*			
Model-selection								
	**0**	Logistic Regression	0.716	0.686	0.745	–	–	–
	**0**	Random Forest	0.702	0.666	0.731	–	–	–
	**0**	XGBoost	0.689	0.658	0.720	–	–	–
	**0**	Soft-vote ensemble	0.714	0.684	0.745	–	–	–
	**0**	Stacking ensemble	0.717	0.686	0.745	–	–	–
Stage / Task	*Landmark (m)*	*Model*	*AUC*	*95% CI low*	*95% CI high*	*Calibration slope*	*Brier raw*	*Brier calib*
Discrimination & calibration								
	**0**	Cox baseline (naïve)	0.704 †	0.684 ‡	0.724 ‡	–	–	–
	**0**	Cox baseline (IPCW)	0.713 †	0.696 ‡	0.730 ‡	–	–	–
	**0**	Logistic Regression	0.607	0.569	0.647	0.983	0.244	0.228
	**0**	Random Forest	0.600	0.560	0.641	0.982	0.241	0.229
	**12**	Logistic Regression	0.581	0.533	0.625	0.988	0.251	0.213
	**12**	Random Forest	0.558	0.512	0.601	0.988	0.237	0.214
	**24**	Logistic Regression	0.601	0.551	0.651	0.979	0.250	0.223
	**24**	Random Forest	0.577	0.526	0.624	0.976	0.245	0.228
	**36**	Logistic Regression	0.611	0.556	0.667	0.982	0.251	0.207
	**36**	Random Forest	0.584	0.534	0.637	0.982	0.234	0.211
	**48**	Logistic Regression	0.635	0.571	0.695	0.985	0.243	0.190
	**48**	Random Forest	0.600	0.535	0.664	0.985	0.223	0.197
Stage / Task	*Threshold %*	*Net-benefit RF §*	*Net-benefit LR §*	*Treat-all*	*Treat-none*			
Decision-curve summary								
	Standard FP penalty	22% - 42%	13% - 50%	baseline	0	–	–	–
	MRI triage (FP 0.20)	17% - 43%	17% - 50%	negative baseline	0	–	–	–
	Preventive counselling (FP 1.00)	–	35% - 50%	negative baseline	0	–	–	–
Stage / Task	*Landmark (m)*	*At-risk N*	*Events ≤ horizon*	*Event %*	*Age mean*	*Female %*	*BMI mean*	
Landmark demographics								
	**0**	743.0	291.0	39.2	60.6	62.6	29.0	–
	**12**	603.0	193.0	32.0	60.7	63.3	28.9	–
	**24**	491.0	181.0	36.9	60.5	62.9	28.8	–
	**36**	398.0	127.0	31.9	60.3	62.6	28.8	–
	**48**	341.0	97.0	28.4	60.3	60.1	28.7	–

† C-index is presented in the AUC column for Cox models (Harrell for the naive row; IPCW when indicated).

‡ Confidence intervals for Cox rows are calculated as mean ± 1.96 × (SD/√5), where SD is the fold standard deviation with ddof=0.

§ Positive net-benefit means the model curve remains above both baselines across the entire threshold range and by at least 0.002.

Table S23: Osteoarthritis-incidence models, complete-case cohort, 48-month landmark → 120-month horizon. Same analytic pipeline as Table S22, but the prediction window extends to 120 months (48 + 72m). Administrative censoring is applied at 144 months to accommodate late outcomes. All metrics, decision-curve windows, and demographic counts follow the definitions given above.

OA incidence short-term (mean-imputed, 48 → 96m)								
Stage / Task	*Landmark (m)*	*Model*	*AUC*	*95% CI low*	*95% CI high*			
Model-selection								
	**0**	Logistic Regression	0.723	0.693	0.753	–	–	–
	**0**	Random Forest	0.696	0.665	0.727	–	–	–
	**0**	XGBoost	0.680	0.649	0.711	–	–	–
	**0**	Soft-vote ensemble	0.711	0.679	0.738	–	–	–
	**0**	Stacking ensemble	0.721	0.693	0.75	–	–	–
Stage / Task	*Landmark (m)*	*Model*	*AUC*	*95% CI low*	*95% CI high*	*Calibration slope*	*Brier raw*	*Brier calib*
Discrimination & calibration								
	**0**	Cox baseline (naïve)	0.714 †	0.683 ‡	0.746 ‡	–	–	–
	**0**	Cox baseline (IPCW)	0.718 †	0.686 ‡	0.749 ‡	–	–	–
	**0**	Logistic Regression	0.605	0.567	0.647	0.992	0.245	0.202
	**0**	Random Forest	0.564	0.522	0.607	0.992	0.231	0.206
	**12**	Logistic Regression	0.559	0.503	0.614	0.995	0.248	0.154
	**12**	Random Forest	0.578	0.528	0.628	0.995	0.193	0.155
	**24**	Logistic Regression	0.595	0.549	0.639	0.993	0.247	0.186
	**24**	Random Forest	0.592	0.547	0.638	0.992	0.212	0.183
	**36**	Logistic Regression	0.627	0.569	0.683	0.994	0.238	0.146
	**36**	Random Forest	0.658	0.604	0.711	0.994	0.178	0.144
	**48**	Logistic Regression	0.620	0.565	0.676	0.993	0.247	0.166
	**48**	Random Forest	0.681	0.632	0.733	0.992	0.187	0.157
Stage / Task	*Threshold %*	*Net-benefit RF §*	*Net-benefit LR §*	*Treat-all*	*Treat-none*			
Decision-curve summary								
	Standard FP penalty	3% - 50%	13% - 41%	baseline	0	–	–	–
	MRI triage (FP 0.20)	15% - 50%	17% - 42%	negative baseline	0	–	–	–
	Preventive counselling (FP 1.00)	38% - 50%	–	negative baseline	0	–	–	–
Stage / Task	*Landmark (m)*	*At-risk N*	*Events ≤ horizon*	*Event %*	*Age mean*	*Female %*	*BMI mean*	
Landmark demographics								
	**0**	743	220	29.6	60.6	62.6	29.0	–
	**12**	652	128	19.6	60.7	63.8	29.0	–
	**24**	611	159	26.0	60.6	63.5	28.9	–
	**36**	557	105	18.9	60.6	62.7	28.9	–
	**48**	524	116	22.1	60.6	62.0	28.9	–

† C-index is presented in the AUC column for Cox models (Harrell for the naive row; IPCW when indicated).

‡ Confidence intervals for Cox rows are calculated as mean ± 1.96 × (SD/√5), where SD is the fold standard deviation with ddof=0.

§ Positive net-benefit means the model curve remains above both baselines across the entire threshold range and by at least 0.002.

Table S24: Osteoarthritis-incidence models, mean-imputed cohort, 48-month landmark → 96-month horizon. Sensitivity analysis retaining knees with partial biomarker data through per-visit mean imputation. Models are trained on the imputed feature set available up to 48 months and assessed for OA conversion by 96 months. Discrimination, calibration, decision-curve net-benefit ranges, and cohort demographics are reported as in Table S22, enabling side-by-side comparison with the complete-case results.

OA incidence long-term (mean-imputed, 48 → 120m)								
Stage / Task	*Landmark (m)*	*Model*	*AUC*	*95% CI low*	*95% CI high*			
Model-selection								
	**0**	Logistic Regression	0.716	0.685	0.745	–	–	–
	**0**	Random Forest	0.702	0.672	0.732	–	–	–
	**0**	XGBoost	0.689	0.660	0.720	–	–	–
	**0**	Soft-vote ensemble	0.714	0.682	0.744	–	–	–
	**0**	Stacking ensemble	0.717	0.689	0.747	–	–	–
Stage / Task	*Landmark (m)*	*Model*	*AUC*	*95% CI low*	*95% CI high*	*Calibration slope*	*Brier raw*	*Brier calib*
Discrimination & calibration								
	**0**	Cox baseline (naïve)	0.704 †	0.684 ‡	0.724 ‡	–	–	–
	**0**	Cox baseline (IPCW)	0.713 †	0.696 ‡	0.730 ‡	–	–	–
	**0**	Logistic Regression	0.609	0.569	0.651	0.983	0.244	0.228
	**0**	Random Forest	0.600	0.558	0.643	0.982	0.241	0.229
	**12**	Logistic Regression	0.585	0.541	0.631	0.990	0.248	0.206
	**12**	Random Forest	0.577	0.532	0.623	0.989	0.230	0.208
	**24**	Logistic Regression	0.581	0.535	0.624	0.987	0.255	0.216
	**24**	Random Forest	0.609	0.565	0.654	0.988	0.230	0.213
	**36**	Logistic Regression	0.608	0.558	0.654	0.991	0.247	0.189
	**36**	Random Forest	0.625	0.574	0.674	0.992	0.214	0.188
	**48**	Logistic Regression	0.619	0.562	0.678	0.993	0.247	0.166
	**48**	Random Forest	0.681	0.628	0.729	0.992	0.187	0.157
Stage / Task	*Threshold %*	*Net-benefit RF §*	*Net-benefit LR §*	*Treat-all*	*Treat-none*			
Decision-curve summary								
	Standard FP penalty	3% - 50%	13% - 41%	baseline	0	–	–	–
	MRI triage (FP 0.20)	15% - 50%	17% - 42%	negative baseline	0	–	–	–
	Preventive counselling (FP 1.00)	38% - 50%	–	negative baseline	0	–	–	–
Stage / Task	*Landmark (m)*	*At-risk N*	*Events ≤ horizon*	*Event %*	*Age mean*	*Female %*	*BMI mean*	
Landmark demographics								
	**0**	743	291	39.2	60.6	62.6	29.0	–
	**12**	652	200	30.7	60.7	63.8	29.0	–
	**24**	611	203	33.2	60.6	63.5	28.9	–
	**36**	557	149	26.8	60.6	62.7	28.9	–
	**48**	524	116	22.1	60.6	62.0	28.9	–
† C-index is presented in the AUC column for Cox models (Harrell for the naive row; IPCW when indicated).								
‡ Confidence intervals for Cox rows are calculated as mean ± 1.96 × (SD/√5), where SD is the fold standard deviation with ddof=0.								
§ Positive net-benefit means the model curve remains above both baselines across the entire threshold range and by at least 0.002.								

Table S25: Osteoarthritis-incidence models, mean-imputed cohort, 48-month landmark → 120-month horizon. Long-horizon sensitivity run corresponding to Table S23 but using the mean-imputed feature matrix. Results verify that model performance and net-benefit patterns persist when incomplete biomarker series are retained. Column definitions and footnotes are identical to those in Tables S23–S24.

10. Data Index for external repository

All study-generated data tables (codes **D1–D57**) are available in an open Figshare repository at: https://doi.org/10.6084/m9.figshare.29633207. These tables are listed here for reference only and are excluded from the Supplementary Materials PDF to keep the file size manageable.

Detailed DICE-score results from baseline and finetuning

Code	Table Title
D1	Subject-Level Segmentation Results and Experimental Details for Finetuning Experiments on Knee_2D_MAPSS-echo1_Research_Compositional_39
D2	Dataset-Level Mean Segmentation Results and Experimental Details for Finetuning Experiments on Knee_2D_MAPSS-echo1_Research_Compositional_39
D3	Subject-Level Segmentation Results and Experimental Details for Finetuning Experiments on Spine_2D_T1ax_Clinical_Anatomical_59
D4	Dataset-Level Mean Segmentation Results and Experimental Details for Finetuning Experiments on Spine_2D_T1ax_Clinical_Anatomical_59
D5	Subject-Level Segmentation Results and Experimental Details for Finetuning Experiments on Spine_2D_T1sag_Clinical_Anatomical_111
D6	Dataset-Level Mean Segmentation Results and Experimental Details for Finetuning Experiments on Spine_2D_T1sag_Clinical_Anatomical_111
D7	Subject-Level Segmentation Results and Experimental Details for Finetuning Experiments on Spine_2D_T2ax_Clinical_Anatomical_38
D8	Dataset-Level Mean Segmentation Results and Experimental Details for Finetuning Experiments on Spine_2D_T2ax_Clinical_Anatomical_38
D9	Subject-Level Segmentation Results and Experimental Details for Finetuning Experiments on Spine_2D_T1sag_Clinical_Anatomical_88
D10	Dataset-Level Mean Segmentation Results and Experimental Details for Finetuning Experiments on Spine_2D_T1sag_Clinical_Anatomical_88
D11	Subject-Level Segmentation Results and Experimental Details for Finetuning Experiments on Shoulder_3D_CUBE_Research_Anatomical_28
D12	Dataset-Level Mean Segmentation Results and Experimental Details for Finetuning Experiments on Shoulder_3D_CUBE_Research_Anatomical_28
D13	Subject-Level Segmentation Results and Experimental Details for Finetuning Experiments on Knee_3D_undersampled_CUBE_Research_Anatomical_50
D14	Dataset-Level Mean Segmentation Results and Experimental Details for Finetuning Experiments on Knee_3D_undersampled_CUBE_Research_Anatomical_50
D15	Subject-Level Segmentation Results and Experimental Details for Finetuning Experiments on Hip_3D_CUBE_Research_Anatomical_42
D16	Dataset-Level Mean Segmentation Results and Experimental Details for Finetuning Experiments on Hip_3D_CUBE_Research_Anatomical_42
D17	Subject-Level Segmentation Results and Experimental Details for Finetuning Experiments on Knee_3D_DESS_Research_Anatomical_86
D18	Dataset-Level Mean Segmentation Results and Experimental Details for Finetuning Experiments on Knee_3D_DESS_Research_Anatomical_86
D19	Subject-Level Segmentation Results and Experimental Details for Finetuning Experiments on Thigh_2D_T1ax_Clinical_Anatomical_50
D20	Dataset-Level Mean Segmentation Results and Experimental Details for Finetuning Experiments on Thigh_2D_T1ax_Clinical_Anatomical_50
D21	Subject-Level Segmentation Results and Experimental Details for Finetuning Experiments on Knee_2D_MAPSS-echo1_Research_Compositional_22
D22	Dataset-Level Mean Segmentation Results and Experimental Details for Finetuning Experiments on Knee_2D_MAPSS-echo1_Research_Compositional_22
D23	Subject-Level Segmentation Results and Experimental Details for Finetuning Experiments on Knee_3D_CUBE_Research_Anatomical_300
D24	Dataset-Level Mean Segmentation Results and Experimental Details for Finetuning Experiments on Knee_3D_CUBE_Research_Anatomical_300

Mixed-model data preparation and checks

Code	Table Title
D25	Original Dataset Prior to Preprocessing for Hierarchical Mixed-Effects Modeling
D26	Final Preprocessed Dataset After Imputation, Encoding, Scaling, and Feature Selection for Hierarchical Mixed-Effects Modeling

Biomarker metric results by dataset and label

Code	Table Title
D27	Subject-Level Bilateral Muscle Volume (cm³) Comparison: Manual Annotation vs. Automated Model Prediction for Thigh_2D_T1ax_Clinical_Anatomical_50 Dataset
D28	Slice-Level Bilateral Muscle Volume Metrics: Manual Annotation vs. Automated Model Prediction for Thigh_2D_T1ax_Clinical_Anatomical_50 Dataset
D29	Subject-Level Mean Cartilage Thickness (mm) Comparison: Manual Annotation vs. Automated Model Prediction for Knee_3D_DESS_Research_Anatomical_86 Dataset
D30	Slice-Level Mean Cartilage Thickness (mm) Comparison: Manual Annotation vs. Automated Model Prediction for Knee_3D_DESS_Research_Anatomical_86 Dataset
D31	Subject-Level Mean Cartilage Thickness (mm) Comparison: Manual Annotation vs. Automated Model Prediction for Knee_3D_CUBE_Research_Anatomical_300
D32	Slice-Level Mean Cartilage Thickness (mm) Comparison: Manual Annotation vs. Automated Model Prediction for Knee_3D_CUBE_Research_Anatomical_300
D33	Subject-Level Bone Volume (cm³) Comparison: Manual Annotation vs. Automated Model Prediction for Knee_3D_CUBE_Research_Anatomical_300
D34	Slice-Level Bone Volume (cm³) Comparison: Manual Annotation vs. Automated Model Prediction for Knee_3D_CUBE_Research_Anatomical_300
D35	Subject-Level Mean T1 Rho Value (ms) Comparison: Manual Annotation vs. Automated Model Prediction for Knee_2D_MAPSS-echo1_Research_Compositional_22 Dataset
D36	Slice-Level T1 Rho Value (ms) Comparison: Manual Annotation vs. Automated Model Prediction for Knee_2D_MAPSS-echo1_Research_Compositional_22 Dataset
D37	Subject-Level Mean T2 Value (ms) Comparison: Manual Annotation vs. Automated Model Prediction for Knee_2D_MAPSS-echo1_Research_Compositional_22 Dataset
D38	Slice-Level T2 Value (ms) Comparison: Manual Annotation vs. Automated Model Prediction for Knee_2D_MAPSS-echo1_Research_Compositional_22 Dataset
D39	Subject-Level Mean T1 Rho Value (ms) Comparison: Manual Annotation vs. Automated Model Prediction for Knee_2D_MAPSS-echo1_Research_Compositional_39
D40	Slice-Level T1 Rho Value (ms) Comparison: Manual Annotation vs. Automated Model Prediction for Knee_2D_MAPSS-echo1_Research_Compositional_39
D41	Subject-Level Mean T2 Value (ms) Comparison: Manual Annotation vs. Automated Model Prediction for Knee_2D_MAPSS-echo1_Research_Compositional_39
D42	Slice-Level T2 Value (ms) Comparison: Manual Annotation vs. Automated Model Prediction for Knee_2D_MAPSS-echo1_Research_Compositional_39
D43	Subject-Level Bone Volume (cm³) Comparison: Manual Annotation vs. Automated Model Prediction for Hip_3D_CUBE_Research_Anatomical_42 Dataset
D44	Slice-Level Bone Volume (cm³) Comparison: Manual Annotation vs. Automated Model Prediction for Hip_3D_CUBE_Research_Anatomical_42 Dataset
D45	Subject-Level Bilateral Muscle Volume (cm³) Comparison: Manual Annotation vs. Automated Model Prediction for Spine_2D_T1ax_Clinical_Anatomical_59
D46	Slice-Level Bilateral Muscle Volume (cm³) Comparison: Manual Annotation vs. Automated Model Prediction for Spine_2D_T1ax_Clinical_Anatomical_59
D47	Subject-Level Bone Volume (cm³) Comparison: Manual Annotation vs. Automated Model Prediction for Knee_3D_undersampled_CUBE_Research_Anatomical_50
D48	Slice-Level Bone Volume (cm³) Comparison: Manual Annotation vs. Automated Model Prediction for Knee_3D_undersampled_CUBE_Research_Anatomical_50
D49	Subject-Level Mean Cartilage Thickness (mm) Comparison: Manual Annotation vs. Automated Model Prediction for Knee_3D_undersampled_CUBE_Research_Anatomical_[illegible]
D50	Slice-Level Mean Cartilage Thickness (mm) Comparison: Manual Annotation vs. Automated Model Prediction for Knee_3D_undersampled_CUBE_Research_Anatomical_50
D51	Subject-Level Max Disc Height (mm) Comparison: Manual Annotation vs. Automated Model Prediction for Spine_2D_T1sag_Clinical_Anatomical_88 Dataset
D52	Slice-Level Disc Height (mm) Comparison: Manual Annotation vs. Automated Model Prediction for Spine_2D_T1sag_Clinical_Anatomical_88 Dataset

Autolabel pipeline evaluation

Code	Table Title
D53	Subject-Level DICE Scores for Evaluation of the Autolabeling System Across Five Musculoskeletal MRI Datasets
D54	Dataset-Level Mean DICE Scores for Evaluation of the Autolabeling System Across Five Musculoskeletal MRI Datasets

Failure-case (tail) segmentation performance

Code	Table Title
D55	Subject-level Dice tail summaries across all segmentation experiments
D56	Table 1 model configurations: tissue-level Dice tail summaries
D57	Table 1 model configurations: subject-mean Dice tail summaries (compact)

11. References

[1] Annarumma, M. *et al.* Automated Triaging of Adult Chest Radiographs with Deep Artificial Neural Networks. *Radiology* **291**, 196–202 (2019).

[2] O'Neill, T. J. *et al.* Active Reprioritization of the Reading Worklist Using Artificial Intelligence Has a Beneficial Effect on the Turnaround Time for Interpretation of Head CT with Intracranial Hemorrhage. *Radiology: Artificial Intelligence* **3**, e200024 (2021).

[3] Batra, K., Xi, Y., Bhagwat, S., Espino, A. & Peshock, R. M. Radiologist Worklist Reprioritization Using Artificial Intelligence: Impact on Report Turnaround Times for CTPA Examinations Positive for Acute Pulmonary Embolism. *American Journal of Roentgenology* **221**, 324–333 (2023).

[4] Gillies, R. J., Kinahan, P. E. & Hricak, H. Radiomics: Images Are More than Pictures, They Are Data. *Radiology* **278**, 563–577 (2016).

[5] Eckstein, F. & Wirth, W. Quantitative Cartilage Imaging in Knee Osteoarthritis. *Arthritis* **2011**, 1–19 (2011).

[6] Cieza, A. *et al.* Global estimates of the need for rehabilitation based on the Global Burden of Disease study 2019: a systematic analysis for the Global Burden of Disease Study 2019. *The Lancet* **396**, 2006–2017 (2020).

[7] Hartvigsen, J. *et al.* What low back pain is and why we need to pay attention. *The Lancet* **391**, 2356–2367 (2018).

[8] Williams, A. *et al.* Musculoskeletal conditions may increase the risk of chronic disease: a systematic review and meta-analysis of cohort studies. *BMC Medicine* **16**, 167 (2018).

[9] Tajbakhsh, N. *et al.* Embracing imperfect datasets: A review of deep learning solutions for medical image segmentation. *Medical Image Analysis* **63**, 101693 (2020).

[10] Pons, C. *et al.* Quantifying skeletal muscle volume and shape in humans using MRI: A systematic review of validity and reliability. *PLOS ONE* **13**, e0207847 (2018).

[11] Tunset, A., Kjaer, P., Samir Chreiteh, S. & Secher Jensen, T. A method for quantitative measurement of lumbar intervertebral disc structures: an intra- and inter-rater agreement and reliability study. *Chiropractic & Manual Therapies* **21**, 26 (2013).

[12] Kirillov, A. *et al.* Segment anything. *Proceedings of the IEEE/CVF International Conference on Computer Vision (ICCV)* 4015–4026 (2023).

[13] Ravi, N. *et al.* Sam 2: Segment anything in images and videos. *International Conference on Learning Representations (ICLR)* (2025).

[14] Ma, J. *et al.* Segment anything in medical images. *Nature Communications* **15**, 654 (2024).

[15] Committee, R. T. Integrating the Healthcare Enterprise. Radiology Technical Framework Supplement: AI Results (AIR). Tech. Rep. Rev. 1.3, IHE International, Inc. (2025).

[16] Leiner, T., Bennink, E., Mol, C. P., Kuijf, H. J. & Veldhuis, W. B. Bringing AI to the clinic: blueprint for a vendor-neutral AI deployment infrastructure. *Insights into Imaging* **12**, 11 (2021).

[17] Brink, L. *et al.* ACR's Connect and AI-LAB technical framework. *JAMIA open* **5**, ooac094 (2022).

[18] Eckstein, F. *et al.* Quantitative MRI measures of cartilage predict knee replacement: a case–control study from the Osteoarthritis Initiative. *Annals of the Rheumatic Diseases* **72**, 707–714 (2013).

[19] Kwoh, C. *et al.* Predicting knee replacement in participants eligible for disease-modifying osteoarthritis drug treatment with structural endpoints. *Osteoarthritis and Cartilage* **28**, 782–791 (2020).

[20] Van Houwelingen, H. & Putter, H. *Dynamic Prediction in Clinical Survival Analysis* 1 edn (CRC Press, 2011).

[21] Van Houwelingen, H. C. & Putter, H. Dynamic predicting by landmarking as an alternative for multi-state modeling: an application to acute lymphoid leukemia data. *Lifetime Data Analysis* **14**, 447–463 (2008).

[22] Vickers, A. J. & Elkin, E. B. Decision Curve Analysis: A Novel Method for Evaluating Prediction Models. *Medical Decision Making* **26**, 565–574 (2006).

[23] Zhao, T. *et al.* A foundation model for joint segmentation, detection and recognition of biomedical objects across nine modalities. *Nature Methods* **22**, 166–176 (2025).

[24] Tolpadi, A. A. *et al.* K2S Challenge: From Undersampled K-Space to Automatic Segmentation. *Bioengineering* **10**, 267 (2023).

[25] Pedoia, V. *et al.* Principal component analysis-T1ρ voxel based relaxometry of the articular cartilage: a comparison of biochemical patterns in osteoarthritis and anterior cruciate ligament subjects. *Quantitative Imaging in Medicine and Surgery* **6**, 623–633 (2016).

[26] Peterfy, C. G., Schneider, E. & Nevitt, M. The osteoarthritis initiative: report on the design rationale for the magnetic resonance imaging protocol for the knee. *Osteoarthritis and Cartilage* **16**, 1433–1441 (2008).

[27] White Paper: Imorphics OA Knee MRI Measurements. Tech. Rep., Stryker Imorphics (2017). URL www.imorphics.com.

[28] Hess, M. *et al.* Deep Learning for Multi-Tissue Segmentation and Fully Automatic Personalized Biomechanical Models from BACPAC Clinical Lumbar Spine MRI. *Pain Medicine* **24**, S139–S148 (2023).

[29] Thahakoya, R. Evaluating the relationship of proximal bone shape asymmetry with cartilage health and biomechanics in patients with hip OA. *International Society for Magnetic Resonance in Medicine (ISMRM)* (2023).

[30] Lee, S. *et al.* Magnetic resonance rotator cuff fat fraction and its relationship with tendon tear severity and subject characteristics. *Journal of Shoulder and Elbow Surgery* **24**, 1442–1451 (2015).

[31] Nardo, L. *et al.* Quantitative assessment of fat infiltration in the rotator cuff muscles using water-fat MRI: Fat Infiltration in the Rotator Cuff Muscles. *Journal of Magnetic Resonance Imaging* **39**, 1178–1185 (2014).

[32] Dice, L. R. Measures of the Amount of Ecologic Association Between Species. *Ecology* **26**, 297–302 (1945).

[33] Jaccard, P. Étude comparative de la distribution florale dans une portion des alpes et des jura. *Bull Soc Vaudoise Sci Nat* **37**, 547–579 (1901).

[34] Friedman, M. The Use of Ranks to Avoid the Assumption of Normality Implicit in the Analysis of Variance. *Journal of the American Statistical Association* **32**, 675–701 (1937).

[35] Wilcoxon, F. Individual Comparisons by Ranking Methods. *Biometrics Bulletin* **1**, 80 (1945).

[36] Benjamini, Y. & Hochberg, Y. Controlling the False Discovery Rate: A Practical and Powerful Approach to Multiple Testing. *Journal of the Royal Statistical Society Series B: Statistical Methodology* **57**, 289–300 (1995).

[37] Loshchilov, I. & Hutter, F. Decoupled weight decay regularization. *International Conference on Learning Representations (ICLR)* (2019).

[38] Loshchilov, I. & Hutter, F. Sgdr: Stochastic gradient descent with warm restarts. *International Conference on Learning Representations (ICLR)* (2017).

[39] Nickolls, J., Buck, I., Garland, M. & Skadron, K. Scalable Parallel Programming with CUDA: Is CUDA the parallel programming model that application developers have been waiting for? *Queue* **6**, 40–53 (2008).

[40] Stekhoven, D. J. & Bühlmann, P. MissForest—non-parametric missing value imputation for mixed-type data. *Bioinformatics* **28**, 112–118 (2012).

[41] Kutner, M. H., Nachtsheim, C. J., Neter, J. & Li, W. (eds) *Applied linear statistical models* 5. ed., internat. ed edn. McGraw-Hill/Irwin series Operations and decision sciences (McGraw-Hill, Boston, Mass., 2005).

[42] Shapiro, S. S. & Wilk, M. B. An analysis of variance test for normality (complete samples). *Biometrika* **52**, 591–611 (1965).

[43] Brown, M. B. & Forsythe, A. B. Robust tests for the equality of variances. *Journal of the American Statistical Association* **69**, 364–367 (1974).

[44] Koo, T. K. & Li, M. Y. A Guideline of Selecting and Reporting Intraclass Correlation Coefficients for Reliability Research. *Journal of Chiropractic Medicine* **15**, 155–163 (2016).

[45] Bland, J. M. & Altman, D. G. Statistical methods for assessing agreement between two methods of clinical measurement. *Lancet (London, England)* **1**, 307–310 (1986).

[46] Rasmussen, C. E. & Williams, C. K. I. *Gaussian Processes for Machine Learning* (The MIT Press, 2005).

[47] Jocher, G., Qiu, J. & Chaurasia, A. Ultralytics YOLO (2023). URL https://github.com/ultralytics/ultralytics.

[48] Zou, H. & Hastie, T. Regularization and Variable Selection Via the Elastic Net. *Journal of the Royal Statistical Society Series B: Statistical Methodology* **67**, 301–320 (2005).

[49] Chen, T. & Guestrin, C. Xgboost: A scalable tree boosting system. *Proc. 22nd ACM SIGKDD Int. Conf. on Knowledge Discovery and Data Mining (KDD), San Francisco, CA, USA* 785–794 (2016).

[50] Pedregosa, F. *et al.* Scikit-learn: Machine Learning in Python. *Journal of Machine Learning Research* **12**, 2825–2830 (2011).

[51] Hoyer, G. *et al.* Foundations of a knee joint digital twin from qMRI biomarkers for osteoarthritis and knee replacement. *npj Digital Medicine* **8** (2025).

[52] Breiman, L. Random Forests. *Machine Learning* **45**, 5–32 (2001).

[53] Zadrozny, B. & Elkan, C. Transforming classifier scores into accurate multiclass probability estimates. *Proc. 8th ACM SIGKDD Int. Conf. on Knowledge Discovery and Data Mining (KDD)* 694–699 (2002).

[54] Cox, D. R. Regression Models and Life-Tables. *Journal of the Royal Statistical Society Series B: Statistical Methodology* **34**, 187–202 (1972).

[55] Harrell, F. E. *Regression Modeling Strategies: With Applications to Linear Models, Logistic and Ordinal Regression, and Survival Analysis* Springer Series in Statistics (Springer International Publishing, Cham, 2015).

[56] Uno, H., Cai, T., Pencina, M. J., D'Agostino, R. B. & Wei, L. J. On the C-statistics for evaluating overall adequacy of risk prediction procedures with censored survival data. *Statistics in Medicine* **30**, 1105–1117 (2011).

[57] Brier, G. W. Verification of forecasts expressed in terms of probability. *Monthly Weather Review* **78**, 1–3 (1950).

[58] Lundberg, S. M. *et al.* From local explanations to global understanding with explainable AI for trees. *Nature Machine Intelligence* **2**, 56–67 (2020).

[59] Lundberg, S. M. & Lee, S.-I. A unified approach to interpreting model predictions. *Advances in Neural Information Processing Systems (NeurIPS)* **30**, 4768–4777 (2017).

[60] Haig, A. J. Paraspinal denervation and the spinal degenerative cascade. *The Spine Journal* **2**, 372–380 (2002).

[61] Gallo, M. *et al.* T1ρ and T2 relaxation times are associated with progression of hip osteoarthritis. *Osteoarthritis and Cartilage* **24**, 1399–1407 (2016).

[62] Baum, T. *et al.* Association of Quadriceps Muscle Fat With Isometric Strength Measurements in Healthy Males Using Chemical Shift Encoding-Based Water-Fat Magnetic Resonance Imaging:. *Journal of Computer Assisted Tomography* **40**, 447–451 (2016).

[63] Bhattacharjee, R. *et al.* Exploring Bilateral Thigh Normalized-Lean-Muscle And Fat Volume Associations With Knee Cartilage Thickness And Functional Parameters In Radiographic Hip Oa Patients. *Osteoarthritis and Cartilage* **31**, S110–S111 (2023).

[64] Davison, M. J. *et al.* Lean muscle volume of the thigh has a stronger relationship with muscle power than muscle strength in women with knee osteoarthritis. *Clinical Biomechanics* **41**, 92–97 (2017).

[65] Iriondo, C. *et al.* Towards understanding mechanistic subgroups of osteoarthritis: 8-year cartilage thickness trajectory analysis. *Journal of Orthopaedic Research* **39**, 1305–1317 (2021).

[66] Cummings, J. *et al.* The knee connectome: A novel tool for studying spatiotemporal change in cartilage thickness. *Journal of Orthopaedic Research* **42**, 43–53 (2024).

[67] Morales, A. G. *et al.* Uncovering associations between data-driven learned qMRI biomarkers and chronic pain. *Scientific Reports* **11**, 21989 (2021).

[68] Pedoia, V., Lee, J., Norman, B., Link, T. & Majumdar, S. Diagnosing osteoarthritis from T2 maps using deep learning: an analysis of the entire Osteoarthritis Initiative baseline cohort. *Osteoarthritis and Cartilage* **27**, 1002–1010 (2019).

[69] Han, M., Tibrewala, R., Bahroos, E., Pedoia, V. & Majumdar, S. Magnetization-prepared spoiled gradient-echo snapshot imaging for efficient measurement of R $_2$ -R $_{1\rho}$ in knee cartilage. *Magnetic Resonance in Medicine* **87**, 733–745 (2022).

[70] Carballido-Gamio, J., Joseph, G. B., Lynch, J. A., Link, T. M. & Majumdar, S. Longitudinal analysis of MRI T_2 knee cartilage laminar organization in a subset of patients from the osteoarthritis initiative: A texture approach. *Magnetic Resonance in Medicine* **65**, 1184–1194 (2011).

[71] Iriondo, C., Pedoia, V. & Majumdar, S. Lumbar intervertebral disc characterization through quantitative MRI analysis: An automatic voxel-based relaxometry approach. *Magnetic Resonance in Medicine* **84**, 1376–1390 (2020).

[72] Roach, K. E., Bird, A. L., Pedoia, V., Majumdar, S. & Souza, R. B. Automated evaluation of hip abductor muscle quality and size in hip osteoarthritis: Localized muscle regions are strongly associated with overall muscle quality. *Magnetic Resonance Imaging* **111**, 237–245 (2024).

[73] Hodges, P. W. & Danneels, L. Changes in Structure and Function of the Back Muscles in Low Back Pain: Different Time Points, Observations, and Mechanisms. *Journal of Orthopaedic & Sports Physical Therapy* **49**, 464–476 (2019).

[74] Goubert, D., Oosterwijck, J. V., Meeus, M. & Danneels, L. Structural Changes of Lumbar Muscles in Non-specific Low Back Pain: A Systematic Review. *Pain Physician* **19**, E985–E1000 (2016).

[75] Virtanen, P. *et al.* SciPy 1.0: fundamental algorithms for scientific computing in Python. *Nature Methods* **17**, 261–272 (2020).

[76] Vallat, R. Pingouin: statistics in Python. *Journal of Open Source Software* **3**, 1026 (2018).

[77] Biewald, L. Experiment Tracking with Weights and Biases (2020). URL https://www.wandb.com/.

[78] Paszke, A. *et al.* PyTorch: An Imperative Style, High-Performance Deep Learning Library (2019).

[79] Diaz-Pinto, A. *et al.* MONAI Label: A framework for AI-assisted interactive labeling of 3D medical images. *Medical Image Analysis* **95** (2024).
