# Supplementary material for: Clinical utility of foundation models in musculoskeletal MRI for biomarker fidelity and predictive outcomes
Source: NPJ Digit Med. 2026 Mar 24;9:383. doi: 10.1038/s41746-026-02520-w (PMC13187405; doi:10.1038/s41746-026-02520-w)
Supplement: Supplementary file 2 — Reporting Summary [file 41746_2026_2520_MOESM2_ESM.pdf]

Corresponding author(s): Gabrielle Hoyer

Last updated by author(s): Feb 3, 2026

Reporting Summary

Nature Portfolio wishes to improve the reproducibility of the work that we publish. This form provides structure for consistency and transparency in reporting. For further information on Nature Portfolio policies, see our Editorial Policies and the Editorial Policy Checklist.

Statistics

For all statistical analyses, confirm that the following items are present in the figure legend, table legend, main text, or Methods section.

n/a	Confirmed	
☐	☒	The exact sample size (n) for each experimental group/condition, given as a discrete number and unit of measurement
☐	☒	A statement on whether measurements were taken from distinct samples or whether the same sample was measured repeatedly
☐	☒	The statistical test(s) used AND whether they are one- or two-sided<br>*Only common tests should be described solely by name; describe more complex techniques in the Methods section.*
☐	☒	A description of all covariates tested
☐	☒	A description of any assumptions or corrections, such as tests of normality and adjustment for multiple comparisons
☐	☒	A full description of the statistical parameters including central tendency (e.g. means) or other basic estimates (e.g. regression coefficient) AND variation (e.g. standard deviation) or associated estimates of uncertainty (e.g. confidence intervals)
☐	☒	For null hypothesis testing, the test statistic (e.g. F, t, r) with confidence intervals, effect sizes, degrees of freedom and P value noted<br>*Give P values as exact values whenever suitable.*
☒	☐	For Bayesian analysis, information on the choice of priors and Markov chain Monte Carlo settings
☐	☒	For hierarchical and complex designs, identification of the appropriate level for tests and full reporting of outcomes
☐	☒	Estimates of effect sizes (e.g. Cohen's d, Pearson's r), indicating how they were calculated

Our web collection on statistics for biologists contains articles on many of the points above.

Software and code

Policy information about availability of computer code

Data collection	This study is a secondary analysis of existing MRI datasets. Osteoarthritis Initiative (OAI) MRI scans and associated cohort variables were accessed via the OAI data portal under registration and data use agreement. Additional institution-specific MRI datasets were accessed under institutional approvals and data governance as described in the manuscript. No new participant recruitment or prospective data collection was performed.
Data analysis	Preprocessing, model fine-tuning, inference, and evaluation were performed using custom Python code, in addition to standard open-source libraries. Code, configuration files, and reproducibility documentation are available at https://github.com/gabbieHoyer/AutoMedLabel. Additional implementation details are provided in the Supplementary Engineering Framework.

For manuscripts utilizing custom algorithms or software that are central to the research but not yet described in published literature, software must be made available to editors and reviewers. We strongly encourage code deposition in a community repository (e.g. GitHub). See the Nature Portfolio guidelines for submitting code & software for further information.

Data

Policy information about availability of data

All manuscripts must include a data availability statement. This statement should provide the following information, where applicable:

- Accession codes, unique identifiers, or web links for publicly available datasets
- A description of any restrictions on data availability
- For clinical datasets or third party data, please ensure that the statement adheres to our policy

All Supplementary Tables (S0–S25) and study-generated Data Tables (D1–D57) cited in the manuscript are publicly available at Figshare: https://doi.org/10.6084/m9.figshare.29633207. Osteoarthritis Initiative (OAI) MRI scans can be accessed via the OAI data portal with registration and data use agreement. Additional institution-specific MRI datasets are subject to institutional review board restrictions; deidentified versions are available from the corresponding author upon reasonable request.

Fine-tuned segmentation weights used in this study will be deposited in a public repository at the time of publication. Versioned metadata describing training datasets, label maps, and inference settings will be included to support reproduction and external validation.

Research involving human participants, their data, or biological material

Policy information about studies with human participants or human data. See also policy information about sex, gender (identity/presentation), and sexual orientation and race, ethnicity and racism.

Reporting on sex and gender	Sex and age were available for most participants and were analyzed as covariates in the hierarchical mixed-effects models. We did not collect new demographic information, and we did not perform a dedicated sex-based subgroup analysis beyond the validation and modeling procedures described in the manuscript. Demographic summaries are reported in the main text and Supplementary Tables where applicable.
Reporting on race, ethnicity, or other socially relevant groupings	Race and ethnicity were not consistently available across the datasets used in this study. For that reason, we did not conduct race- or ethnicity-based analyses.
Population characteristics	Population characteristics were derived from the source datasets and summarized where available, including age and sex. Dataset composition and imaging acquisition characteristics are reported in the manuscript and Supplementary Tables to describe heterogeneity across cohorts and protocols.
Recruitment	Participants were not recruited for this study. This work analyzes imaging data previously collected by existing cohorts and institutional datasets, accessed under the relevant agreements and approvals.
Ethics oversight	This study was conducted in accordance with the Declaration of Helsinki and relevant regulations. Analyses involving institution-specific datasets were approved by the University of California, San Francisco (UCSF) Institutional Review Board under the approvals listed in the manuscript; participants provided written informed consent under the originating study protocols. Osteoarthritis Initiative (OAI) data were accessed via the OAI data portal under registration and data use agreement; OAI procedures were approved by the relevant institutional review boards and are documented by the originating study. This work did not involve new participant recruitment, intervention, or prospective data collection.

Note that full information on the approval of the study protocol must also be provided in the manuscript.

Field-specific reporting

Please select the one below that is the best fit for your research. If you are not sure, read the appropriate sections before making your selection.

☒ Life sciences ☐ Behavioural & social sciences ☐ Ecological, evolutionary & environmental sciences

For a reference copy of the document with all sections, see nature.com/documents/nr-reporting-summary-flat.pdf

Life sciences study design

All studies must disclose on these points even when the disclosure is negative.

Sample size	Sample size was determined by the available datasets and prespecified splits. Exact sample sizes are reported in the manuscript and relevant tables and figure legends.
Data exclusions	Exclusion criteria were not defined as a single scan-level filter for all datasets. This work uses previously curated datasets with study-specific inclusion criteria defined by the originating cohorts where applicable. In our pipeline, exclusions primarily occurred at the slice or annotation level during quality control, for example when an annotation was missing, incomplete, or did not meet basic integrity checks for a given task. The relevant quality control procedures are described in the Methods and Supplementary Engineering Framework.
Replication	Performance and biomarker fidelity were evaluated on held-out test data across multiple datasets and tasks. Consistency was assessed across

Replication	anatomy-specific settings and multiple endpoints, including segmentation metrics and biomarker agreement. No new external cohort was collected for prospective replication.
Randomization	Randomization to experimental groups is not applicable because this is a secondary analysis of observational imaging datasets. Data were partitioned into prespecified splits to prevent leakage, as described in the Methods.
Blinding	Blinding is not applicable for this computational study. Evaluation was performed using predefined scripts and metrics applied to held-out data, as described in the Methods.

Reporting for specific materials, systems and methods

We require information from authors about some types of materials, experimental systems and methods used in many studies. Here, indicate whether each material, system or method listed is relevant to your study. If you are not sure if a list item applies to your research, read the appropriate section before selecting a response.

Materials & experimental systems

n/a	Involved in the study	
☒	☐	Antibodies
☒	☐	Eukaryotic cell lines
☒	☐	Palaeontology and archaeology
☒	☐	Animals and other organisms
☐	☒	Clinical data
☒	☐	Dual use research of concern
☒	☐	Plants

Methods

n/a	Involved in the study	
☒	☐	ChIP-seq
☒	☐	Flow cytometry
☒	☐	MRI-based neuroimaging

Clinical data

Policy information about clinical studies

All manuscripts should comply with the ICMJE guidelines for publication of clinical research and a completed CONSORT checklist must be included with all submissions.

Clinical trial registration	Not applicable. This work is a retrospective secondary analysis of existing cohort data and is not a clinical trial.
Study protocol	Not applicable for this analysis. The originating cohort documentation is cited in the manuscript.
Data collection	No new clinical data were collected for this study. OAI imaging and associated cohort variables were accessed via the OAI data portal under registration and data use agreement. Additional institution-specific datasets were accessed under institutional approvals and data governance as described in the manuscript.
Outcomes	Primary endpoints include segmentation performance and biomarker agreement between automated measurements and expert-derived references. Downstream outcomes include time-to-event knee replacement and incident osteoarthritis, evaluated using landmark-based prediction models as described in the manuscript.

Plants

Seed stocks	*Report on the source of all seed stocks or other plant material used. If applicable, state the seed stock centre and catalogue number. If plant specimens were collected from the field, describe the collection location, date and sampling procedures.*
Novel plant genotypes	*Describe the methods by which all novel plant genotypes were produced. This includes those generated by transgenic approaches, gene editing, chemical/radiation-based mutagenesis and hybridization. For transgenic lines, describe the transformation method, the number of independent lines analyzed and the generation upon which experiments were performed. For gene-edited lines, describe the editor used, the endogenous sequence targeted for editing, the targeting guide RNA sequence (if applicable) and how the editor was applied.*
Authentication	*Describe any authentication procedures for each seed stock used or novel genotype generated. Describe any experiments used to assess the effect of a mutation and, where applicable, how potential secondary effects (e.g. second site T-DNA insertions, mosiacism, off-target gene editing) were examined.*
